# Circulating HBV RNA and hepatitis B core-related antigen as determinants of HBsAg loss in persons with HIV in Europe

**DOI:** 10.1016/j.jhepr.2025.101671

**Published:** 2025-11-07

**Authors:** Lorin Begré, Anders Boyd, Marie-Laure Plissonnier, Barbara Testoni, Charles Béguelin, Franziska Suter-Riniker, Caroline Scholtès, Jürgen K. Rockstroh, Karine Lacombe, Lars Peters, Marintha Heil, Massimo Levrero, Andri Rauch, Fabien Zoulim, Gilles Wandeler, for the Swiss HIV Cohort Study, EuroSIDA and French HIV/HBV and Biliver cohorts, Irene A. Abela, Karoline Aebi-Popp, Alexia Anagnostopoulos, Manuel Battegay, Enos Bernasconi, Dominique L. Braun, Heiner C. Bucher, Alexandra Calmy, Matthias Cavassini, Angela Ciuffi, Günter Dollenmaier, Matthias Egger, Luisa Elzi, Jan S. Fehr, Jacques Fellay, Hansjakob Furrer, Christoph A. Fux, Huldrych F. Günthard, Anna Hachfeld, David Hans-Ulrich Haerry, Barbara Hasse, Hans H. Hirsch, Matthias Hoffmann, Irene Hösli, Michael Huber, David Jackson-Perry, Christian R. Kahlert, Olivia Keiser, Thomas Klimkait, Roger D. Kouyos, Helen Kovari, Katharina Kusejko, Niklaus D. Labhardt, Karoline Leuzinger, Begoña Martinez de Tejada, Catja Marzolini, Karin J. Metzner, Nicolas Müller, Johannes Nemeth, Dunja Nicca, Julia Notter, Paolo Paioni, Giuseppe Pantaleo, Matthieu Perreau, Andri Rauch, Luisa Paola Salazar-Vizcaya, Patrick Schmid, Olivier Segeral, Speck R. F, Marcel Stöckle, Philip E. Tarr, Alexandra Trkola, Gilles Wandeler, Maja Weisser, Sabine Yerly, Members of the EuroSIDA Study Group, A. Harxhi, M. Losso, M. Kundro, M. Knappik, P. Cichon, Klinik Penzing, M. Sarcletti, I. Karpov, V.M. Mitsura, D. Paduto, N. Clumeck, S. De Wit, M. Delforge, M. Frankenhuijsen, M. De Scheerder, J. Topalovic, J. Begovac, D. Jilich, L. Machala, D. Sedlacek, T. Benfield, J. Gerstoft, A.M. Lebech, O. Kirk, I.S. Johansen, L. Ostergaard, L. Wiese, L.N. Nielsen, K. Zilmer, I. Aho, J.-P. Viard, K. Lacombe, C. Pradier, E. Fontas, C. Duvivier, J. Rockstroh, O. Degen, C. Hoffmann, C. Stefan, J. Bogner, C. Lehmann, A. Abutidze, H. Sambatakou, G. Adamis, N. Paissios, J. Szlávik, M. Gottfredsson, E. Devitt, L. Tau, O.A. Bondarenko, L.M. Wattad, H. Elinav, D. Elbirt, G. Marchetti, G. Guaraldi, C. Mussini, A. Castagna, A. Ridolfo, F. Schiavo, V. Uzdaviniene, R. Matulionyte, T. Staub, R. Batutu, M. vd Valk, J. Trajanovska, D.H. Reikvam, B. Knysz, B. Szetela, M. Inglot, E. Bakowska, M. Parczewski, B. Aksak-Was, M. Beniowski, E. Mularska, E. Jablonowska, J. Kamerys, K. Wojcik, I. Mozer-Lisewska, B. Rozplochowski, A. Zagalo, K. Mansinho, F. Maltez, R. Radoi, C. Oprea, D. Gusev, T. Trofimova, E. Kuzovatova, E. Borodulina, E. Vdoushkina, J. Ranin, J. Tomazic, E. Martínez, J.M. Miró, M. Laguno, J.L. Blanco, M. Martinez-Rebollar, J. Ambrosioni, B. Torres, L. de la Mora, A. Gonzalez-Cordon, I. Chivite, A. Foncillas, E. de Lazzaari, L. Berrocal, P. Callau, A. Inciarte, J. Alcami, J. Mallolas, R. Paredes, J. Puig, J.R. Santos, C. Miranda, P. Domingo, G.M. Mar, M.G. Gracia, E.S. De A Arroniz, A. Ponz, C. Carlander, A. Sönnerborg, J. Brännström, K. Falconer, F. Månsson, K. Kusejko, D. Braun, M. Cavassini, A. Calmy, H. Furrer, M. Battegay, P. Schmid, E. Bernasconi, A. Kuznetsova, L. Hetman, A. Kryshchuk, M. Boffito, S. Edwards, F. Burns, C. Orkin, A. Winston, A. Clarke, C. Mackintosh, C. Boesecke, C. Carlander, C. Pradier, C. Oprea, E. Martinez, G. Wandeler, I. Aho, I. Karpov, J. Begovac, J.D. Kowalska, J. Lundgren, L.D. Rasmussen, L. Tau, O. Nesterova, R. Matulionyte, S. Nozza, J.D. Kowalska, L. Peters, L. Peters, J.F. Larsen, M. Gardizi, N. Jaschinski, A. Timiryasova, B. Neesgaard, F. Roper, D. Raben, A.H. Fischer, A. Cozzi-Lepri, W. Bannister, T.W. Elsing, L.R. Kumar, S. Shahi, B. Pepa, Members of the French HIV/HBV and Biliver cohorts, Anders Boyd, Patrick Miailhes, Caroline Lascoux-Combe, Julie Chas, Pierre-Marie Girard, Joël Gozlan, Fabien Zoulim, Constance Delaugerre, Hayette Rougier, Karine Lacombe

**Affiliations:** 1 Department of Infectious Diseases, Inselspital, Bern University Hospital, University of Bern, Bern, Switzerland; 2 Graduate School for Health Sciences, University of Bern, Bern, Switzerland; 3 Stichting HIV Monitoring, Amsterdam, The Netherlands; 4 Department of Infectious Diseases, Amsterdam UMC Location University of Amsterdam, Amsterdam, The Netherlands; 5 Department of Infectious Diseases, Amsterdam Institute for Infection and Immunity, Amsterdam, The Netherlands; 6 UMR PaThLiv U1350, Inserm, Université Claude Bernard Lyon 1, Lyon, France; 7 Lyon Hepatology Institute, IHU EVEREST, Lyon, France; 8 Institute for Infectious Diseases, University of Bern, Bern, Switzerland; 9 Department of Virology, Hospices Civils de Lyon, Lyon, France; 10 HIV-Clinic, Department of Medicine I, University Hospital Bonn, Bonn, Germany; 11 Sorbonne Université, Inserm, Institut Pierre Louis d'Épidémiologie et de Santé Publique, IPLESP, Paris, France; 12 AP-HP, GH Sorbonne.Université, Hôpital Saint-Antoine, Service de maladies infectieuses et tropicales, Paris, France; 13 CHIP, Centre of Excellence for Health, Immunity and Infections, Rigshospitalet, Copenhagen, Denmark; 14 Roche Molecular Diagnostics, Pleasanton, CA, USA; 15 Department of Hepatology, Hospices Civils de Lyon, Lyon, France; 16 Institute of Social and Preventive Medicine, University of Bern, Bern, Switzerland

**Keywords:** Hepatitis B virus, HIV, Coinfection, HBV RNA, Hepatitis B core-related antigen, Cohort studies, Tenofovir

## Abstract

**Background & Aims:**

HBsAg loss improves clinical outcomes in persons with HIV and HBV coinfection. We aimed to evaluate if hepatitis B core-related antigen and circulating HBV RNA levels were associated with HBsAg loss in Euro-B, a multi-cohort collaboration including data from the Swiss HIV Cohort Study, EuroSIDA, and the French HIV/HBV cohort.

**Methods:**

We included persons with HIV, a positive HBsAg, and ≥6 months of follow-up on tenofovir-containing antiretroviral therapy. We evaluated quantitative HBsAg, HBV DNA, hepatitis B core-related antigen, and HBV RNA levels over time and assessed HBsAg loss (*i.e.* quantitative HBsAg <0.05 IU/ml) during tenofovir therapy.

**Results:**

Among 599 participants median age was 41 years (IQR 35–47), 18.4% were female and 47.3% HBeAg-positive. We observed HBsAg loss in 12.9% of participants after 2 years and in 18.2% during a median follow-up of 8.2 years (IQR 3.6–13.1). Individuals who were HBeAg-negative were more likely to have a negative hepatitis B core-related antigen and HBV RNA below the detection limit than participants who were HBeAg-positive. Quantitative HBsAg ≤1,000 IU/ml at baseline was the strongest predictor of HBsAg loss regardless of HBeAg status. Additionally, HBsAg loss was associated with lower baseline HBV RNA levels (odds ratio 0.66, 95% CI 0.49–0.88) and higher baseline HBV DNA levels in participants who were HBeAg-positive.

**Conclusions:**

In this European cohort of persons with HIV/HBV, 18% experienced HBsAg loss during tenofovir-containing antiretroviral therapy. In addition to low baseline quantitative HBsAg levels, HBV RNA may predict HBsAg loss in individuals who are HBeAg-positive.

**Impact and implications:**

The present study builds on a multi-cohort collaboration including persons with HIV/HBV from Europe. It provides estimates on the probability of HBsAg loss during long-term tenofovir-containing antiretroviral therapy and describes the potential of the novel biomarkers HBV RNA and hepatitis B core-related antigen as its predictors. The discrepancies regarding HBV RNA and HBcrAg levels before and during therapy observed between persons who were HBeAg-negative and HBeAg-positive with HIV/HBV may influence treatment decisions and the development of new treatment strategies.

**Clinical Trials Registration:**

The study is registered at ClinicalTrials.gov (NCT04984772).

## Highlights

• Of the 599 participants, 18.2% experienced HBsAg loss within a median of 8.2 years.
• HBV RNA and HBcrAg levels decreased during therapy independent of HBsAg loss.
• Most participants who were HBeAg-negative reached negative HBV RNA and HBcrAg levels.
• Low quantitative HBsAg levels were the strongest predictor of HBsAg loss.
• In people who were HBeAg-positive, lower HBV RNA levels were also associated with HBsAg loss.

## Graphical abstract

**Figure:**
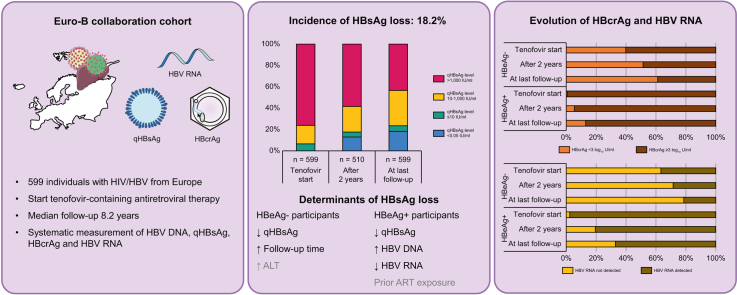

## Introduction

Worldwide, ∼8% of persons with HIV (PWH) are living with chronic HBV infection and the risk of liver-related events and death is higher in this population compared with persons without HIV.[1], [2], [3] Despite the suppression of HBV viral replication in the majority of persons treated with tenofovir disoproxil fumarate (TDF) or tenofovir alafenamide (TAF) as part of antiretroviral therapy (ART), the risk of developing liver-related events, including hepatocellular carcinoma (HCC), remains elevated.^4^^,^^5^ HBsAg loss improves clinical outcomes but occurs infrequently.^6^^,^^7^ Several studies found higher rates of HBsAg loss in PWH compared with persons with HBV monoinfection, but the reasons for this difference remains poorly understood.^8^

Measuring intrahepatic markers of HBV activity would be ideal for assessing viral activity and would improve our understanding of the determinants of HBsAg loss. However, the invasive nature of liver biopsy reduces its widespread use. Serum quantitative HBsAg (qHBsAg) is strongly correlated with levels of replication within hepatocytes. Furthermore, low qHBsAg levels at the start of antiviral therapy were found to be associated with HBsAg loss in persons with and without HIV.^9^^,^^10^ In a case-control study within the Swiss HIV Cohort Study (SHCS), we found that most persons who experienced HBsAg loss had an early decline in qHBsAg levels after starting tenofovir therapy, but individual trajectories were diverse.^11^ In contrast, individuals without HBsAg loss consistently had stable levels during long-term treatment with tenofovir. Serum HBcrAg, a composite of the precore/core gene products of hepatitis B core antigen, HBeAg, and p22 core-related protein, reflects the size of the transcriptionally active covalently closed circular DNA (cccDNA) pool in the liver.[12], [13], [14] Circulating HBV RNA seems to consist mainly of pregenomic RNA and reflects cccDNA transcriptional activity.[15], [16], [17] HBcrAg and HBV RNA would therefore be potential candidates to help predict HBsAg loss.

We aimed to evaluate the association of HBcrAg and HBV RNA levels with HBsAg loss during treatment with tenofovir-containing ART in Euro-B, an international multi-cohort collaboration including data from persons with HIV/HBV from the SHCS, the EuroSIDA Study, and the French HIV/HBV cohort.

## Patients and methods

### Study design and population

We conducted a longitudinal, observational cohort study using data from the SHCS, the EuroSIDA Study, and the French HIV/HBV and Biliver cohorts.[18], [19], [20] We considered participants of these cohorts who were aged 16 years or older, had a positive HBsAg test, and commenced TDF or TAF-containing ART at some point during follow-up. To avoid double inclusion of Swiss participants, participants from EuroSIDA followed in Swiss centers were not included in the EuroSIDA participants. In this study, participants needed to have a qHBsAg ≥0.05 IU/ml at tenofovir commencement and a qHBsAg measurement after >6 months of follow-up on tenofovir. We excluded participants with unknown date of tenofovir start and with an incident HBV infection documented after the start of the first tenofovir-containing ART regimen. Participants could switch between TDF and TAF during follow-up.

We defined baseline as the start date of the first tenofovir-containing ART regimen and follow-up continued to the last available stored plasma sample, death, loss to follow-up, cessation of tenofovir, or database closure on 31 October 2022, whichever occurred first. Follow-up continued in case of interruption of tenofovir therapy when participants resumed therapy any time later on. We measured qHBsAg, HBV DNA, HBcrAg, and HBV RNA using stored plasma samples at baseline (with a window period of -12 to +6 months), 2 years after tenofovir start (with a window period of ±6 months) and at the last time point with available data. Detailed information on demographic, clinical, and laboratory data were collected according to the standardized study protocols of the respective cohorts. Local ethical committees approved the cohort studies (SHCS: Cantonal Ethics Committee Zurich, BASEC-No. 2023-02080; EuroSIDA: Regional Committees on Health Research Ethics for the Capital Region of Denmark, No. H-3-2012-049; and French HIB/HBV and Biliver cohorts: Ethics Committees of the Pitié-Salpêtrière Hospital and the Saint-Antoine Hospital in Paris, France) and written consent was obtained from all participants according to local regulations. The study is registered at ClinicalTrials.gov (NCT04984772).

### Outcomes and definitions

The primary outcome was the proportion of participants with HBsAg loss, defined as a qHBsAg <0.05 IU/ml, at the last follow-up visit. Secondary outcomes were the proportion of participants with HBsAg loss at 2 years of tenofovir therapy, and the proportion with HBsAg seroreversion, defined as a qHBsAg ≥0.05 IU/ml at the last follow-up visit after having achieved a qHBsAg <0.05 IU/ml at 2 years. Other secondary outcomes included the proportion with HBV DNA suppression, negative HBcrAg and HBV RNA levels after 2 years of follow-up and at the last follow-up. HBV DNA suppression was defined as <20 IU/ml, a negative HBcrAg as <3 log_10_ U/ml, and an undetectable HBV RNA level as HBV RNA below the lower limit of detection (LLOD). Cirrhosis was assessed by liver biopsy, transient elastography >11 kPa, or APRI (aspartate aminotransferase-to-platelet ratio index) >2, as described previously.^21^

### Laboratory analyses

We quantified qHBsAg using a commercial chemiluminescent microparticle immunoassay, either the Elecsys HBsAg II assays (Roche Diagnostics, Rotkreuz, Switzerland) or the ARCHITECT HBsAg (Abbott, Sligo, Ireland) with a sensitivity of ≤0.05 IU/ml. HBV DNA was measured using the commercial quantitative nucleic acid test COBAS HBV on the COBAS 4800 system (Roche Diagnostics, Rotkreuz, Switzerland) with a LLOD of 4.4 IU/ml and a linear range from 10 to 1 × 10^9^ IU/ml. We quantified HBcrAg with the Lumipulse G HBcrAg assay on the LUMIPULSE G1200 Analyzer (Fujirebio Europe, Gent, Belgium) according to the manufacturer’s instructions. As proposed by Kimura *et al.*^12^, HBcrAg levels <3 log_10_ U/ml were considered negative. We determined HBV RNA levels using the COBAS HBV RNA automated investigational assay on the COBAS 6800 system (Roche Molecular Diagnostics, Pleasanton, CA, USA) with a LLOD <5 copies/ml (cp/ml) and a linear range between 10 and 10^7^ cp/ml, as described previously.^22^

### Statistical analysis

We compared baseline characteristics between participants who were HBeAg-positive and HBeAg-negative using Pearson’s χ^2^ and Wilcoxon rank-sum tests, where appropriate. We described qHBsAg, HBV DNA, HBcrAg, and HBV RNA levels at the different time points, stratified by HBeAg status. We assessed predictors of HBsAg loss after 2 years of tenofovir therapy and at the latest follow-up time point using multivariable logistic regression models, stratified by HBeAg status at baseline. Because of collinearity between HBcrAg and HBV RNA levels, we evaluated these predictors in two separate regression models. We adjusted the models for sex at birth, age, CD4 T cell count, previous ART, qHBsAg levels, HBV DNA levels, alanine aminotransferase (ALT) levels and either HBcrAg or HBV RNA levels at start of tenofovir. To adjust for different follow-up durations between participants, we included log_10_ transformed follow-up time in the logistic regression models evaluating predictors of HBsAg loss at the last follow-up time point.

Statistical significance was defined as a two-sided *p* value <0.05. We performed all analyses using Stata/MP 16.1 (StataCorp, College Station, TX, USA).

## Results

### Patient characteristics

Of 1,125 participants with an HBsAg-positive test before commencing tenofovir, 599 participants (53.2%) had a qHBsAg ≥0.05 IU/ml at start of tenofovir and a qHBsAg measurement after >6 months of follow-up, and hence included in the primary analysis (Fig. S1). Participants included in the analysis were more likely to have been born in a country with an HBV prevalence ≥2% and drank less alcohol compared with those not included (Table S1). Of the included participants, 387 were from the SHCS (64.6%), 119 (19.9%) from EuroSIDA, and 93 (15.5%) from the French HIV/HBV cohort. Median age was 41 years (IQR 35–47), 110/599 (18.4%) were assigned female at birth and 279/590 (47.3%) were HBeAg-positive (Table 1). When commencing tenofovir therapy, 345/599 (57.6%) were already on ART and 289/599 (48.2%) received ≥1 anti-HBV active drug including lamivudine, emtricitabine, entecavir, adefovir, or interferon-α. Among 192 participants with available information on HBV genotype, 143 (74.3%) had genotype A infection. HBeAg-positive participants commenced tenofovir therapy earlier (median calendar year 2004, IQR 2003–2007 *vs.* 2007, IQR 2004–2010) and were more likely to be male (91.4% *vs.* 72.7%), to be born in a country with low HBV prevalence (82.7% *vs.* 59.9%), to have experienced an AIDS-defining condition (32.3% *vs.* 19.3%), and had higher ALT levels (median 54 IU/ml, IQR 37–86 *vs.* 33 IU/ml, IQR 22–57) compared with participants who were HBeAg-negative. At baseline, participants who were HBeAg-positive had higher median HBV DNA, qHBsAg, HBcrAg, and HBV RNA levels. Participants who were HBeAg-negative and participants who were HBeAg-positive and already on ART before starting tenofovir had higher CD4 counts, CD4 nadir, and were less likely to have experienced an AIDS-defining condition (Table S2). Compared with those not on ART, participants who were HBeAg-negative on ART had lower HBV DNA levels before starting tenofovir, whereas individuals who were HBeAg-positive with previous ART had higher qHBsAg levels, but similar HBV DNA levels.

**Table 1:** Characteristics of participants at baseline, stratified by HBeAg status.

	Total	HBeAg-negative	HBeAg-positive	*p* value
	N = 599	n = 311	n = 279	
Female sex at birth	110/599 (18.4)	85/311 (27.3)	24/279 (8.6)	**<0.001**
Age, median (IQR), years	41.0 (35.0–47.0)	41.0 (35.0–47.0)	41.0 (36.0–46.0)	0.68
Calendar year of tenofovir start, median (IQR)	2006 (2003–2009)	2007 (2004–2010)	2004 (2003–2007)	**<0.001**
HBV prevalence ≥2% in country of origin∗	174/596 (29.2)	124/309 (40.1)	48/278 (17.3)	**<0.001**
Mode of HIV acquisition				**<0.001**
Men who have sex with men	309/599 (51.6)	117/311 (37.6)	187/279 (67.0)	
Heterosexual contact	166/599 (27.7)	115/311 (37.0)	47/279 (16.8)	
Injection drug use	76/599 (12.7)	49/311 (15.8)	27/279 (9.7)	
Other or unknown	48/599 (8.0)	30/311 (9.6)	18/279 (6.5)	
BMI, median (IQR), kg/m^2^	22.8 (20.8–25.4)	23.2 (21.1–26.2)	22.6 (20.7–24.3)	**0.003**
On ART	345/599 (57.6)	170/311 (54.7)	173/279 (62.0)	0.07
On lamivudine or emtricitabine	286/599 (47.7)	138/311 (44.4)	146/279 (52.3)	0.05
On adefovir or entecavir	7/599 (1.2)	3/311 (1.0)	4/279 (1.4)	0.60
On interferon-α therapy	4/599 (0.7)	4/311 (1.3)	0/279 (0.0)	0.06
CD4 count, median (IQR), cells/mm^3^	355 (230–533)	372 (237–533)	347 (222–547)	0.76
CD4 nadir, median (IQR), cells/mm^3^	170 (61–290)	183 (72–290)	142 (50–291)	**0.04**
HIV viral load <50 copies/ml	300/599 (50.1)	154/311 (49.5)	142/279 (50.9)	0.74
AIDS-defining condition†	151/599 (25.2)	60/311 (19.3)	90/279 (32.3)	**<0.001**
Liver cirrhosis	50/366 (13.7)	28/196 (14.3)	22/165 (13.3)	0.79
Ever unhealthy alcohol use	41/292 (14.0)	21/151 (13.9)	19/137 (13.9)	0.99
HBV DNA, median (IQR), log_10_ IU/ml	3.2 (0.8–6.7)	1.3 (0.0–2.9)	6.6 (4.0–8.0)	**<0.001**
HBV DNA <20 IU/ml	177/596 (29.7)	155/309 (50.2)	21/278 (7.6)	
qHBsAg, median (IQR), log_10_ IU/ml	3.9 (3.1–4.6)	3.4 (2.3–3.9)	4.6 (3.9–5.0)	**<0.001**
qHBsAg >1,000 IU/ml	456/599 (76.1)	199/311 (64.0)	250/279 (89.6)	
qHBsAg >10–1,000 IU/ml	104/599 (17.4)	76/311 (24.4)	26/279 (9.3)	
qHBsAg ≤10 IU/ml	39/599 (6.5)	36/311 (11.6)	3/279 (1.1)	
HBcrAg, median (IQR), log_10_ U/ml	5.9 (3.1–8.0)	3.3 (2.9–4.3)	8.0 (7.2–8.5)	**<0.001**
HBcrAg <3 log_10_ U/ml	126/587 (21.5)	121/304 (39.8)	2/274 (0.7)	
HBV RNA, median (IQR), log_10_ copies/ml	2.8 (0.0–5.9)	0.0 (0.0–0.8)	5.9 (5.1–6.3)	**<0.001**
HBV RNA <LLOD	197/566 (34.8)	188/297 (63.3)	6/260 (2.3)	
Ever Hepatitis D antibody positive	72/539 (13.4)	54/276 (19.6)	18/254 (7.1)	**<0.001**
Ever Hepatitis D RNA positive	40/534 (7.5)	29/271 (10.7)	11/254 (4.3)	**0.006**
Hepatitis C antibody positive	65/531 (12.2)	42/283 (14.8)	23/248 (9.3)	0.05
Hepatitis C RNA positive	34/524 (6.5)	23/276 (8.3)	11/239 (4.6)	0.09
ALT elevation ≥5 × ULN	38/586 (6.5)	14/304 (4.6)	24/273 (8.8)	**0.04**

∗ Based on 2015 estimates from GBD 2019 Hepatitis Collaborators, Lancet Gastroenterol Hepatol 2022; 7:796–829.

† According to the clinical classification of HIV disease by the US Centers for Disease Control and Prevention. Data are presented as median (IQR) for continuous measures, and n/N (%) for categorical measures. Continuous variables were compared using Wilcoxon rank-sum tests. Comparisons of categorical variables were performed using Pearson's χ^2^ tests. Level of significance: *p* <0.05 (bold). AIDS, acquired immunodeficiency syndrome; ALT, alanine aminotransferase; ART, antiretroviral therapy; BMI, body mass index; HBcrAg, hepatitis B core-related antigen; HBeAg, hepatitis B e antigen; HBV, hepatitis B virus; LLOD, lower limit of detection; qHBsAg, quantitative hepatitis B surface antigen; RNA, ribonucleic acid, ULN, upper limit of normal; XTC, lamivudine or emtricitabine.

### HBsAg loss after 2 years and at the last follow-up visit

**Fig. 1:**
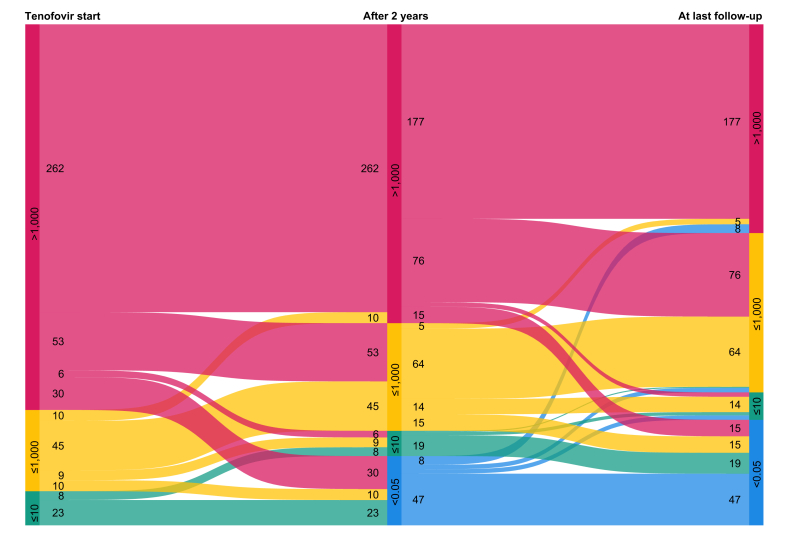
**Alluvial plot showing qHBsAg levels during follow-up on tenofovir-containing antiretroviral therapy among the 456 (76.1%) participants with three available measurements.** Red includes participants with qHBsAg >1,000 IU/ml, yellow those with >10–1,000 IU/ml, green those with 0.05–10 IU/ml, and blue those with HBsAg loss, defined as qHBsAg <0.05 IU/ml. qHBsAg, quantitative hepatitis B surface antigen.

At baseline, 456 (76.1%) participants had qHBsAg >1,000 IU/ml. As shown in Fig. 1, this proportion declined to 298/510 (58.4%) after 2 years and 260 (43.4%) at the last follow-up visit after a median duration of 8.2 years (IQR 3.6–13.1). After 2 years of treatment, 66/510 (12.9%) participants had a qHBsAg <0.05 IU/ml, and 109/599 (18.2%) had experienced HBsAg loss at the last available follow-up visit. During follow-up, 62 (10.4%) participants died, 77 (12.9%) discontinued tenofovir therapy permanently, and 81 (13.5%) were lost to follow-up, no longer seeking HIV care at a participating study center or withdrew consent. Rates of HBsAg loss after 2 years of tenofovir therapy were similar in participants who were HBeAg-negative (37/261, 14.2%) and HBeAg-positive (27/240, 11.3%, *p* = 0.33), but those who were HBeAg-negative tended to be more likely to achieve HBsAg loss at the last follow-up visit (66/311, 21.2%) than those who were HBeAg-positive (42/279, 15.1%, *p* = 0.05).

### Changes in qHBsAg, HBV DNA, HBcrAg, and HBV RNA levels during follow-up according to HBeAg status

**Fig 2:**
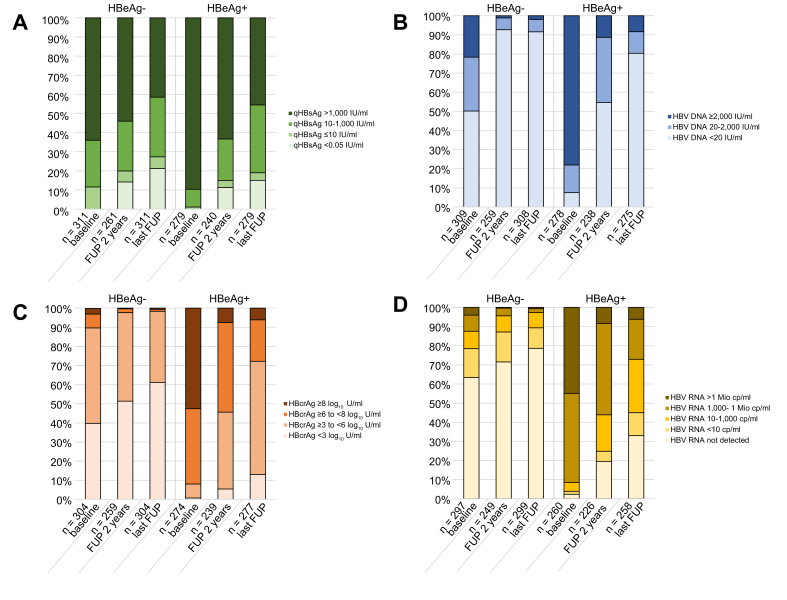
**qHBsAg (A), HBV DNA (B), HBcrAg (C), and HBV RNA (D) levels in HBeAg-negative and HBeAg-positive participants at start of tenofovir therapy, after 2 years of follow-up and at the last follow-up visit after a median follow-up of 8.2 years (IQR 3.6-13.1).** cp/ml, copies per milliliter; FUP, follow-up; HBcrAg, hepatitis B core-related antigen; HBeAg-, hepatitis B e antigen negative; HBeAg+, hepatitis B e antigen positive; Mio, million; qHBsAg, quantitative hepatitis B surface antigen; HBV, hepatitis B virus.

At baseline, 112/311 (36.0%) HBeAg-negative and 29/279 (10.4%) HBeAg-positive participants had qHBsAg ≤1,000 IU/ml. During follow-up, these proportions increased to 58.5% (182/311) in HBeAg-negative and 54.5% (152/279) in HBeAg-positive participants (Fig. 2A). Participants who were HBeAg-positive with anti-HBV active therapy before tenofovir were more likely to be classified into higher qHBsAg level categories after 2 years (*p* = 0.003) and at the last follow-up (*p* = 0.001) compared with those not on ART, whereas in participants who were HBeAg-negative qHBsAg levels were similar in both sub-groups after 2 years (*p* = 0.30) and at the last follow-up (*p* = 0.62, Fig. S2). HBV DNA was undetectable at baseline in 50.2% (155/309) of participants who were HBeAg-negative compared with 7.6% (21/278) of those who were HBeAg-positive (Fig. 2B). Participants who were HBeAg-negative were also more likely to be HBcrAg-negative compared with those who were HBeAg-positive from baseline (39.8% *vs.* 0.7%) until the last follow-up visit (61.2% *vs.* 13.0%, Fig. 2C), and to have HBV RNA <LLOD from baseline (63.3% *vs.* 2.3%) to the last follow-up visit (78.6 *vs.* 33.0%, Fig. 2D). In participants who were HBeAg-negative, HBV DNA, HBcrAg, and HBV RNA level categories were similar between participants with and without anti-HBV active therapy before tenofovir during follow-up (Fig. S3–S5). In contrast, participants who were HBeAg-positive on anti-HBV active therapy at baseline had higher HBcrAg (median 6.6 log_10_ U/ml, IQR 5.2–7.2) and HBV RNA (median 3.7 log_10_ cp/ml, IQR 2.1–4.9) levels after 2 years compared with those not on therapy with anti-HBV activity (HBcrAg: median 5.9 log_10_ U/ml, IQR 4.3–7.2, *p* = 0.03; HBV RNA: median 2.7 log_10_ cp/ml, IQR 0.0–4.9, *p* = 0.04). Those differences remained at the last follow-up (HBcrAg: median 5.1 log_10_ U/ml, IQR 4.1–6.5 *vs.* median 4.5 log_10_ U/ml, IQR 3.4–5.6, *p* <0.001; HBV RNA: median 1.8 log_10_ cp/ml, IQR 0.8–3.8 *vs.* median 0.8 log_10_ cp/ml, IQR 0.0–2.8, *p* = 0.004).

**Fig. 3:**
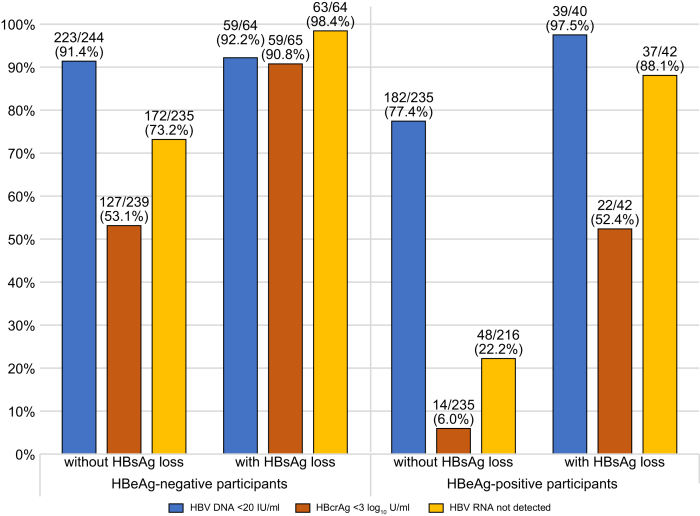
**Proportion of participants with HBV DNA <20 IU/ml, HBcrAg <3 log_10_ U/ml and HBV RNA below the detection limit at the last follow-up time point, stratified by HBsAg loss and HBeAg status.** HBcrAg, hepatitis B core-related antigen; HBeAg, hepatitis B e antigen; HBsAg, hepatitis B surface antigen.

In participants who were HBeAg-negative with HBsAg loss, 59/65 (90.8%) were HBcrAg-negative and 63/64 (98.4%) had HBV RNA <LLOD at the last follow-up visit. In comparison, 52.4% (22/42) of participants who were HBeAg-positive with HBsAg loss were HBcrAg-negative and 88.1% (37/42) had HBV RNA <LLOD (Fig. 3) at the last follow-up visit. At the last follow-up visit, a combination of negative HBcrAg and HBV RNA <LLOD was observed in 90.6% (58/64) of participants who were HBeAg-negative compared with 47.6% (20/42) of those who were HBeAg-positive. As shown in Fig. S6, these proportions were comparable at the 2-year follow-up time point. At 2 years, combined suppressed levels of HBcrAg and HBV RNA were observed in 75.0% (27/36) of participants who were HBeAg-negative and 33.3% (8/24) of those who were HBeAg-positive.

### Predictors of HBsAg loss

**Fig. 4:**
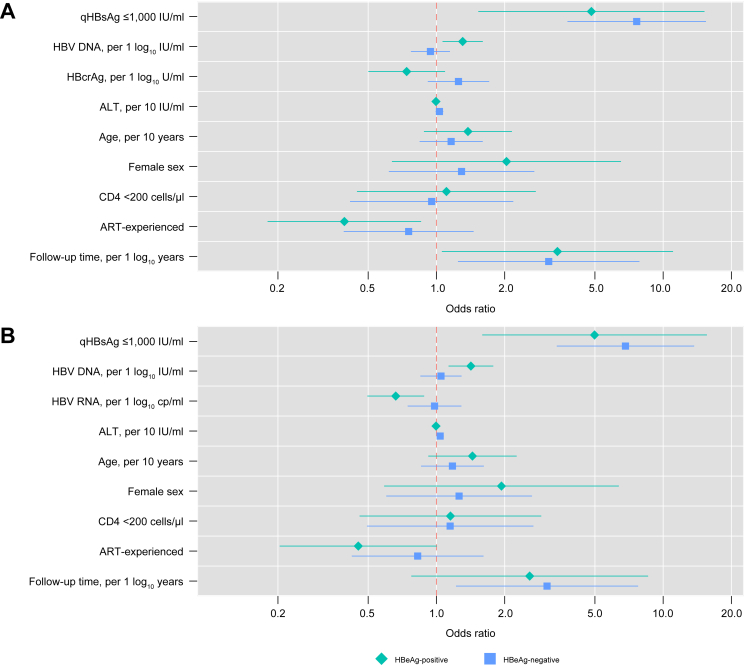
**Multivariable logistic regression models for HBsAg loss at the last follow-up time point.** (A) with HBcrAg included in the model, (B) with HBV RNA included in the model. Except follow-up time, all parameters included in the models are baseline values. Points represent the odds ratios and bands the 95% CIs. Green refers to the model restricted to participants who were HBeAg-positive and blue to the model restricted to those who were HBeAg-negative. Wald tests were performed to assess statistical significance of individual covariables with significance defined as *p* <0.05. In the models including HBcrAg (panel A) the following variables were significantly associated with HBsAg loss in at least one sub-group: qHBsAg ≤1,000 IU/ml (HBeAg-negative participants *p* <0.001; HBeAg-positive participants *p* = 0.007), being ART-experienced (HBeAg-negative participants *p* = 0.40; HBeAg-positive participants *p* = 0.02), and follow-up time (HBeAg-negative participants *p* = 0.02; HBeAg-positive participants *p* = 0.04). In the models including HBV RNA (panel B) the following variables were significantly associated with HBsAg loss: qHBsAg ≤1,000 IU/ml (participants who were HBeAg-negative *p* <0.001; participants who were HBeAg-positive *p* = 0.006), HBV DNA (participants who were HBeAg-negative *p* = 0.67; participants who were HBeAg-positive *p* = 0.003), HBV RNA (participants who were HBeAg-negative *p* = 0.89; participants who were HBeAg-positive *p* = 0.005), ALT (participants who were HBeAg-negative *p* = 0.048; participants who were HBeAg-positive *p* = 0.71), and follow-up time (participants who were HBeAg-negative *p* = 0.02; participants who were HBeAg-positive *p* = 0.12). Actual odds ratios, 95% CIs and *p* values for all variables are depicted in Tables S3 and S4. ALT, alanine aminotransferase; ART, antiretroviral therapy; cp/ml, copies per milliliter; HBcrAg, hepatitis B core-related antigen; HBeAg, hepatitis B e antigen; qHBsAg, quantitative hepatitis B surface antigen.

In multivariable analysis, qHBsAg ≤1,000 IU/ml at baseline and follow-up duration were the strongest predictors of HBsAg loss at the last follow-up visit among participants who were HBeAg-negative (model with HBcrAg: odds ratio [OR] 7.64, 95% CI 3.78–15.44; model with HBV RNA: OR 6.82, 95% CI 3.39–13.70) and participants who were HBeAg-positive (model with HBcrAg: OR 4.82, 95% CI 1.53–15.21; model with HBV RNA: OR 4.98, 95% CI 1.59–15.58) (Fig. 4 and Tables S3 and S4). Among participants who were HBeAg-negative, neither HBcrAg, nor HBV RNA levels were associated with HBsAg loss after a median of 8.2 years of follow-up. However, higher ALT (OR 1.04, 95% CI 1.00–1.08 per 10 IU/ml increase) was associated with HBsAg loss in the multivariable model including HBV RNA but not in the one including HBcrAg. In individuals who were HBeAg-positive, lower HBV RNA (OR 0.66, 95% CI 0.50–0.88 per 1 log_10_ cp/ml increase) and higher HBV DNA levels were associated with HBsAg loss, whereas HBcrAg levels were not. Being ART-experienced was associated with HBsAg loss only in the multivariable model including HBcrAg and restricted to participants who were HBeAg-positive.

After 2 years of tenofovir therapy, HBsAg loss was associated with qHBsAg ≤1,000 IU/ml in participants who were HBeAg-negative (model with HBcrAg: OR 22.82, 95% CI 6.99–74.54; model with HBV RNA: OR 19.91, 95% CI 6.21–63.85), but not in participants who were HBeAg-positive (model with HBcrAg: OR 1.43, 95% CI 0.41–5.09; model with HBV RNA: OR 1.77, 95% CI 0.50–6.25) (Tables S5 and S6). In individuals who were HBeAg-negative, higher HBcrAg levels (OR 1.79, 95% CI 1.16–2.76 per 1 log_10_ U/ml increase) and female sex at birth were additionally associated with HBsAg loss after 2 years of therapy. Higher ALT levels were associated with HBsAg loss only in the model including HBV RNA in individuals who were HBeAg-negative. In participants who were HBeAg-positive, lower HBV RNA (OR 0.64, 95% CI 0.47–0.85 per 1 log_10_ cp/ml increase) and HBcrAg levels (OR 0.66, 95% CI 0.44–1.00 per 1 log_10_ U/ml increase) as well as prior ART was associated with HBsAg loss after 2 years.

In sensitivity analyses, we excluded all participants with detectable hepatitis C RNA at tenofovir start and participants who ever had replicating hepatitis D infection. As shown in Table S7, exclusion of the above-mentioned participants did not substantially influence our models. Similarly, our multivariable logistic regression models of predictors of HBsAg loss after 2 years of tenofovir therapy remained relatively unchanged (Table S8).

### HBsAg seroreversion

Among individuals with three available qHBsAg measurements, 16/63 (25.3%) participants who had qHBsAg <0.05 IU/ml after 2 years seroreverted to being HBsAg-positive at the last follow-up visit. Participants with seroreversion had higher qHBsAg levels (3.7 log_10_ IU/ml, IQR 3.3–4.4 *vs.* 1.4 log_10_ IU/ml, IQR 0.1–3.5, *p* = 0.01) and HBV RNA levels at baseline (4.9 log_10_ cp/ml, IQR 0.8–5.6 *vs.* 0.8 log_10_ cp/ml, IQR 0.0–4.8), *p* = 0.02) and were more likely to be HBeAg-positive (10/15, 67% *vs.* 16/46, 35%, *p* = 0.03). In addition, they were more likely to have a detectable HBV viral load (5/16, 31% *vs.* 1/45, 2%, *p* = 0.001) and detectable HBV RNA levels (6/16, 37.5% *vs.* 1/47. 2%, *p* <0.001) after 2 years of follow-up despite having qHBsAg <0.05 IU/ml (Table S9).

## Discussion

In this large prospective study including persons with HIV/HBV from three European cohorts, over 18% experienced HBsAg loss during a median of 8 years of tenofovir-containing ART. Participants with low qHBsAg levels were over five times more likely to experience HBsAg loss on tenofovir than those with higher levels. Lower HBV RNA levels in participants who were HBeAg-positive were additionally associated with HBsAg loss.

The proportion of HBsAg loss found in our study is consistent with data from the German HIV/HBV cohort, where 18% of participants experienced HBsAg loss during a median follow-up of 11 years.^23^ Similar proportions have also been described in other cohorts including participants from China, Thailand, Australia, and Zambia.[24], [25], [26] These findings, however, contrast with data from persons with HBV monoinfection, where usually <1% per year experience HBsAg loss.^7^^,^^8^ Previous studies have reported an association between lower CD4+ T cell counts or advanced HIV disease and increased rates of HBsAg loss, suggesting immune reconstitution as a driver of HBsAg loss.^9^^,^^27^ Although we did not observe such an association in our study, participants who were ART-naïve and HBeAg-positive were four times more likely to experience HBsAg loss within 2 years of tenofovir therapy compared with their ART-experienced counterparts. Interestingly, we did not observe an association between prior ART and HBsAg loss among HBeAg-negative participants. As partial recovery of the impaired HBV-specific T cell response in chronic HBV infection can be observed in persons without HIV, HBeAg-positive PWH – who probably acquired HBV relatively recently, compared with their HBeAg-negative counterparts – may be able to establish robust HBV-specific T cell responses and achieve immunological control of HBV with immune reconstitution after ART uptake.^28^^,^^29^

In line with our pilot study, which relied on a median number of 12 follow-up measurements, HBV RNA and HBcrAg levels decreased in participants with and without HBsAg loss during long-term tenofovir-containing ART.^30^ These findings contrast with a study including 95 persons with HIV/HBV from North America, where declining HBV RNA and HBcrAg levels were only observed in individuals who were HBeAg-positive.^31^ However, in that study almost all participants were on nucleos(t)ide analogue therapy at study inclusion, whereas in our study over 40% of participants were ART-naïve. Nevertheless, participants who were HBeAg-positive and HBeAg-negative appeared to represent two distinct sub-groups, consistent with the epidemiology of HIV/HBV coinfection in Europe. These differences were not limited to demographical characteristics: whereas in participants who were HBeAg-negative, 40% were HBcrAg-negative and >60% had undetectable HBV RNA levels at baseline, >95% of those who were HBeAg-positive had quantifiable HBcrAg and HBV RNA levels. The differences remained after the initiation of tenofovir therapy: whereas the majority of those who were HBeAg-negative reached undetectable HBcrAg and HBV RNA levels even in the absence of HBsAg loss, almost 50% of those who were HBeAg-positive with HBsAg loss remained HBcrAg-positive. Longitudinal data including liver biopsies would be needed to show whether these differences truly reflect a persistent distinction regarding the size and transcriptional activity of the intrahepatic cccDNA pool between individuals who were HBeAg-positive and those who were HBeAg-negative as shown in a previous cross-sectional study of mainly untreated individuals with HBV monoinfection.^32^

Interestingly, we observed an association between HBsAg seroreversion and detectable HBV RNA levels, but not with positive HBcrAg levels. Although our study was neither designed nor powered to investigate risk factors for HBV seroreversion, our findings are in line with recent studies in which detectable HBV RNA and higher HBcrAg levels were associated with biochemical relapse or hepatic flares after cessation of HBV therapy.[33], [34], [35]

In contrast to data from a large meta-analysis of over 40,000 persons with HBV monoinfection, we did not observe a significant difference between persons with HIV/HBV who were HBeAg-negative and HBeAg-positive with regard to the proportion with HBsAg loss.^7^ However, qHBsAg levels were only associated with HBsAg loss within 2 years for people who were HBeAg-negative, with 20 times higher odds of clearing HBsAg when qHBsAg was ≤1,000 IU/ml, but not in participants who were HBeAg-positive. However, lower HBV RNA and HBcrAg levels together with lack of prior ART were associated with this outcome. HBV RNA appeared to be predictive only in participants who were HBeAg-positive whereas HBcrAg was only useful in predicting HBsAg loss within 2 years in those who were HBeAg-negative. The clinical use of HBV RNA levels in individuals who were HBeAg-negative is restricted by the large proportion with undetectable or unquantifiable levels at baseline, whereas the predictive potential of HBcrAg may be flawed by its nature as a composite marker which includes HBeAg.

Our study provides robust estimates on the probability of HBsAg loss and its predictors in a large population of well-characterized persons with HIV/HBV across Europe. The availability of serially stored plasma samples allowed us to investigate the trajectories of the two novel biomarkers HBcrAg and HBV RNA, along with traditional markers, in approximately 600 persons with HIV/HBV. However, given the lack of a molecular standardization across investigational and commercial assays for HBV RNA, our results may not be directly comparable with those from other cohorts using different assays.^22^^,^^36^^,^^37^ Recently, calibration studies for a molecular standard involved in HBV RNA assays have been conducted, which may simplify comparisons in the near future.^38^ The benefit of HBcrAg is currently limited by the high LLOD of 1,000 U/ml, although this disadvantage may diminish with novel assays aimed at improving analytical sensitivity.^12^^,^^39^ As we only had data on the most likely route of HIV acquisition available, we could not reliably determine the chronology of HIV and HBV acquisition. Although we did not systematically assess HBV genotype, our sample is likely representative of the epidemiology in Europe, with genotypes A and D being most prevalent.^40^

In conclusion, our findings point towards high proportions of HBsAg loss among persons with HIV/HBV, with low baseline qHBsAg values being the strongest predictor of HBsAg loss in individuals who are HBeAg-negative and also in those who are HBeAg-positive. Additionally, HBV RNA may be a useful predictor of HBsAg loss in participants who are HBeAg-positive, whereas HBcrAg appears to be associated with HBsAg loss in individuals who are HBeAg-negative only during the first years of tenofovir therapy. Participants who were HBeAg-negative were more likely to achieve negative HBV RNA and HBcrAg levels despite similar rates of HBsAg loss, emphasizing the distinction between people who were HBeAg-negative and HBeAg-positive with HIV/HBV when considering treatment and the development of new treatment strategies.

## Abbreviations

ALT, alanine aminotransferase; ART, antiretroviral therapy; cccDNA, covalently closed circular DNA; cp/ml, copies per milliliter; HCC, hepatocellular carcinoma; LLOD, lower limit of detection; OR, odds ratio; PWH, persons with HIV; qHBsAg, quantitative hepatitis B surface antigen; SHCS, Swiss HIV Cohort Study; TAF, tenofovir alafenamide; TDF, tenofovir disoproxil fumarate.

## Authors' contributions

Study design: LB, AB, FZ, AR, GW. Performed the serological and virological analyses: MLP, BT, FSR, CS. Analyzed the data and prepared the first draft manuscript: LB. Collected and provided data for the study, contributed to the interpretation of the analyses, reviewed and commented on the draft, and approved the final version: all authors.

## Data availability

Data are available upon reasonable request. The individual level datasets generated or analyzed during the current study do not fulfill the requirements for open data access as the data is too dense and comprehensive to preserve patient privacy in persons living with HIV.

## Financial support

This work was supported by an investigator-initiated trial grant from 10.13039/100005564Gilead Sciences, United States (CO-SW-985-5602), by the NEAT-ID Foundation, by the Department of Teaching and Research, 10.13039/100018234Inselspital, Bern University Hospital, Switzerland and by the Liquid Biobank 10.13039/100018234Inselspital Bern, Switzerland. This work was performed within the framework of the IHU EVEREST (ANR-23-IAHU-0008), within the program ‘Investissements d’Avenir’ operated by the 10.13039/501100001665French National Research Agency (ANR), and by a public grant overseen by French ANR as part of the second ‘Investissements d’Avenir’ programme (ANR-17-RHUS-0003). This study has been financed within the framework of the Swiss HIV Cohort Study, supported by the 10.13039/100000001Swiss National Science Foundation (grant #33FI-0_229621), by SHCS project #809 and #868, and by the SHCS research foundation. EuroSIDA has received funding from 10.13039/100010877ViiV Healthcare 10.13039/100023970LLC, United States 10.13039/100017183Janssen Scientific Affairs, United States, 10.13039/100005565Janssen R&D, Belgium, 10.13039/100002491Bristol-Myers Squibb Company, United States, Merck Sharp & Dohme Corp, United States, 10.13039/100005564Gilead Sciences, United States, and the European Union’s Seventh Framework Programme for research, technological development, and demonstration under EuroCoord grant agreement no. 260694. The participation of centers from Switzerland has been supported by the 10.13039/100000001Swiss National Science Foundation (grant 148522).The study is also supported by a grant ([grant 10.13039/501100001732DNRF126) from the 10.13039/501100001732Danish National Research Foundation and by the International Cohort Consortium of Infectious Disease (RESPOND). The SHCS biobank was supported by the Liquid Biobank 10.13039/100018234Inselspital Bern, Switzerland, and by the Department of Teaching and Research, 10.13039/100018234Inselspital, Bern University Hospital, Switzerland. The French HIV-HBV and Biliver cohorts have been funded by the 10.13039/501100003323ANRS, France and 10.13039/100009060Sidaction, France, as well as through an unrestricted grant from 10.13039/100005564Gilead, United States. LB was supported by the «Young Talents in Clinical Research» program of the 10.13039/501100000691Swiss Academy of Medical Sciences and G. and J. Bangerter-Rhyner Foundation, Switzerland (grant YTCR 13/19). 10.13039/100007108GW was supported by a Professorship from the 10.13039/100000001Swiss National Science Foundation [PP00P3_211025]. FZ and ML received public grants overseen by the 10.13039/501100001665French National Research Agency (ANR) as part of the second ‘Investissements d'Avenir’ programme (reference: ANR-17-RHUS-0003) and by the EU (grant EU H2020-847939-IP-cure-B).

## Conflicts of interest

LB reports unrestricted research grants from Gilead Sciences and Roche Diagnostics, support for travel and conference participation from the CROI Foundation and the SAFE-ID Foundation, and speaker honoraria from Roche, all paid to his institution. AB reports receiving speaker’s fees from Gilead Sciences, Inc. MLP reports support from the French National Research Agency (ANR). BT reports research grants paid to her institution from Aligos, Assembly, BlueJay, AusperBio, and ImCheck, and lecture honoraria from Gilead Sciences France, Hospital Vall d’Hebron and the Belgian Association for the Study of the Liver, and payment for expert testimony and travel support from the International Hepatology Education Program. CB reports travel support from Gilead and participation in the post EASL HDV advisory board 2023. FSR has no conflicts of interest to declare. CS reports honoraria for educational lectures from Abbvie and expert testimony paid to her institution from Roche Diagnostics. JKR reports grants from Gilead, paid to his institution, consulting fees from Boehringer, Gilead, MSD, ViiV, speaker honoraria from Gilead, Janssen, MSD, ViiV, participation in advisory boards from BerlinCure, and being unpaid co-chair of EuroTEST. KL reports grants from MSD, and personal funding for advisory boards and educational activities as well as support for travel and conference participation from Gilead, MSD, ViiV Healthcare. LP reports stock or stock options from Novo Nordisk, Eli Lilly and Company, and Bavarian Nordic. MH reports support from Roche, patents issued, and stock or stock options from Roche as part of employee compensation. ML reports support for travel and conference participation from Gilead, Abbvie, Madrigal, speakers honoraria from Gilead, Abbvie, and support for medical writing from Roche Diagnostics and Gilead (outside of submitted work). AR reports support to his institution for advisory boards and/or travel grants from MSD, Gilead Sciences, Pfizer, and Moderna, and an investigator-initiated trial (IIT) grant from Gilead Sciences. All remuneration went to his home institution and not to AR personally, and all remuneration was provided outside the submitted work. FZ reports research grants from Aligos, Ausperbio, Bluejay, and ImCheck, consulting fees from Aligos, Ausperbio, Bluejay, GSK, nChroma, Precision, and Gilead, and speaker honoraria from Gilead. GW reports unrestricted research grants from Gilead Sciences and Roche Diagnostics, as well as advisory board fees from MSD, ViiV, and Gilead Sciences, all paid to his institution.

Please refer to the accompanying ICMJE disclosure forms for further details.
