# Supplementary material for: Circulating HBV RNA and hepatitis B core-related antigen as determinants of HBsAg loss in persons with HIV in Europe
Source: JHEP Rep. 2025 Nov 7;8(2):101671. doi: 10.1016/j.jhepr.2025.101671 (PMC12803887; doi:10.1016/j.jhepr.2025.101671)
Supplement: Multimedia component 1 [file mmc1.pdf]

Circulating HBV RNA and hepatitis B core-related antigen as determinants of HBsAg loss in persons with HIV in Europe

Lorin Begré, Anders Boyd, Marie-Laure Plissonnier, Barbara Testoni, Charles Béguelin, Franziska Suter-Riniker, Caroline Scholtès, Jürgen K. Rockstroh, Karine Lacombe, Lars Peters, Marintha Heil, Massimo Levrero, Andri Rauch, Fabien Zoulim, Gilles Wandeler, the Swiss HIV Cohort study, EuroSIDA and French HIV/HBV and Biliver cohorts

Table of contents

Fig. S1 2

Fig. S2. 3

Fig. S3 4

Fig. S4 5

Fig. S5 6

Fig. S6 7

Table S1 8

Table S2 9

Table S3. 11

Table S4. 12

Table S5 13

Table S6 14

Table S7 15

Table S8 16

Table S9 17

Supplementary Figures

Fig. S1: Inclusion flow chart.

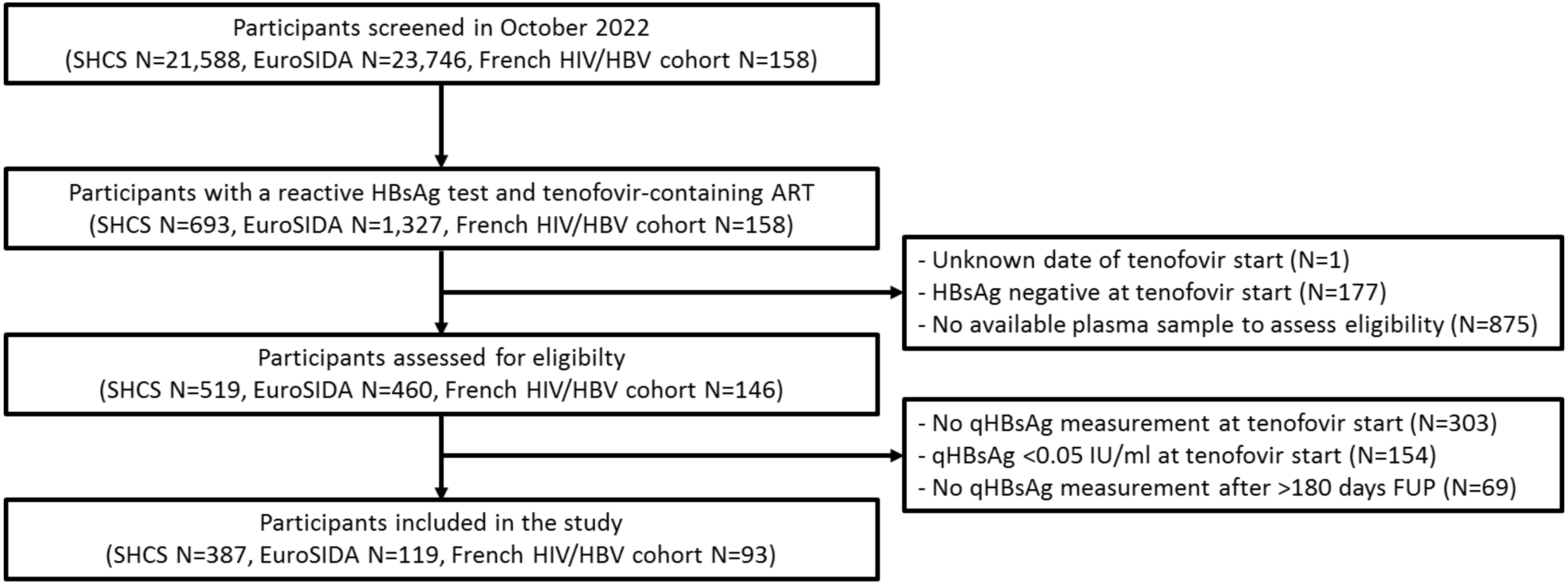

Abbreviations: ART, antiretroviral therapy; FUP, follow-up; HBsAg, hepatitis B surface antigen; HBV, hepatitis B virus; HIV, human immunodeficiency virus; qHBsAg, quantitative HBsAg; SHCS, Swiss HIV Cohort Study.

Fig. S2: qHBsAg levels in HBeAg-negative and HBeAg-positive participants at start of tenofovir therapy, after two years of follow-up and at the last follow-up visit, stratified by prior treatment with anti-HBV activity*.

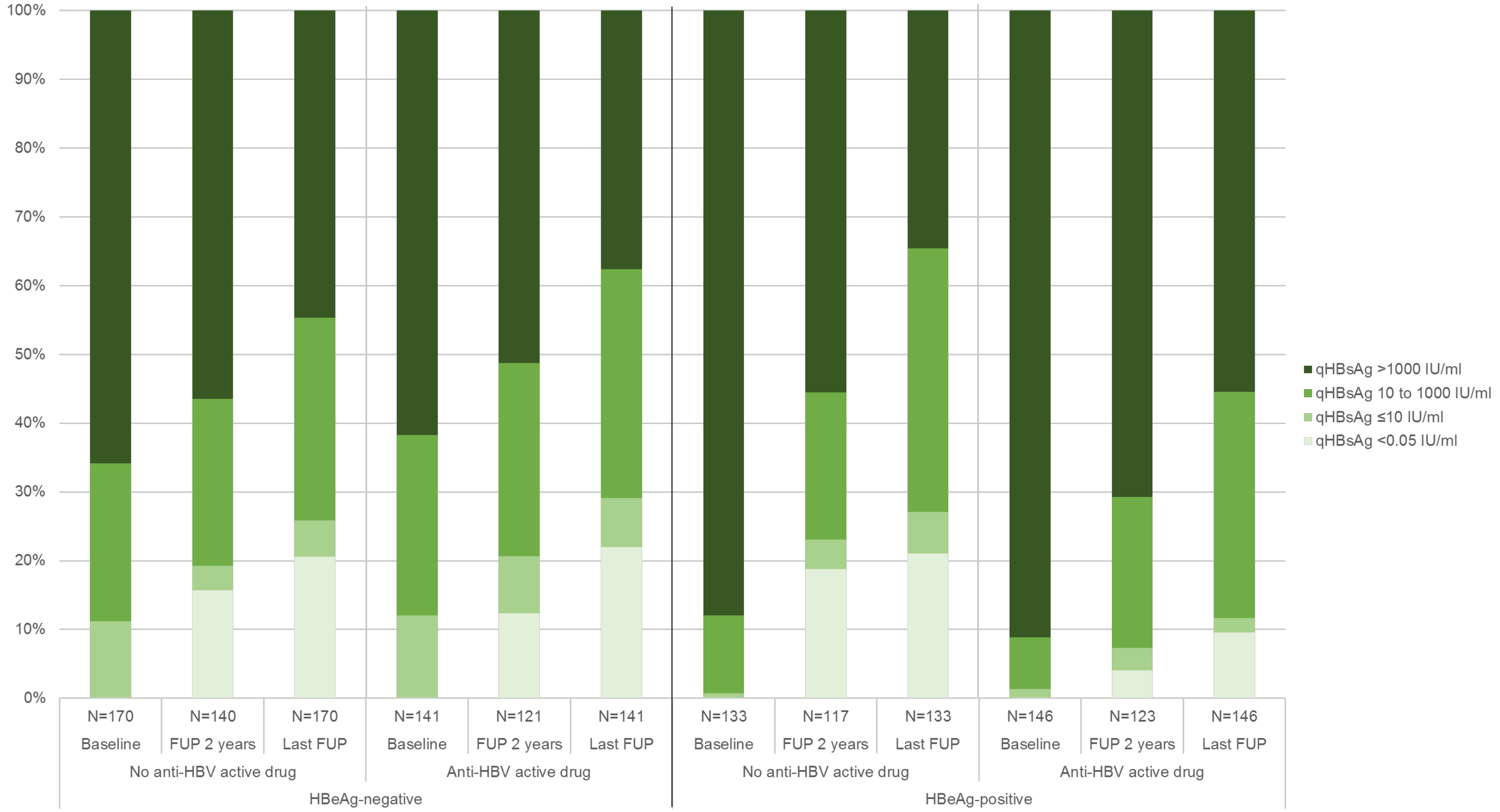

*Anti-HBV active drugs included lamivudine, emtricitabine, entecavir, adefovir and interferon-alfa. Abbreviations: ART, antiretroviral therapy; FUP, follow-up; HBeAg, IU/ml, international units per milliliter; qHBsAg, quantitative hepatitis B surface antigen.

Fig. S3: HBV DNA levels in HBeAg-negative and HBeAg-positive participants at start of tenofovir therapy, after two years of follow-up and at the last follow-up visit, stratified by prior treatment with anti-HBV activity*.

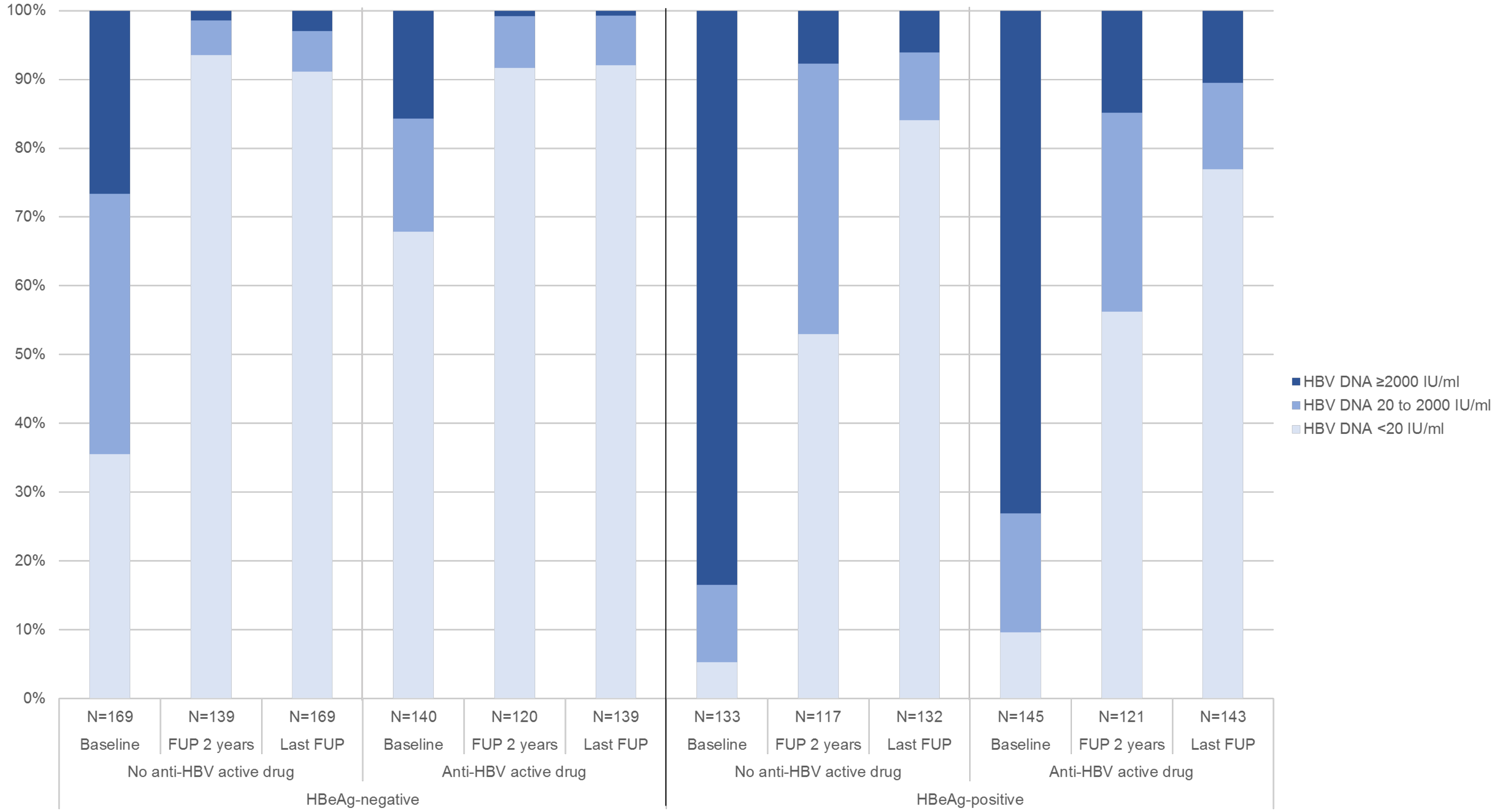

*Anti-HBV active drugs included lamivudine, emtricitabine, entecavir, adefovir and interferon-alfa. Abbreviations: ART, antiretroviral therapy; DNA, deoxyribonucleic acid; FUP, follow-up; HBeAg, hepatitis B e antigen; IU/ml, international units per milliliter; HBV, hepatitis B virus.

Fig. S4: HBcrAg levels in HBeAg-negative and HBeAg-positive participants at start of tenofovir therapy, after two years of follow-up and at the last follow-up visit, stratified by prior treatment with anti-HBV activity*.

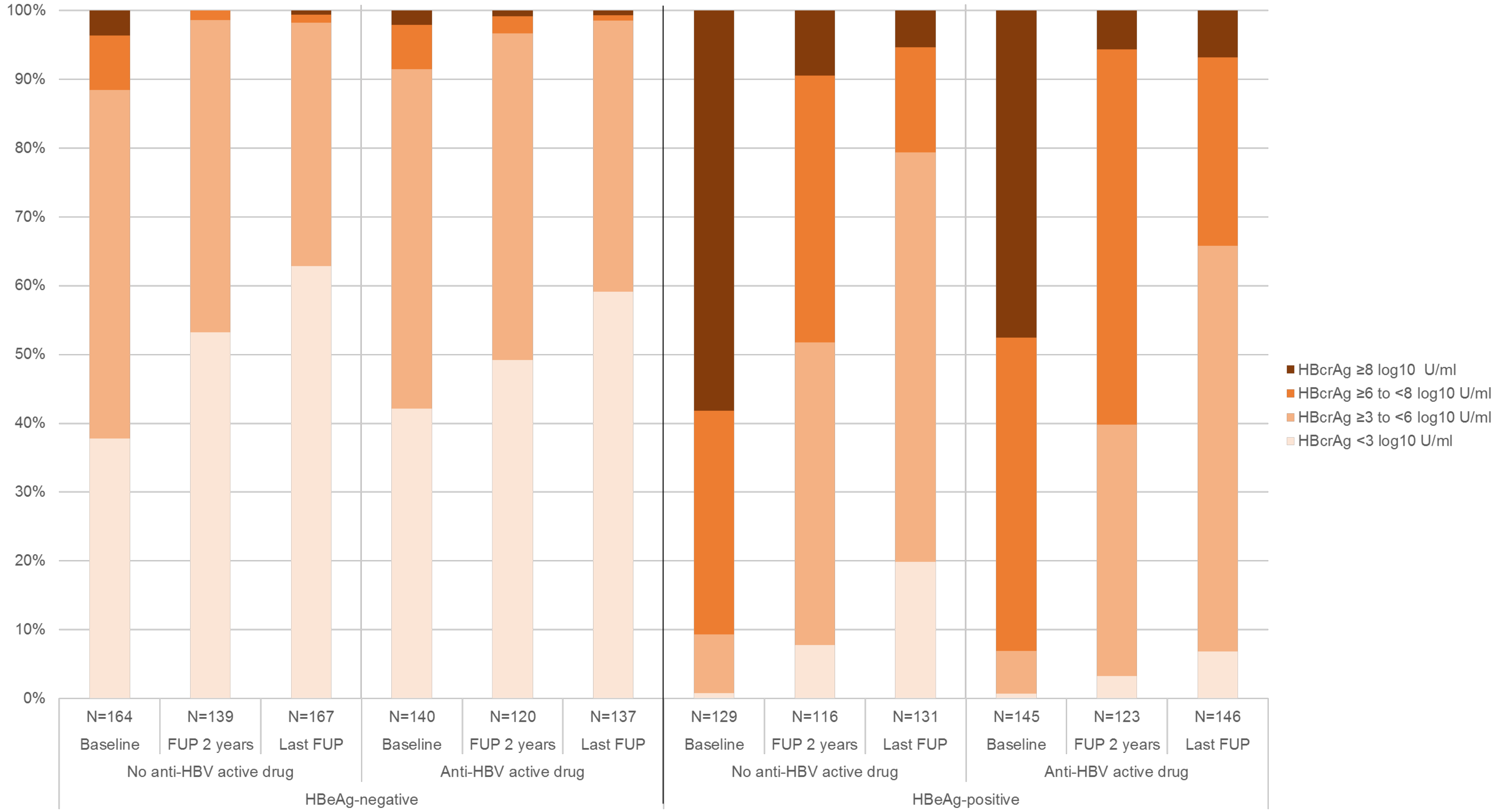

*Anti-HBV active drugs included lamivudine, emtricitabine, entecavir, adefovir and interferon-alfa. Abbreviations: ART, antiretroviral therapy; FUP, follow-up; HBcrAg, hepatitis B core-related antigen; HBeAg, hepatitis B e antigen; U/ml, units per milliliter

Fig. S5: HBV RNA levels in HBeAg-negative and HBeAg-positive participants at start of tenofovir therapy, after two years of follow-up and at the last follow-up visit, stratified by prior treatment with anti-HBV activity*.

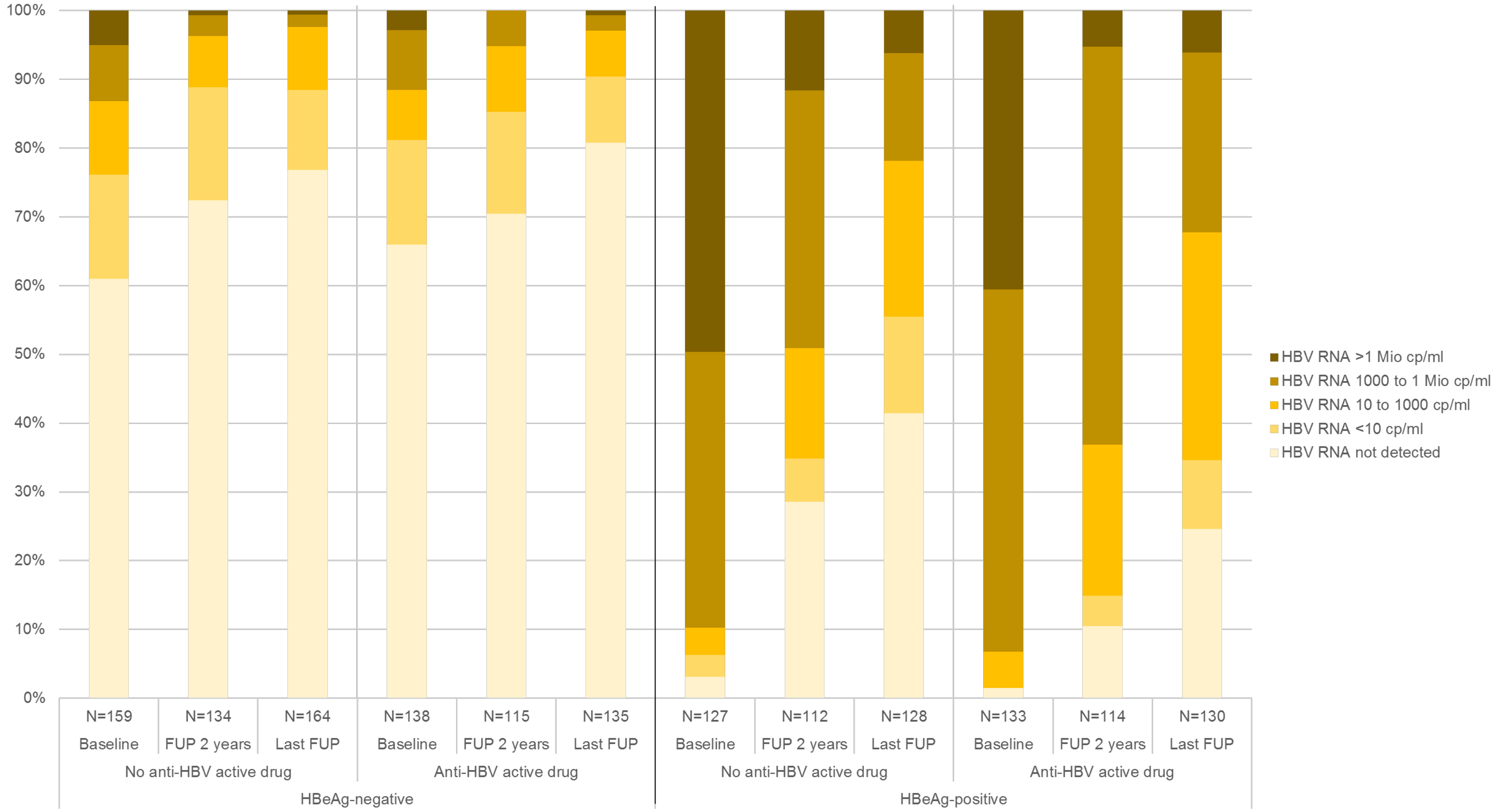

*Anti-HBV active drugs included lamivudine, emtricitabine, entecavir, adefovir and interferon-alfa. Abbreviations: ART, antiretroviral therapy; cp/ml, copies per milliliter; FUP, follow-up; HBeAg, hepatitis B e antigen Mio, million; HBV, hepatitis B virus; RNA, ribonucleic acid.

Fig. S6: Proportion of participants with HBV DNA <20 IU/ml, HBcrAg <3 log_{10} U/ml and HBV RNA below the detection limit after two years of tenofovir-containing antiretroviral therapy, stratified by qHBsAg level loss and HBeAg status.

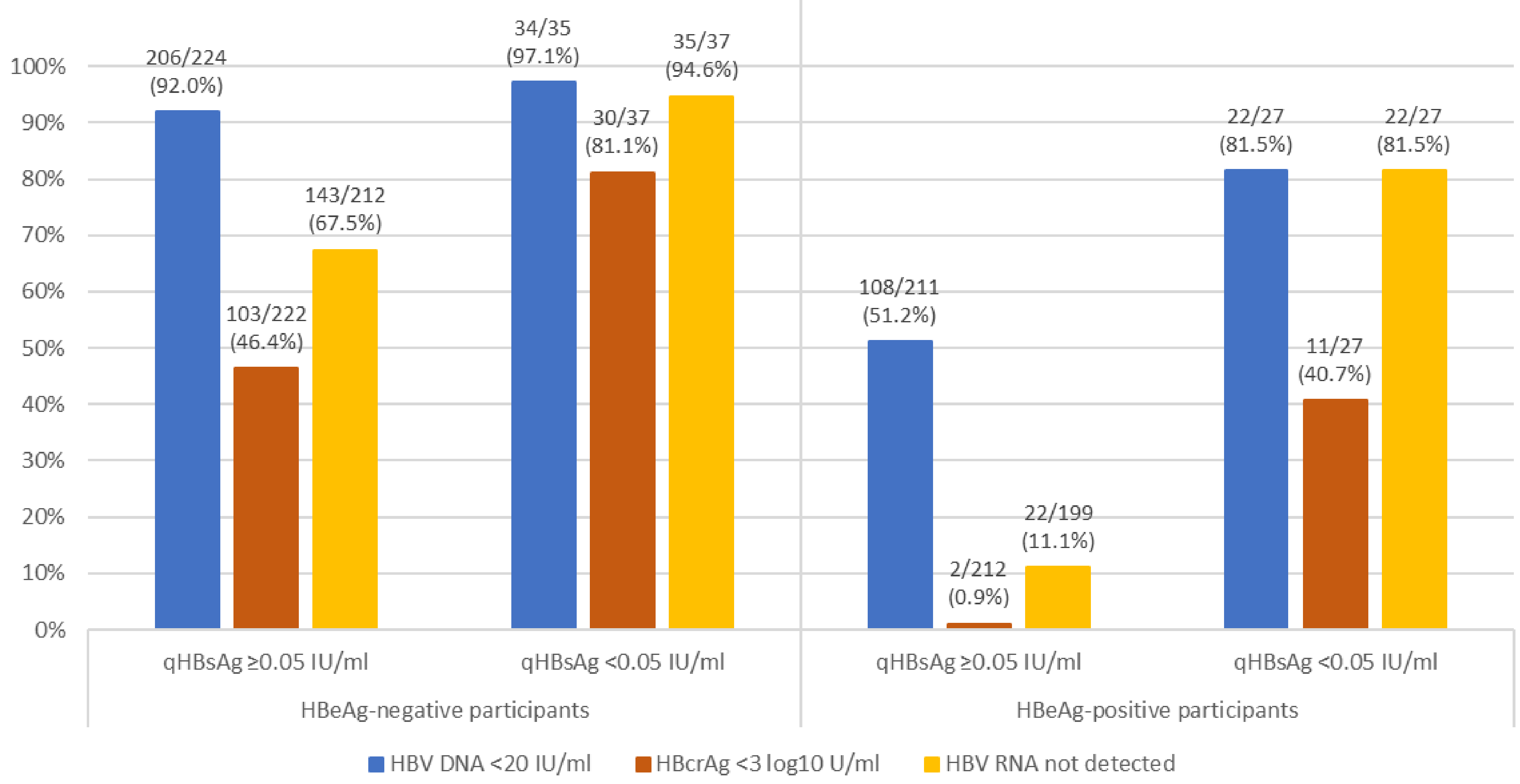

Abbreviations: DNA, deoxyribonucleic acid; HBcrAg, hepatitis B core-related antigen; HBeAg, hepatitis B e antigen; HBV, hepatitis B virus; IU/ml, international units milliliter; qHBsAg, quantitative hepatitis B surface antigen; RNA, ribonucleic acid; U/ml, units per milliliter.

Supplementary Tables

Table S1: Baseline characteristics of participants with an HBsAg-positive test before commencing tenofovir who were included and not included in the analysis.

	Excluded with reason N=526	Included in analysis N=599	P-value
Female sex at birth	92/515 (17.9%)	110/599 (18.4%)	0.83
Age, median (IQR), years	41.0 (35.0-46.0)	41.0 (35.0-47.0)	0.55
Calendar year of tenofovir start, median (IQR)	2006 (2003-2009)	2006 (2003-2009)	0.08
HBV prevalence ≥2% in country of origin*	115/511 (22.5%)	174/596 (29.2%)	**0.01**
BMI, median (IQR), kg/m^2	23.0 (20.7-25.2)	22.8 (20.9-25.3)	>0.99
On ART at start of tenofovir	280/526 (53.2%)	345/599 (57.6%)	0.14
CD4 count, median (IQR), cells/mm^3	323 (201-525)	345 (215-513)	0.41
CD4 nadir, median (IQR), cells/mm^3	161 (59-267)	170 (61-288)	0.20
HIV viral load <50 copies/ml	212/526 (40.3%)	259/599 (43.2%)	0.32
AIDS-defining condition†	151/526 (28.7%)	151/599 (25.2%)	0.19
Liver cirrhosis	31/242 (12.8%)	50/366 (13.7%)	0.76
Ever unhealthy alcohol use	40/139 (28.8%)	41/292 (14.0%)	**<0.001**

*Based on 2015 estimates from GBD 2019 Hepatitis Collaborators, Lancet Gastroenterol Hepatol 2022; 7:796-829. †According to the clinical classification of HIV disease by the US Centers for Disease Control and Prevention. Data are presented as median (IQR) for continuous measures, and n/total (%) for categorical measures. Continuous variables were compared using Wilcoxon rank-sum tests. Comparisons of categorical variables were performed using Pearson's χ^2 tests. Level of significance: $p<0.05$ (bold). Abbreviations: BMI, body mass index; HBV, hepatitis B virus; IQR, interquartile range.

Table S2: Characteristics of participants at baseline, stratified by HBeAg and ART status.

	HBeAg-negative participants			HBeAg-positive participants		
	**Not on ART**	**ART-experienced**	**P-value**	**Not on ART**	**ART-experienced**	**P-value**
	**N=141**	**N=170**		**N=106**	**N=173**	
Female sex at birth	41/141 (29.1%)	44/170 (25.9%)	0.53	11/106 (10.4%)	13/173 (7.5%)	0.41
Age, median (IQR), years	40.0 (34.0-46.0)	41.0 (36.0-49.0)	0.07	39.5 (33.0-45.0)	42.0 (37.0-47.0)	**0.03**
Calendar year of tenofovir start, median (IQR)	2009 (2006-2013)	2005 (2003-2008)	**<0.001**	2007 (2004-2010)	2003 (2002-2005)	**<0.001**
HBV prevalence ≥2% in country of origin*	60/140 (42.9%)	64/169 (37.9%)	0.37	21/106 (19.8%)	27/172 (15.7%)	0.38
Mode of HIV acquisition			0.06			0.06
Men who have sex with men	49/141 (34.8%)	68/170 (40.0%)		67/106 (63.2%)	120/173 (69.4%)	
Heterosexual contact	63/141 (44.7%)	52/170 (30.6%)		22/106 (20.8%)	25/173 (14.5%)	
Injection drug use	19/141 (13.5%)	30/170 (17.6%)		14/106 (13.2%)	13/173 (7.5%)	
Other or unknown	10/141 (7.1%)	20/170 (11.8%)		3/106 (2.8%)	15/173 (8.7%)	
BMI, median (IQR), kg/m^2	23.8 (21.4-26.8)	22.5 (20.8-25.8)	0.07	22.7 (21.1-24.3)	22.6 (20.5-24.3)	0.50
On lamivudine or emtriticabine	0/141 (0.0%)	138/170 (81.2%)	**<0.001**	0/106 (0.0%)	146/173 (84.4%)	**<0.001**
On adefovir or entecavir	1/141 (0.7%)	2/170 (1.2%)	0.67	0/106 (0.0%)	4/173 (2.3%)	0.11
On interferon-α therapy	1/141 (0.7%)	3/170 (1.8%)	0.41	0/106 (0.0%)	0/173 (0.0%)	
CD4 count, median (IQR), $cells/mm^3$	338 (184-443)	415 (271-564)	**<0.001**	288 (181-413)	404 (265-590)	**<0.001**
CD4 nadir, median (IQR), $cells/mm^3$	234 (130-351)	145 (52-241)	**<0.001**	232 (96-320)	109 (37-228)	**<0.001**
HIV viral load <50 copies/ml	31/141 (22.0%)	123/170 (72.4%)	**<0.001**	22/106 (20.8%)	120/173 (69.4%)	**<0.001**
AIDS-defining condition†	16/141 (11.3%)	44/170 (25.9%)	**0.001**	24/106 (22.6%)	66/173 (38.2%)	**0.007**

Liver cirrhosis	10/87 (11.5%)	18/109 (16.5%)	0.32	14/67 (20.9%)	8/98 (8.2%)	**0.02**
Ever unhealthy alcohol use	13/73 (17.8%)	8/78 (10.3%)	0.18	10/58 (17.2%)	9/79 (11.4%)	0.33
HBV DNA, median (IQR), $\log_{10}$ IU/ml	2.1 (0.0-3.6)	0.0 (0.0-2.1)	**<0.001**	7.0 (4.1-8.1)	6.5 (3.8-8.0)	0.32
HBV DNA <20 IU/ml	48/141 (34.0%)	107/168 (63.7%)		7/106 (6.6%)	14/172 (8.1%)	
qHBsAg, median (IQR), $\log_{10}$ IU/ml	3.5 (2.3-4.0)	3.3 (2.5-3.8)	0.06	4.5 (3.5-5.0)	4.7 (4.1-5.0)	**0.04**
qHBsAg >1000 IU/ml	91/141 (64.5%)	108/170 (63.5%)		90/106 (84.9%)	160/173 (92.5%)	
qHBsAg >10 to 1000 IU/ml	33/141 (23.4%)	43/170 (25.3%)		15/106 (14.2%)	11/173 (6.4%)	
qHBsAg ≤10 IU/ml	17/141 (12.1%)	19/170 (11.2%)		1/106 (0.9%)	2/173 (1.2%)	
HBcrAg, median (IQR), $\log_{10}$ U/ml	3.4 (2.9-4.6)	3.1 (2.9-4.3)	0.24	8.0 (7.1-8.5)	8.0 (7.2-8.5)	0.91
HBcrAg <3 $\log_{10}$ U/ml	49/136 (36.0%)	72/168 (42.9%)	0.23	1/103 (1.0%)	1/171 (0.6%)	0.72
HBV RNA, median (IQR), $\log_{10}$ copies/ml	0.0 (0.0-0.8)	0.0 (0.0-0.8)	0.49	5.9 (5.1-6.4)	5.9 (5.1-6.3)	0.71
HBV RNA <LLOD	83/134 (61.9%)	105/163 (64.4%)	0.66	4/102 (3.9%)	2/158 (1.3%)	0.16
Ever Hepatitis D antibody positive	23/124 (18.5%)	31/152 (20.4%)	0.70	7/95 (7.4%)	11/159 (6.9%)	0.89
Ever Hepatitis D RNA positive	14/122 (11.5%)	15/149 (10.1%)	0.71	4/96 (4.2%)	7/158 (4.4%)	0.92
Hepatitis C antibody positive	15/124 (12.1%)	27/159 (17.0%)	0.25	13/95 (13.7%)	10/153 (6.5%)	0.06
Hepatitis C RNA positive	10/122 (8.2%)	13/154 (8.4%)	0.94	9/91 (9.9%)	2/148 (1.4%)	**0.002**
ALT elevation ≥5x ULN	8/136 (5.9%)	6/168 (3.6%)	0.34	6/103 (5.8%)	18/170 (10.6%)	0.18

*Based on 2015 estimates from GBD 2019 Hepatitis Collaborators, Lancet Gastroenterol Hepatol 2022; 7:796-829. †According to the clinical classification of HIV disease by the US Centers for Disease Control and Prevention. Data are presented as median (IQR) for continuous measures, and n/total (%) for categorical measures. Continuous variables were compared using Wilcoxon rank-sum tests. Comparisons of categorical variables were performed using Pearson's $\chi 2$ tests. Level of significance: $p<0.05$ (bold). Abbreviations: AIDS, acquired immunodeficiency syndrome; ALT, alanine aminotransferase; BMI, body mass index; ART, antiretroviral therapy; DNA, deoxyribonucleic acid; HBcrAg, hepatitis B core-related antigen; HBeAg, hepatitis B e antigen; HBV, hepatitis B virus; IQR, interquartile range; LLOD, lower limit of detection; qHBsAg, quantitative hepatitis B surface antigen; RNA, ribonucleic acid; ULN, upper limit of normal.

Table S3: Determinants of HBsAg loss at the last follow-up visit among HBeAg-negative participants.

		Univariable analysis		Multivariable analysis			
				with HBcrAg (N=296)		with HBV RNA (N=290)	
	N	**OR (95% CI)**	**P-value**	**OR (95% CI)**	**P-value**	**OR (95% CI)**	**P-value**
qHBsAg ≤1000 IU/ml	311	5.69 (3.16-10.26)	**<0.001**	7.64 (3.78-15.44)	**<0.001**	6.82 (3.39-13.70)	**<0.001**
HBV DNA, per 1 log_{10} IU/ml increase	309	0.93 (0.81-1.06)	0.25	0.94 (0.77-1.15)	0.55	1.05 (0.85-1.29)	0.67
HBV RNA, per 1 log_{10} cp/ml increase	297	0.90 (0.76-1.08)	0.26			0.98 (0.75-1.29)	0.89
HBcrAg, per 1 log_{10} U/ml increase	304	1.01 (0.83-1.22)	0.93	1.25 (0.92-1.71)	0.16		
ALT, per 10 IU/ml increase	304	1.03 (1.00-1.06)	0.10	1.03 (0.99-1.07)	0.13	1.04 (1.00-1.08)	**0.05**
Age, per 10 years	311	1.23 (0.94-1.61)	0.12	1.16 (0.84-1.60)	0.36	1.18 (0.85-1.62)	0.32
Female sex at birth	311	0.74 (0.39-1.39)	0.35	1.29 (0.62-2.70)	0.50	1.26 (0.60-2.64)	0.54
CD4 <200 cells/ul	309	1.05 (0.53-2.09)	0.89	0.95 (0.42-2.18)	0.91	1.15 (0.49-2.68)	0.75
ART-experienced	311	0.92 (0.53-1.59)	0.76	0.75 (0.39-1.46)	0.40	0.83 (0.42-1.61)	0.58
Follow-up time, per 1 log_{10} years	311	3.03 (1.32-6.93)	**0.01**	3.13 (1.24-7.86)	**0.02**	3.07 (1.22-7.75)	**0.02**

The determinants of HBsAg loss were assessed using univariable and multivariable logistic regression models. Wald tests were performed to assess statistical significance of individual covariables with significance being defined as p<0.05 (bold). Abbreviations: ALT, alanine aminotransferase; ART, antiretroviral therapy; CI, confidence interval; cp/ml, copies per milliliter; DNA, deoxyribonucleic acid; HBcrAg, hepatitis B core-related antigen; HBeAg, hepatitis B e antigen; HBV, hepatitis B virus; IU/ml, IU/l, international units per liter; international units per milliliter; OR, odds ratio; qHBsAg, quantitative hepatitis B surface antigen; RNA, ribonucleic acid; U/ml, units per milliliter.

Table S4: Determinants of HBsAg loss at the last follow-up visit among HBeAg-positive participants.

	Univariable analysis			Multivariable analysis			
				with HBcrAg (N=268)		with HBV RNA (N=255)	
	N	**OR (95% CI)**	**P-value**	**OR (95% CI)**	**P-value**	**OR (95% CI)**	**P-value**
qHBsAg ≤1000 IU/ml	279	4.32 (1.87-9.99)	**0.001**	4.82 (1.53-15.21)	**0.007**	4.98 (1.59-15.58)	**0.006**
HBV DNA, per 1 log_{10} IU/ml increase	278	1.08 (0.94-1.23)	0.27	1.31 (1.06-1.60)	**0.01**	1.42 (1.13-1.78)	**0.003**
HBV RNA, per 1 log_{10} cp/ml increase	260	0.76 (0.63-0.90)	**0.002**			0.66 (0.50-0.88)	**0.005**
HBcrAg, per 1 log_{10} U/ml increase	274	0.77 (0.59-1.00)	0.05	0.74 (0.50-1.09)	0.13		
ALT, per 10 IU/ml increase	273	0.99 (0.97-1.02)	0.62	1.00 (0.97-1.02)	0.67	1.00 (0.97-1.02)	0.71
Age, per 10 years	279	1.15 (0.77-1.70)	0.49	1.38 (0.88-2.15)	0.16	1.44 (0.92-2.26)	0.11
Female sex at birth	279	2.03 (0.75-5.45)	0.16	2.04 (0.64-6.52)	0.23	1.94 (0.59-6.37)	0.28
CD4 <200 cells/ul	279	1.75 (0.83-3.70)	0.14	1.11 (0.45-2.74)	0.83	1.15 (0.46-2.90)	0.76
ART-experienced	279	0.35 (0.18-0.69)	**0.002**	0.39 (0.18-0.86)	**0.02**	0.45 (0.20-1.00)	0.05
Follow-up time, per 1 log_{10} years	279	3.32 (1.13-9.78)	**0.03**	3.42 (1.06-11.05	**0.04**	2.58 (0.77-8.58)	0.12

The determinants of HBsAg loss were assessed using univariable and multivariable logistic regression models. Wald tests were performed to assess statistical significance of individual covariables with significance being defined as p<0.05 (bold). Abbreviations: ALT, alanine aminotransferase; ART, antiretroviral therapy; CI, confidence interval; cp/ml, copies per milliliter; DNA, deoxyribonucleic acid; HBcrAg, hepatitis B core-related antigen; HBeAg, hepatitis B e antigen; HBV, hepatitis B virus; IU/l, international units per liter; IU/ml, international units per milliliter; OR, odds ratio; qHBsAg, quantitative hepatitis B surface antigen; RNA, ribonucleic acid; U/ml, units per milliliter.

Table S5: Determinants of HBsAg loss after two years of tenofovir-containing antiretroviral therapy among HBeAg-negative participants.

		Univariable analysis		Multivariable analysis			
				with HBcrAg (N=248)		with HBV RNA (N=242)	
	N	**OR (95% CI)**	**P-value**	**OR (95% CI)**	**P-value**	**OR (95% CI)**	**P-value**
qHBsAg ≤1000 IU/ml	261	7.29 (3.26-16.28)	**<0.001**	22.82 (6.99-74.54)	**<0.001**	19.91 (6.21-63.85)	**<0.001**
HBV DNA, per 1 $\log_{10}$ IU/ml increase	259	1.06 (0.91-1.23)	0.47	0.99 (0.76-1.28))	0.91	1.11 (0.86-1.44)	0.42
HBV RNA, per 1 $\log_{10}$ cp/ml increase	248	1.05 (0.87-1.26)	0.62			1.30 (0.93-1.84)	0.13
HBcrAg, per 1 $\log_{10}$ U/ml increase	255	1.19 (0.96-1.49)	0.12	1.79 (1.16-2.76)	**0.008**		
ALT, per 10 IU/ml increase	254	1.03 (1.00-1.06)	0.07	1.03 (0.99-1.07)	0.11	1.04 (1.01-1.08)	**0.03**
Age, per 10 years	261	1.18 (0.83-1.67)	0.35	1.19 (0.77-1.86)	0.43	1.18 (0.77-1.82)	0.45
Female sex at birth	261	1.55 (0.74-3.24)	0.24	5.37 (1.93-14.93)	**0.001**	4.68 (1.71-12.82)	**0.003**
CD4 <200 cells/ul	260	1.22 (0.52-2.87)	0.64	0.75 (0.25-2.21)	0.60	0.91 (0.31-2.65)	0.86
ART-experienced	261	0.72 (0.36-1.45)	0.36	0.75 (0.30-1.84)	0.53	0.92 (0.37-2.26)	0.85

The determinants of HBsAg loss were assessed using univariable and multivariable logistic regression models. Wald tests were performed to assess statistical significance of individual covariables with significance being defined as p<0.05 (bold). Abbreviations: ALT, alanine aminotransferase; ART, antiretroviral therapy; CI, confidence interval; cp/ml, copies per milliliter; DNA, deoxyribonucleic acid; HBcrAg, hepatitis B core-related antigen; HBeAg, hepatitis B e antigen; HBV, hepatitis B virus; IU/l, international units per liter; IU/ml, international units per milliliter; OR, odds ratio; qHBsAg, quantitative hepatitis B surface antigen; RNA, ribonucleic acid; U/ml, units per milliliter.

Table S6: Determinants of HBsAg loss after two years of tenofovir-containing antiretroviral therapy among HBeAg-positive participants.

		Univariable analysis		Multivariable analysis			
				with HBcrAg (N=230)		with HBV RNA (N=219)	
	N	OR (95% CI)	P-value	OR (95% CI)	P-value	OR (95% CI)	P-value
qHBsAg ≤1000 IU/ml	240	3.57 (1.34-9.53)	**0.01**	1.43 (0.41-5.09)	0.58	1.77 (0.50-6.25)	0.38
HBV DNA, per 1 log_{10} IU/ml increase	239	0.92 (0.79-1.07)	0.28	1.04 (0.84-1.28)	0.75	1.12 (0.88-1.41)	0.36
HBV RNA, per 1 log_{10} cp/ml increase	223	0.64 (0.53-0.79)	**<0.001**			0.64 (0.47-0.85)	**0.003**
HBcrAg, per 1 log_{10} U/ml increase	235	0.63 (0.47-0.85)	**0.003**	0.66 (0.44-1.00)	**0.05**		
ALT, per 10 IU/l increase	235	1.00 (0.98-1.03)	0.86	1.01 (0.98-1.03)	0.60	1.00 (0.98-1.03)	0.64
Age, per 10 years	240	0.94 (0.58-1.52)	0.79	1.15 (0.67-1.96)	0.61	1.22 (0.71-2.09)	0.47
Female sex at birth	240	1.88 (0.59-6.05)	0.29	2.04 (0.54-7.72)	0.30	1.88 (0.49-7.29)	0.36
CD4 <200 cells/µl	240	1.47 (0.58-3.71)	0.42	0.78 (0.26-2.36)	0.66	0.81 (0.26-2.51)	0.72
ART-experienced	240	0.22 (0.09-0.54)	**0.001**	0.22 (0.08-0.58	**0.002**	0.27 (0.10-0.74)	**0.01**

The determinants of HBsAg loss were assessed using univariable and multivariable logistic regression models. Wald tests were performed to assess statistical significance of individual covariables with significance being defined as p<0.05 (bold). Abbreviations: ALT, alanine aminotransferase; ART, antiretroviral therapy; CI, confidence interval; cp/ml, copies per milliliter; DNA, deoxyribonucleic acid; HBcrAg, hepatitis B core-related antigen; HBeAg, hepatitis B e antigen; HBV, hepatitis B virus; IU/l, international units per liter; IU/ml, international units per milliliter; OR, odds ratio; qHBsAg, quantitative hepatitis B surface antigen; RNA, ribonucleic acid; U/ml, units per milliliter.

Table S7: Determinants of HBsAg loss at the last follow-up visit: sensitivity analyses excluding participants with detectable hepatitis C RNA at baseline or ever replicating hepatitis D infection.

		**Univariable analysis**		**Multivariable analysis**			
	**N**	**OR (95% CI)**	**P-value**	**OR (95% CI)**	**P-value**	**OR (95% CI)**	**P-value**
**HBeAg-negative participants**							
				**with HBcrAg (N=252)**		**with HBV RNA (N=246)**	
qHBsAg ≤1000 IU/ml	266	6.38 (3.36-12.09)	**<0.001**	9.20 (4.29-19.74)	**<0.001**	8.48 (3.95-18.22)	**<0.001**
HBV DNA, per 1 log_{10} IU/ml increase	264	0.90 (0.78-1.04)	0.15	0.88 (0.71-1.09)	0.24	0.98 (0.79-1.23)	0.89
HBV RNA, per 1 log_{10} cp/ml increase	252	0.91 (0.75-1.09)	0.30			1.02 (0.77-1.36)	0.88
HBcrAg, per 1 log_{10} U/ml increase	259	1.02 (0.83-1.25)	0.85	1.33 (0.95-1.86)	0.10		
ALT, per 10 IU/ml increase	260	1.03 (1.00-1.07)	0.08	1.03 (0.99-1.08)	0.16	1.04 (1.00-1.09)	0.06
Age, per 10 years	266	1.25 (0.95-1.65)	0.11	1.14 (0.81-1.61)	0.45	1.18 (0.83-1.64)	0.38
Female sex at birth	266	0.77 (0.39-1.51)	0.45	1.30 (0.59-2.89)	0.52	1.28 (0.58-2.85)	0.54
CD4 <200 cells/ul	264	1.05 (0.48-2.27)	0.91	1.16 (0.43-3.10)	0.77	1.57 (0.58-4.23)	0.37
ART-experienced	266	0.97 (0.54-1.75)	0.93	0.67 (0.33-1.39)	0.29	0.75 (0.36-1.56)	0.44
Follow-up time, per 1 log_{10} years	266	2.43 (1.01-5.84)	**0.05**	2.53 (0.93-6.87)	0.07	2.55 (0.94-6.93)	0.07
**HBeAg-positive participants**							
				**with HBcrAg (N=247)**		**with HBV RNA (N=234)**	
qHBsAg ≤1000 IU/ml	258	5.05 (2.05-12.42)	**<0.001**	4.72 (1.36-16.32)	**0.01**	4.99 (1.47-16.96)	**0.01**
HBV DNA, per 1 log_{10} IU/ml increase	257	1.06 (0.92-1.22)	0.39	1.29 (1.04-1.59)	**0.02**	1.38 (1.10-1.74)	**0.006**
HBV RNA, per 1 log_{10} cp/ml increase	239	0.73 (0.60-0.88)	**0.001**			0.66 (0.49-0.89)	**0.006**
HBcrAg, per 1 log_{10} U/ml increase	253	0.73 (0.55-0.96)	**0.03**	0.73 (0.49-1.09)	0.13		
ALT, per 10 IU/ml increase	252	0.99 (0.97-1.02)	0.64	1.00 (0.97-1.02)	0.71	1.00 (0.97-1.02)	0.73
Age, per 10 years	258	1.10 (0.73-1.65)	0.65	1.28 (0.80-2.04)	0.30	1.32 (0.83-2.10)	0.24
Female sex at birth	258	2.31 (0.84-6.32)	0.10	2.23 (0.67-7.45)	0.19	2.04 (0.59-7.06)	0.26
CD4 <200 cells/ul	258	1.66 (0.76-3.59)	0.20	1.03 (0.40-2.68)	0.95	1.12 (0.42-2.95)	0.83
ART-experienced	258	0.38 (0.19-0.76)	**0.006**	0.46 (0.21-1.04)	0.06	0.54 (0.24-1.25)	0.15
Follow-up time, per 1 log_{10} years	258	3.72 (1.17-11.79)	**0.03**	3.50 (1.00-12.21)	**0.05**	2.68 (0.74-9.70)	0.13

The determinants of HBsAg loss were assessed using univariable and multivariable logistic regression models. Wald tests were performed to assess statistical significance of individual covariables with a significance being defined as p<0.05 (bold). Abbreviations: ALT, alanine aminotransferase; ART, antiretroviral therapy; CI, confidence interval; cp/ml, copies per milliliter; DNA, deoxyribonucleic acid; HBcrAg, hepatitis B core-related antigen; HBeAg, hepatitis B e antigen; HBV, hepatitis B virus; IU/ml, IU/l, international units per liter; international units per milliliter; OR, odds ratio; qHBsAg, quantitative hepatitis B surface antigen; RNA, ribonucleic acid; U/ml, units per milliliter.

Table S8: Determinants of HBsAg loss after two years: sensitivity analyses excluding participants with detectable hepatitis C RNA at baseline or ever replicating hepatitis D infection.

		Univariable analysis		Multivariable analysis			
	N	OR (95% CI)	P-value	OR (95% CI)	P-value	OR (95% CI)	P-value
**HBeAg-negative participants**							
				**with HBcrAg (N=213)**		**with HBV RNA (N=207)**	
qHBsAg ≤1000 IU/ml	225	6.02 (2.61-13.89)	**<0.001**	18.16 (5.33-61.81)	**<0.001**	16.44 (4.88-55.33)	**<0.001**
HBV DNA, per 1 log_{10} IU/ml increase	223	1.05 (0.89-1.23)	0.59	0.95 (0.72-1.25)	0.72	1.08 (0.83-1.41)	0.58
HBV RNA, per 1 log_{10} cp/ml increase	212	1.07 (0.88-1.31)	0.48			1.36 (0.95-1.95)	0.10
HBcrAg, per 1 log_{10} U/ml increase	219	1.22 (0.97-1.53)	0.09	1.87 (1.20-2.93)	**0.006**		
ALT, per 10 IU/ml increase	219	1.04 (1.00-1.08)	**0.03**	1.04 (0.99-1.08)	0.09	1.25 (1.01-1.09)	**0.02**
Age, per 10 years	225	1.18 (0.82-1.71)	0.37	1.26 (0.79-2.02)	0.33	1.25 (0.79-1.99)	0.33
Female sex at birth	225	1.72 (0.78-3.80)	0.18	6.19 (2.04-18.76)	**0.001**	5.40 (1.80-16.17)	**0.003**
CD4 <200 cells/ul	224	0.93 (0.33-2.61)	0.89	0.59 (0.16-2.19)	0.43	0.89 (0.25-3.22)	0.86
ART-experienced	225	0.97 (0.45-2.07)	0.93	0.83 (0.31-2.22)	0.71	1.01 (0.37-2.71)	0.99
**HBeAg-positive participants**							
				**with HBcrAg (N=210)**		**with HBV RNA (N=199)**	
qHBsAg ≤1000 IU/ml	220	4.02 (1.39-11.65)	**0.01**	1.31 (0.31-5.57)	0.72	1.81 (0.45-7.30)	0.41
HBV DNA, per 1 log_{10} IU/ml increase	219	0.92 (0.78-1.08)	0.31	1.05 (0.84-1.32)	0.66	1.09 (0.86-1.38)	0.48
HBV RNA, per 1 log_{10} cp/ml increase	203	0.64 (0.51-0.79)	**<0.001**			0.65 (0.48-0.89)	**0.006**
HBcrAg, per 1 log_{10} U/ml increase	215	0.60 (0.44-0.83)	**0.002**	0.62 (0.40-0.96)	**0.03**		
ALT, per 10 IU/ml increase	215	1.00 (0.98-1.03)	0.75	1.01 (0.99-1.03)	0.48	1.01 (0.99-1.03)	0.55
Age, per 10 years	220	0.92 (0.55-1.53)	0.75	1.10 (0.62-1.94)	0.75	1.15 (0.65-2.02)	0.64
Female sex at birth	220	2.25 (0.69-7.39)	0.18	2.35 (0.58-9.44)	0.23	2.03 (0.49-8.34)	0.33
CD4 <200 cells/ul	220	1.34 (0.50-3.60)	0.56	0.66 (0.19-2.21)	0.50	0.72 (0.21-.247)	0.61
ART-experienced	220	0.25 (0.10-0.62)	**0.003**	0.26 (0.09-0.70)	**0.008**	0.33 (0.12-0.92)	**0.03**

The determinants of HBsAg loss were assessed using univariable and multivariable logistic regression models. Wald tests were performed to assess statistical significance of individual covariables with significance being defined as p<0.05 (bold). Abbreviations: ALT, alanine aminotransferase; ART, antiretroviral therapy; CI, confidence interval; cp/ml, copies per milliliter; DNA, deoxyribonucleic acid; HBcrAg, hepatitis B core-related antigen; HBeAg, hepatitis B e antigen; HBV, hepatitis B virus; IU/ml, IU/l, international units per liter; international units per milliliter; OR, odds ratio; qHBsAg, quantitative hepatitis B surface antigen; RNA, ribonucleic acid; U/ml, units per milliliter.

Table S9: Characteristics of participants with qHBsAg <0.05 IU/ml after two years of tenofovir therapy, stratified by qHBsAg level at last follow-up visit.

	qHBsAg level at last follow-up		
	**qHBsAg <0.05 IU/ml N=47**	**qHBsAg ≥0.05 IU/ml N=16**	**p-value**
**Demographical and clinical characteristics at tenofovir start**			
Female sex at birth	11/47 (23.4%)	4/16 (25.0%)	0.90
Age, median (IQR), years	43.0 (34.0-51.0)	39.5 (36.5-41.5)	0.22
Calendar year of tenofovir start, median (IQR)	2008 (2004-2010)	2006 (2004-2009)	0.31
Follow-up duration, median (IQR), years	9.7 (6.5-14.0)	11.6 (8.0-15.6)	0.21
HBV prevalence ≥2% in country of origin*	11/47 (23.4%)	4/16 (25.0%)	0.90
BMI, median (IQR), kg/m^2	23.0 (20.1-24.8)	23.5 (20.2-24.9)	0.78
CD4 count, median (IQR), $cells/mm^3$	345.0 (198.0-510.0)	317.0 (242.5-432.5)	0.63
CD4 nadir, median (IQR), $cells/mm^3$	145.0 (83.0-239.0)	211.0 (74.5-274.5)	0.54
CD4/CD8 ratio, median (IQR)	0.4 (0.2-0.7)	0.3 (0.2-0.3)	0.09
HIV viral load <50 cp/ml	21/47 (44.7%)	6/16 (37.5%)	0.62
Stage C†	12/47 (25.5%)	4/16 (25.0%)	0.97
Liver cirrhosis	4/27 (14.8%)	1/10 (10.0%)	0.70
Ever unhealthy alcohol use	1/19 (5.3%)	0/7 (0.0%)	0.54
**Laboratory characteristics at tenofovir start**			
ALT, median (IQR), IU/l	44.0 (28.0-73.0)	33.0 (21.0-56.0)	0.14
ALT ≥5x ULN	5/45 (11.1%)	0/14 (0.0%)	0.19
HBV DNA, median (IQR), log_{10} IU/ml]	3.3 (0.0-5.7)	3.7 (2.1-7.4)	0.30
HBV DNA <20 IU/ml	15/47 (31.9%)	3/16 (18.8%)	0.31
qHBsAg, median (IQR), log_{10} IU/ml	1.4 (0.1-3.5)	3.7 (3.3-4.4)	**0.007**

>1000 IU/ml	17/47 (36.2%)	13/16 (81.3%)	
10-1000 IU/ml	9/47 (19.1%)	1/16 (6.3%)	
≤10 IU/ml	21/47 (44.7%)	2/16 (12.5%)	
HBcrAg, median (IQR), log_{10} U/ml	5.3 (3.0-7.5)	6.7 (3.7-7.9)	0.28
HBcrAg <3 log_{10} U/ml	11/47 (23.4%)	3/15 (20.0%)	0.78
HBV RNA, median (IQR), log_{10} copies/ml	0.8 (0.0-4.8)	4.9 (0.8-5.6)	**0.02**
HBV RNA <LLOD	23/47 (48.9%)	1/15 (6.7%)	**0.003**
HBeAg-positive	16/46 (34.8%)	10/15 (66.7%)	**0.03**
**Laboratory characteristics after 2 years of FUP**			
ALT, median (IQR), IU/l	27.0 (17.0-38.0)	21.0 (16.5-40.0)	0.65
ALT ≥5x ULN	1/47 (2.1%)	2/16 (12.5%)	0.09
HBV DNA, median (IQR), log_{10} IU/ml]	0.0 (0.0-0.0)	0.0 (0.0-1.7)	**0.03**
HBV DNA <20 IU/ml	44/45 (97.8%)	11/16 (68.8%)	**<0.001**
HBcrAg, median (IQR), log_{10} U/ml	2.9 (2.9-3.7)	2.9 (2.9-4.6)	0.33
HBcrAg <3 log_{10} U/ml	30/47 (63.8%)	9/16 (56.3%)	0.59
HBV RNA, median (IQR), log_{10} copies/ml	0.0 (0.0-0.0)	0.0 (0.0-1.3)	**<0.001**
HBV RNA <LLOD	46/47 (97.9%)	10/16 (62.5%)	**<0.001**
**Treatment-related characteristics**			
Started on TAF	1/47 (2.1%)	0/16 (0.0%)	0.56
Tenofovir interruption >30 days from baseline to 2 years FUP	4/47 (8.5%)	2/16 (12.5%)	0.64
Ever tenofovir therapy interruption >30 days	17/47 (36.2%)	6/16 (37.5%)	0.92
FUP time on tenofovir from baseline to 2 years FUP , median (IQR), %	100.0 (100.0-100.0)	100.0 (100.0-100.0)	0.96
FUP time on tenofovir from baseline to last FUP, median (IQR), %	100.0 (90.9-100.0)	100.0 (89.0-100.0)	0.91
Started with TAF	1/47 (2.1%)	0/16 (0.0%)	0.56
Switched from TDF to TAF during tenofovir	27/47 (57.4%)	9/16 (56.3%)	0.93

Lamivudine and/or emtricitabine co-therapy	46/47 (97.8%)	15/16 (93.8%)	0.42
Pegylated interferon-α co-therapy	4/47 (8.5%)	0/16 (0.0%)	0.23
Median CD4 count change from baseline to 2 years FUP, median (IQR), cells/mm^3	85.0 (4.0-227.0)	78.5 (-21.0-169.5)	0.28
Median CD4 count change from baseline to last FUP, median (IQR), cells/mm^3	299.0 (90.0-452.0)	251.5 (17.0-377.5)	0.48
Time with unsuppressed HIV viral load from baseline to 2 years FUP, median (IQR), %	13.0 (0.0-27.4)	13.1 (7.5-30.9)	0.34
Time with unsuppressed HIV viral load from baseline to last FUP, median (IQR), %	4.0 (1.1-14.6)	4.9 (2.1-7.2)	0.54
Worst reported level of adherence during first 2 years FUP			0.58
low adherence (<70%)	1/45 (2.2%)	0/14 (0.0%)	
partial adherence	1/45 (2.2%)	1/14 (7.1%)	
full adherence (>95%)	43/45 (95.6%)	13/14 (92.9%)	
Worst ever reported level of adherence during FUP			0.29
low adherence (<70%)	5/46 (10.9%)	3/14 (21.4%)	
partial adherence	5/46 (10.9%)	3/14 (21.4%)	
full adherence (>95%)	36/46 (78.3%)	8/14 (57.1%)	

*Based on 2015 estimates from GBD 2019 Hepatitis Collaborators, Lancet Gastroenterol Hepatol 2022; 7:796-829. †According to the clinical classification of HIV disease by the US Centers for Disease Control and Prevention. Data are presented as median (IQR) for continuous measures, and n/total (%) for categorical measures. Continuous variables were compared using Wilcoxon rank-sum tests. Comparisons of categorical variables were performed using Pearson's χ^2 tests. Level of significance: $p<0.05$ (bold). Abbreviations: ALT, alanine aminotransferase; BMI, body mass index; DNA, deoxyribonucleic acid; FUP, follow-up; HBcrAg, hepatitis B core-related antigen; HBeAg, hepatitis B e antigen; HBV, hepatitis B virus; IQR, interquartile range; qHBsAg, quantitative hepatitis B surface antigen; RNA, ribonucleic acid; TAF, tenofovir alafenamide; TDF, tenofovir disoproxil fumarate; ULN, upper limit of normal.
