# Supplementary material for: Circulating HBV RNA and hepatitis B core-related antigen as determinants of HBsAg loss in persons with HIV in Europe
Source: JHEP Rep. 2025 Nov 7;8(2):101671. doi: 10.1016/j.jhepr.2025.101671 (PMC12803887; doi:10.1016/j.jhepr.2025.101671)
Supplement: Multimedia component 3 [file mmc4.pdf]

JHEP Reports

Circulating HBV RNA and hepatitis B core-related antigen as determinants of HBsAg loss in persons with HIV in Europe

Authors

Lorin Begré, Anders Boyd, Marie-Laure Plissonnier, …, Andri Rauch, Fabien Zoulim, Gilles Wandeler

Correspondence

lorinaaron.begre@insel.ch (L. Begré), gilles.wandeler@insel.ch (G. Wandeler).

Graphical abstract

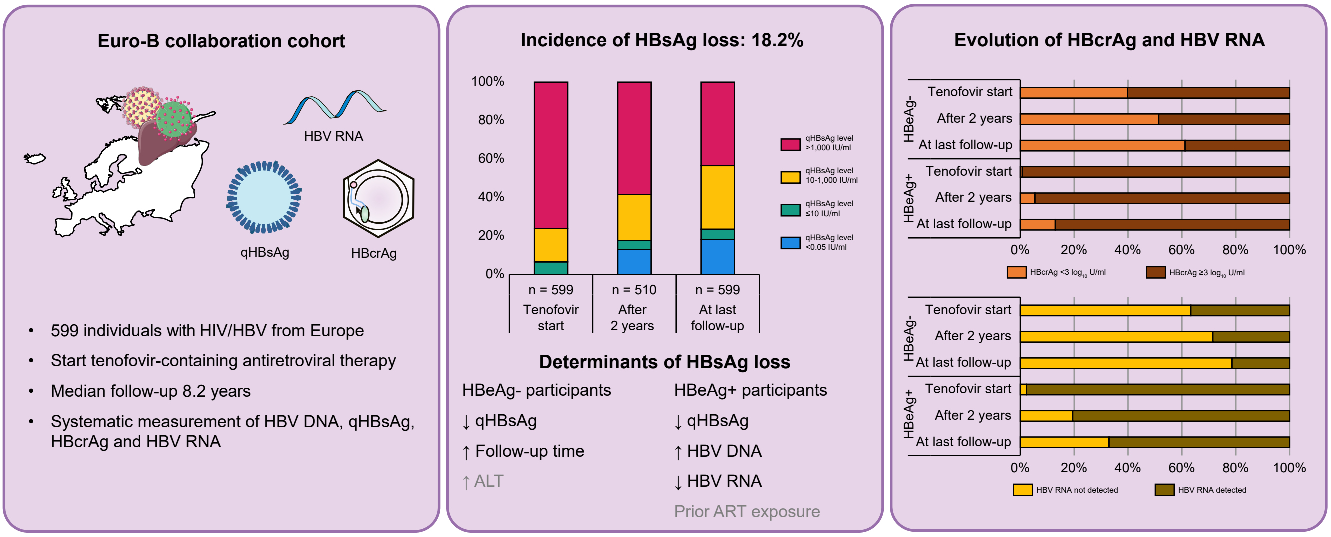

Highlights:

- Of the 599 participants, 18.2% experienced HBsAg loss within a median of 8.2 years.
- HBV RNA and HBcrAg levels decreased during therapy independent of HBsAg loss.
- Most participants who were HBeAg-negative reached negative HBV RNA and HBcrAg levels.
- Low quantitative HBsAg levels were the strongest predictor of HBsAg loss.
- In people who were HBeAg-positive, lower HBV RNA levels were also associated with HBsAg loss.

Impact and implications:

The present study builds on a multi-cohort collaboration including persons with HIV/HBV from Europe. It provides estimates on the probability of HBsAg loss during long-term tenofovir-containing antiretroviral therapy and describes the potential of the novel biomarkers HBV RNA and hepatitis B core-related antigen as its predictors. The discrepancies regarding HBV RNA and HBcrAg levels before and during therapy observed between persons who were HBeAg-negative and HBeAg-positive with HIV/HBV may influence treatment decisions and the development of new treatment strategies.

https://doi.org/10.1016/j.jhepr.2025.101671
© 2025 The Author(s). Published by Elsevier B.V. on behalf of European Association for the Study of the Liver (EASL). This is an open access article under the CC BY license (http://creativecommons.org/licenses/by/4.0/).

Circulating HBV RNA and hepatitis B core-related antigen as determinants of HBsAg loss in persons with HIV in Europe

Lorin Begré[1,2,*], Anders Boyd[3,4,5], Marie-Laure Plissonnier[6,7], Barbara Testoni[6,7], Charles Béguelin[1], Franziska Suter-Riniker[8], Caroline Scholtès[6,7,9], Jürgen K. Rockstroh[10], Karine Lacombe[11,12], Lars Peters[13], Marintha Heil[14], Massimo Levrero[6,7,15], Andri Rauch[1,†], Fabien Zoulim[6,7,15,†], Gilles Wandeler[1,16,*,†], for the Swiss HIV Cohort Study, EuroSIDA and French HIV/HBV and Biliver cohorts, Members of the EuroSIDA Study Group, Members of the French HIV/HBV and Biliver cohorts

JHEP Reports **2026**. vol. 8 | 1–11

Check for updates

Background & Aims: HBsAg loss improves clinical outcomes in persons with HIV and HBV coinfection. We aimed to evaluate if hepatitis B core-related antigen and circulating HBV RNA levels were associated with HBsAg loss in Euro-B, a multi-cohort collaboration including data from the Swiss HIV Cohort Study, EuroSIDA, and the French HIV/HBV cohort.

Methods: We included persons with HIV, a positive HBsAg, and ≥6 months of follow-up on tenofovir-containing antiretroviral therapy. We evaluated quantitative HBsAg, HBV DNA, hepatitis B core-related antigen, and HBV RNA levels over time and assessed HBsAg loss (*i.e.* quantitative HBsAg <0.05 IU/ml) during tenofovir therapy.

Results: Among 599 participants median age was 41 years (IQR 35–47), 18.4% were female and 47.3% HBeAg-positive. We observed HBsAg loss in 12.9% of participants after 2 years and in 18.2% during a median follow-up of 8.2 years (IQR 3.6–13.1). Individuals who were HBeAg-negative were more likely to have a negative hepatitis B core-related antigen and HBV RNA below the detection limit than participants who were HBeAg-positive. Quantitative HBsAg ≤1,000 IU/ml at baseline was the strongest predictor of HBsAg loss regardless of HBeAg status. Additionally, HBsAg loss was associated with lower baseline HBV RNA levels (odds ratio 0.66, 95% CI 0.49–0.88) and higher baseline HBV DNA levels in participants who were HBeAg-positive.

Conclusions: In this European cohort of persons with HIV/HBV, 18% experienced HBsAg loss during tenofovir-containing antiretroviral therapy. In addition to low baseline quantitative HBsAg levels, HBV RNA may predict HBsAg loss in individuals who are HBeAg-positive.

Clinical Trials Registration: The study is registered at ClinicalTrials.gov (NCT04984772).

© 2025 The Author(s). Published by Elsevier B.V. on behalf of European Association for the Study of the Liver (EASL). This is an open access article under the CC BY license (http://creativecommons.org/licenses/by/4.0/).

Introduction

Worldwide, ∼8% of persons with HIV (PWH) are living with chronic HBV infection and the risk of liver-related events and death is higher in this population compared with persons without HIV.[1–3] Despite the suppression of HBV viral replication in the majority of persons treated with tenofovir disoproxil fumarate (TDF) or tenofovir alafenamide (TAF) as part of antiretroviral therapy (ART), the risk of developing liver-related events, including hepatocellular carcinoma (HCC), remains elevated.[4,5] HBsAg loss improves clinical outcomes but occurs infrequently.[6,7] Several studies found higher rates of HBsAg loss in PWH compared with persons with HBV monoinfection, but the reasons for this difference remains poorly understood.[8]

Measuring intrahepatic markers of HBV activity would be ideal for assessing viral activity and would improve our understanding of the determinants of HBsAg loss. However, the invasive nature of liver biopsy reduces its widespread use. Serum quantitative HBsAg (qHBsAg) is strongly correlated with levels of replication within hepatocytes. Furthermore, low qHBsAg levels at the start of antiviral therapy were found to be associated with HBsAg loss in persons with and without HIV.[9,10] In a case-control study within the Swiss HIV Cohort Study (SHCS), we found that most persons who experienced HBsAg loss had an early decline in qHBsAg levels after starting tenofovir therapy, but individual trajectories were diverse.[11] In contrast, individuals without HBsAg loss consistently had stable levels during long-term treatment with tenofovir. Serum HBcrAg, a composite of the precore/core gene products of hepatitis B core antigen, HBeAg, and p22 core-related protein, reflects the size of the transcriptionally active covalently closed circular DNA (cccDNA) pool in the liver.[12–14] Circulating HBV RNA seems to consist mainly of pregenomic RNA and reflects

* Corresponding authors. Addresses: Department of Infectious Diseases, Inselspital, Bern University Hospital, Freiburgstrasse 20, 3010 Bern, Switzerland; Tel.: +41 31 632 25 25 (L. Begré); Department of Infectious Diseases, Inselspital, Bern University Hospital, Freiburgstrasse 20, 3010 Bern, Switzerland. Tel.: +41 31 632 25 25 (G. Wandeler).
E-mail addresses: lorinaaron.begre@insel.ch (L. Begré), gilles.wandeler@insel.ch (G. Wandeler).
† These authors contributed equally to this manuscript.
https://doi.org/10.1016/j.jhepr.2025.101671

cccDNA transcriptional activity.[15–17] HBcrAg and HBV RNA would therefore be potential candidates to help predict HBsAg loss.

We aimed to evaluate the association of HBcrAg and HBV RNA levels with HBsAg loss during treatment with tenofovir-containing ART in Euro-B, an international multi-cohort collaboration including data from persons with HIV/HBV from the SHCS, the EuroSIDA Study, and the French HIV/HBV cohort.

Patients and methods

Study design and population

We conducted a longitudinal, observational cohort study using data from the SHCS, the EuroSIDA Study, and the French HIV/HBV and Biliver cohorts.[18–20] We considered participants of these cohorts who were aged 16 years or older, had a positive HBsAg test, and commenced TDF or TAF-containing ART at some point during follow-up. To avoid double inclusion of Swiss participants, participants from EuroSIDA followed in Swiss centers were not included in the EuroSIDA participants. In this study, participants needed to have a qHBsAg ≥0.05 IU/ml at tenofovir commencement and a qHBsAg measurement after >6 months of follow-up on tenofovir. We excluded participants with unknown date of tenofovir start and with an incident HBV infection documented after the start of the first tenofovir-containing ART regimen. Participants could switch between TDF and TAF during follow-up.

We defined baseline as the start date of the first tenofovir-containing ART regimen and follow-up continued to the last available stored plasma sample, death, loss to follow-up, cessation of tenofovir, or database closure on 31 October 2022, whichever occurred first. Follow-up continued in case of interruption of tenofovir therapy when participants resumed therapy any time later on. We measured qHBsAg, HBV DNA, HBcrAg, and HBV RNA using stored plasma samples at baseline (with a window period of -12 to +6 months), 2 years after tenofovir start (with a window period of ±6 months) and at the last time point with available data. Detailed information on demographic, clinical, and laboratory data were collected according to the standardized study protocols of the respective cohorts. Local ethical committees approved the cohort studies (SHCS: Cantonal Ethics Committee Zurich, BASEC-No. 2023-02080; EuroSIDA: Regional Committees on Health Research Ethics for the Capital Region of Denmark, No. H-3-2012-049; and French HIB/HBV and Biliver cohorts: Ethics Committees of the Pitié-Salpêtrière Hospital and the Saint-Antoine Hospital in Paris, France) and written consent was obtained from all participants according to local regulations. The study is registered at ClinicalTrials.gov (NCT04984772).

Outcomes and definitions

The primary outcome was the proportion of participants with HBsAg loss, defined as a qHBsAg <0.05 IU/ml, at the last follow-up visit. Secondary outcomes were the proportion of participants with HBsAg loss at 2 years of tenofovir therapy, and the proportion with HBsAg seroreversion, defined as a qHBsAg ≥0.05 IU/ml at the last follow-up visit after having achieved a qHBsAg <0.05 IU/ml at 2 years. Other secondary outcomes included the proportion with HBV DNA suppression, negative HBcrAg and HBV RNA levels after 2 years of follow-up and at the last follow-up. HBV DNA suppression was defined as <20 IU/ml, a negative HBcrAg as <3 $\log_{10}$ U/ml, and an undetectable HBV RNA level as HBV RNA below the lower limit of detection (LLOD). Cirrhosis was assessed by liver biopsy, transient elastography >11 kPa, or APRI (aspartate aminotransferase-to-platelet ratio index) >2, as described previously.[21]

Laboratory analyses

We quantified qHBsAg using a commercial chemiluminescent microparticle immunoassay, either the Elecsys HBsAg II assays (Roche Diagnostics, Rotkreuz, Switzerland) or the ARCHITECT HBsAg (Abbott, Sligo, Ireland) with a sensitivity of ≤0.05 IU/ml. HBV DNA was measured using the commercial quantitative nucleic acid test COBAS HBV on the COBAS 4800 system (Roche Diagnostics, Rotkreuz, Switzerland) with a LLOD of 4.4 IU/ml and a linear range from 10 to 1×10^9 IU/ml. We quantified HBcrAg with the Lumipulse G HBcrAg assay on the LUMIPULSE G1200 Analyzer (Fujirebio Europe, Gent, Belgium) according to the manufacturer's instructions. As proposed by Kimura *et al.*[12], HBcrAg levels <3 $\log_{10}$ U/ml were considered negative. We determined HBV RNA levels using the COBAS HBV RNA automated investigational assay on the COBAS 6800 system (Roche Molecular Diagnostics, Pleasanton, CA, USA) with a LLOD <5 copies/ml (cp/ml) and a linear range between 10 and 10^7 cp/ml, as described previously.[22]

Statistical analysis

We compared baseline characteristics between participants who were HBeAg-positive and HBeAg-negative using Pearson's χ^2 and Wilcoxon rank-sum tests, where appropriate. We described qHBsAg, HBV DNA, HBcrAg, and HBV RNA levels at the different time points, stratified by HBeAg status. We assessed predictors of HBsAg loss after 2 years of tenofovir therapy and at the latest follow-up time point using multivariable logistic regression models, stratified by HBeAg status at baseline. Because of collinearity between HBcrAg and HBV RNA levels, we evaluated these predictors in two separate regression models. We adjusted the models for sex at birth, age, CD4 T cell count, previous ART, qHBsAg levels, HBV DNA levels, alanine aminotransferase (ALT) levels and either HBcrAg or HBV RNA levels at start of tenofovir. To adjust for different follow-up durations between participants, we included $\log_{10}$ transformed follow-up time in the logistic regression models evaluating predictors of HBsAg loss at the last follow-up time point.

Statistical significance was defined as a two-sided *p* value <0.05. We performed all analyses using Stata/MP 16.1 (StataCorp, College Station, TX, USA).

Results

Patient characteristics

Of 1,125 participants with an HBsAg-positive test before commencing tenofovir, 599 participants (53.2%) had a qHBsAg ≥0.05 IU/ml at start of tenofovir and a qHBsAg measurement after >6 months of follow-up, and hence included in the primary analysis (Fig. S1). Participants included in the analysis were more likely to have been born in a country with

an HBV prevalence ≥2% and drank less alcohol compared with those not included (Table S1). Of the included participants, 387 were from the SHCS (64.6%), 119 (19.9%) from EuroSIDA, and 93 (15.5%) from the French HIV/HBV cohort. Median age was 41 years (IQR 35–47), 110/599 (18.4%) were assigned female at birth and 279/590 (47.3%) were HBeAg-positive (Table 1). When commencing tenofovir therapy, 345/599 (57.6%) were already on ART and 289/599 (48.2%) received ≥1 anti-HBV active drug including lamivudine, emtricitabine, entecavir, adefovir, or interferon-α. Among 192 participants with available information on HBV genotype, 143 (74.3%) had genotype A infection. HBeAg-positive participants commenced tenofovir therapy earlier (median calendar year 2004, IQR 2003–2007 *vs.* 2007, IQR 2004–2010) and were more likely to be male (91.4% *vs.* 72.7%), to be born in a country with low HBV prevalence (82.7% *vs.* 59.9%), to have experienced an AIDS-defining condition (32.3% *vs.* 19.3%), and had higher ALT levels (median 54 IU/ml, IQR 37–86 *vs.* 33 IU/ml, IQR 22–57) compared with participants who were HBeAg-negative. At baseline, participants who were HBeAg-positive had higher median HBV DNA, qHBsAg, HBcrAg, and HBV RNA levels. Participants who were HBeAg-negative and participants who were HBeAg-positive and already on ART before starting tenofovir had higher CD4 counts, CD4 nadir, and were less likely to have experienced an AIDS-defining condition (Table S2). Compared with those not on ART, participants who were HBeAg-negative on ART had lower HBV DNA levels before starting tenofovir, whereas individuals who were HBeAg-positive with previous ART had higher qHBsAg levels, but similar HBV DNA levels.

HBsAg loss after 2 years and at the last follow-up visit

At baseline, 456 (76.1%) participants had qHBsAg >1,000 IU/ml. As shown in Fig. 1, this proportion declined to 298/510 (58.4%) after 2 years and 260 (43.4%) at the last follow-up visit after a median duration of 8.2 years (IQR 3.6–13.1). After 2 years of treatment, 66/510 (12.9%) participants had a qHBsAg <0.05 IU/ml, and 109/599 (18.2%) had experienced HBsAg loss at the last available follow-up visit. During follow-up, 62 (10.4%) participants died, 77 (12.9%) discontinued tenofovir therapy permanently, and 81 (13.5%) were lost to follow-up, no longer seeking HIV care at a participating study center or

Table 1. Characteristics of participants at baseline, stratified by HBeAg status.

	Total	HBeAg-negative	HBeAg-positive	
	N = 599	n = 311	n = 279	*p* value
Female sex at birth	110/599 (18.4)	85/311 (27.3)	24/279 (8.6)	**<0.001**
Age, median (IQR), years	41.0 (35.0–47.0)	41.0 (35.0–47.0)	41.0 (36.0–46.0)	0.68
Calendar year of tenofovir start, median (IQR)	2006 (2003–2009)	2007 (2004–2010)	2004 (2003–2007)	**<0.001**
HBV prevalence ≥2% in country of origin*	174/596 (29.2)	124/309 (40.1)	48/278 (17.3)	**<0.001**
Mode of HIV acquisition				**<0.001**
Men who have sex with men	309/599 (51.6)	117/311 (37.6)	187/279 (67.0)	
Heterosexual contact	166/599 (27.7)	115/311 (37.0)	47/279 (16.8)	
Injection drug use	76/599 (12.7)	49/311 (15.8)	27/279 (9.7)	
Other or unknown	48/599 (8.0)	30/311 (9.6)	18/279 (6.5)	
BMI, median (IQR), kg/m^2	22.8 (20.8–25.4)	23.2 (21.1–26.2)	22.6 (20.7–24.3)	**0.003**
On ART	345/599 (57.6)	170/311 (54.7)	173/279 (62.0)	0.07
On lamivudine or emtricitabine	286/599 (47.7)	138/311 (44.4)	146/279 (52.3)	0.05
On adefovir or entecavir	7/599 (1.2)	3/311 (1.0)	4/279 (1.4)	0.60
On interferon-α therapy	4/599 (0.7)	4/311 (1.3)	0/279 (0.0)	0.06
CD4 count, median (IQR), cells/mm^3	355 (230–533)	372 (237–533)	347 (222–547)	0.76
CD4 nadir, median (IQR), cells/mm^3	170 (61–290)	183 (72–290)	142 (50–291)	**0.04**
HIV viral load <50 copies/ml	300/599 (50.1)	154/311 (49.5)	142/279 (50.9)	0.74
AIDS-defining condition†	151/599 (25.2)	60/311 (19.3)	90/279 (32.3)	**<0.001**
Liver cirrhosis	50/366 (13.7)	28/196 (14.3)	22/165 (13.3)	0.79
Ever unhealthy alcohol use	41/292 (14.0)	21/151 (13.9)	19/137 (13.9)	0.99
HBV DNA, median (IQR), $\log_{10}$ IU/ml	3.2 (0.8–6.7)	1.3 (0.0–2.9)	6.6 (4.0–8.0)	**<0.001**
HBV DNA <20 IU/ml	177/596 (29.7)	155/309 (50.2)	21/278 (7.6)	
qHBsAg, median (IQR), $\log_{10}$ IU/ml	3.9 (3.1–4.6)	3.4 (2.3–3.9)	4.6 (3.9–5.0)	**<0.001**
qHBsAg >1,000 IU/ml	456/599 (76.1)	199/311 (64.0)	250/279 (89.6)	
qHBsAg >10–1,000 IU/ml	104/599 (17.4)	76/311 (24.4)	26/279 (9.3)	
qHBsAg ≤10 IU/ml	39/599 (6.5)	36/311 (11.6)	3/279 (1.1)	
HBcrAg, median (IQR), $\log_{10}$ U/ml	5.9 (3.1–8.0)	3.3 (2.9–4.3)	8.0 (7.2–8.5)	**<0.001**
HBcrAg <3 $\log_{10}$ U/ml	126/587 (21.5)	121/304 (39.8)	2/274 (0.7)	
HBV RNA, median (IQR), $\log_{10}$ copies/ml	2.8 (0.0–5.9)	0.0 (0.0–0.8)	5.9 (5.1–6.3)	**<0.001**
HBV RNA <LLOD	197/566 (34.8)	188/297 (63.3)	6/260 (2.3)	
Ever Hepatitis D antibody positive	72/539 (13.4)	54/276 (19.6)	18/254 (7.1)	**<0.001**
Ever Hepatitis D RNA positive	40/534 (7.5)	29/271 (10.7)	11/254 (4.3)	**0.006**
Hepatitis C antibody positive	65/531 (12.2)	42/283 (14.8)	23/248 (9.3)	0.05
Hepatitis C RNA positive	34/524 (6.5)	23/276 (8.3)	11/239 (4.6)	0.09
ALT elevation ≥5 × ULN	38/586 (6.5)	14/304 (4.6)	24/273 (8.8)	**0.04**

*Based on 2015 estimates from GBD 2019 Hepatitis Collaborators, Lancet Gastroenterol Hepatol 2022; 7:796–829.
†According to the clinical classification of HIV disease by the US Centers for Disease Control and Prevention. Data are presented as median (IQR) for continuous measures, and n/N (%) for categorical measures. Continuous variables were compared using Wilcoxon rank-sum tests. Comparisons of categorical variables were performed using Pearson's χ^2 tests. Level of significance: p <0.05 (bold). AIDS, acquired immunodeficiency syndrome; ALT, alanine aminotransferase; ART, antiretroviral therapy; BMI, body mass index; HBcrAg, hepatitis B core-related antigen; HBeAg, hepatitis B e antigen; HBV, hepatitis B virus; LLOD, lower limit of detection; qHBsAg, quantitative hepatitis B surface antigen; RNA, ribonucleic acid, ULN, upper limit of normal; XTC, lamivudine or emtricitabine.

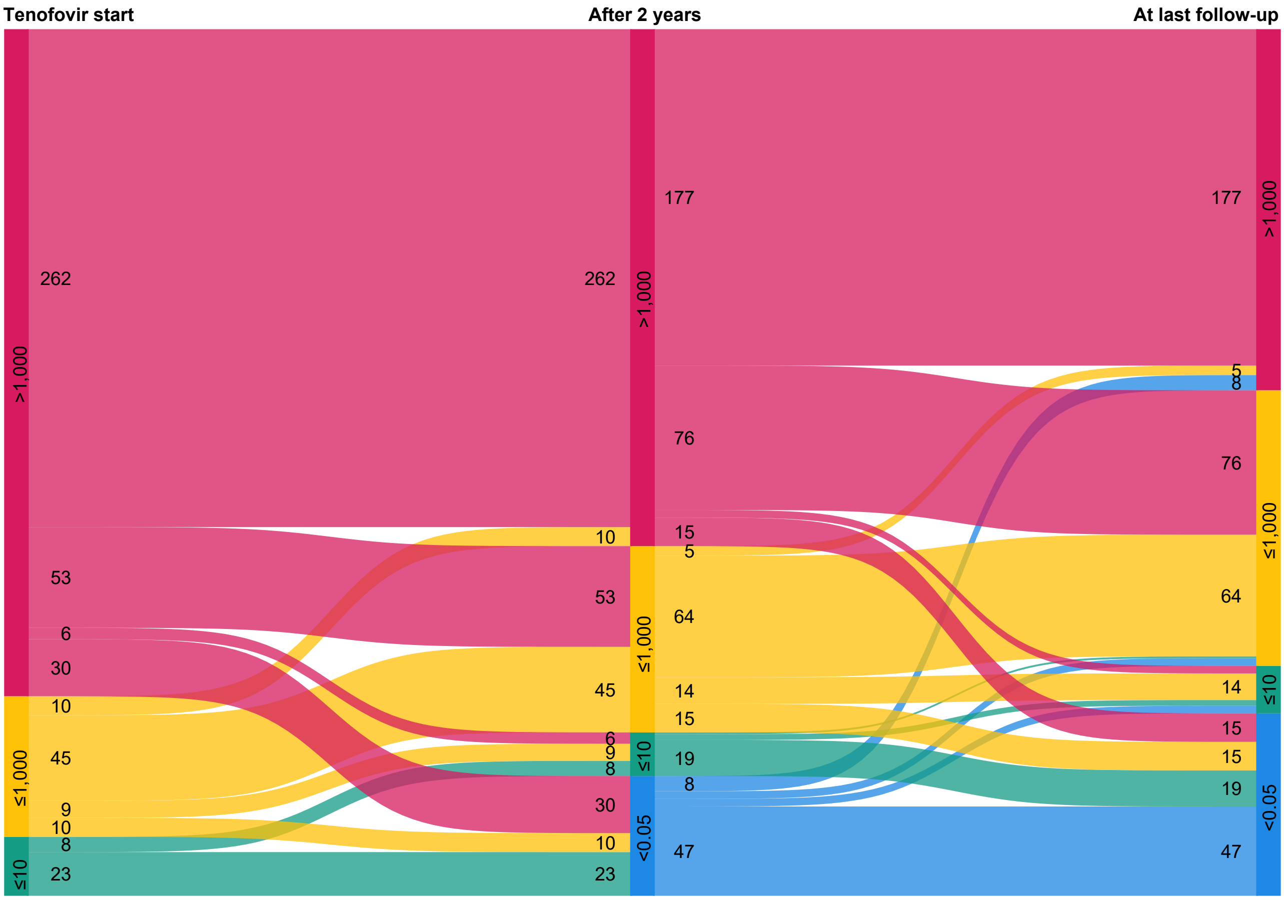

Fig. 1. Alluvial plot showing qHBsAg levels during follow-up on tenofovir-containing antiretroviral therapy among the 456 (76.1%) participants with three available measurements. Red includes participants with qHBsAg >1,000 IU/ml, yellow those with >10–1,000 IU/ml, green those with 0.05–10 IU/ml, and blue those with HBsAg loss, defined as qHBsAg <0.05 IU/ml. qHBsAg, quantitative hepatitis B surface antigen.

withdrew consent. Rates of HBsAg loss after 2 years of tenofovir therapy were similar in participants who were HBeAg-negative (37/261, 14.2%) and HBeAg-positive (27/240, 11.3%, p = 0.33), but those who were HBeAg-negative tended to be more likely to achieve HBsAg loss at the last follow-up visit (66/311, 21.2%) than those who were HBeAg-positive (42/279, 15.1%, p = 0.05).

Changes in qHBsAg, HBV DNA, HBcrAg, and HBV RNA levels during follow-up according to HBeAg status

At baseline, 112/311 (36.0%) HBeAg-negative and 29/279 (10.4%) HBeAg-positive participants had qHBsAg ≤1,000 IU/ml. During follow-up, these proportions increased to 58.5% (182/311) in HBeAg-negative and 54.5% (152/279) in HBeAg-positive participants (Fig. 2A). Participants who were HBeAg-positive with anti-HBV active therapy before tenofovir were more likely to be classified into higher qHBsAg level categories after 2 years (p = 0.003) and at the last follow-up (p = 0.001) compared with those not on ART, whereas in participants who were HBeAg-negative qHBsAg levels were similar in both subgroups after 2 years (p = 0.30) and at the last follow-up (p = 0.62, Fig. S2). HBV DNA was undetectable at baseline in 50.2% (155/309) of participants who were HBeAg-negative compared with 7.6% (21/278) of those who were HBeAg-positive (Fig. 2B). Participants who were HBeAg-negative were also more likely to be HBcrAg-negative compared with those who were HBeAg-positive from baseline (39.8% *vs.* 0.7%) until the last follow-up visit (61.2% *vs.* 13.0%, Fig. 2C), and to have HBV RNA <LLOD from baseline (63.3% *vs.* 2.3%) to the last follow-up visit (78.6 *vs.* 33.0%, Fig. 2D). In participants who were HBeAg-negative, HBV DNA, HBcrAg, and HBV RNA level categories were similar between participants with and without anti-HBV active therapy before tenofovir during follow-up (Fig. S3–S5). In contrast, participants who were HBeAg-positive on anti-HBV active therapy at baseline had higher HBcrAg (median 6.6 $\log_{10}$ U/ml, IQR 5.2–7.2) and HBV RNA (median 3.7 $\log_{10}$ cp/ml, IQR 2.1–4.9) levels after 2 years compared with those not on therapy with anti-HBV activity (HBcrAg: median 5.9 $\log_{10}$ U/ml, IQR 4.3–7.2, p = 0.03; HBV RNA: median 2.7 $\log_{10}$ cp/ml, IQR 0.0–4.9, p = 0.04). Those differences remained at the last follow-up (HBcrAg: median 5.1 $\log_{10}$ U/ml, IQR 4.1–6.5 *vs.* median 4.5 $\log_{10}$ U/ml, IQR 3.4–5.6, p <0.001; HBV RNA: median 1.8 $\log_{10}$ cp/ml, IQR 0.8–3.8 *vs.* median 0.8 $\log_{10}$ cp/ml, IQR 0.0–2.8, p = 0.004).

In participants who were HBeAg-negative with HBsAg loss, 59/65 (90.8%) were HBcrAg-negative and 63/64 (98.4%) had HBV RNA <LLOD at the last follow-up visit. In comparison,

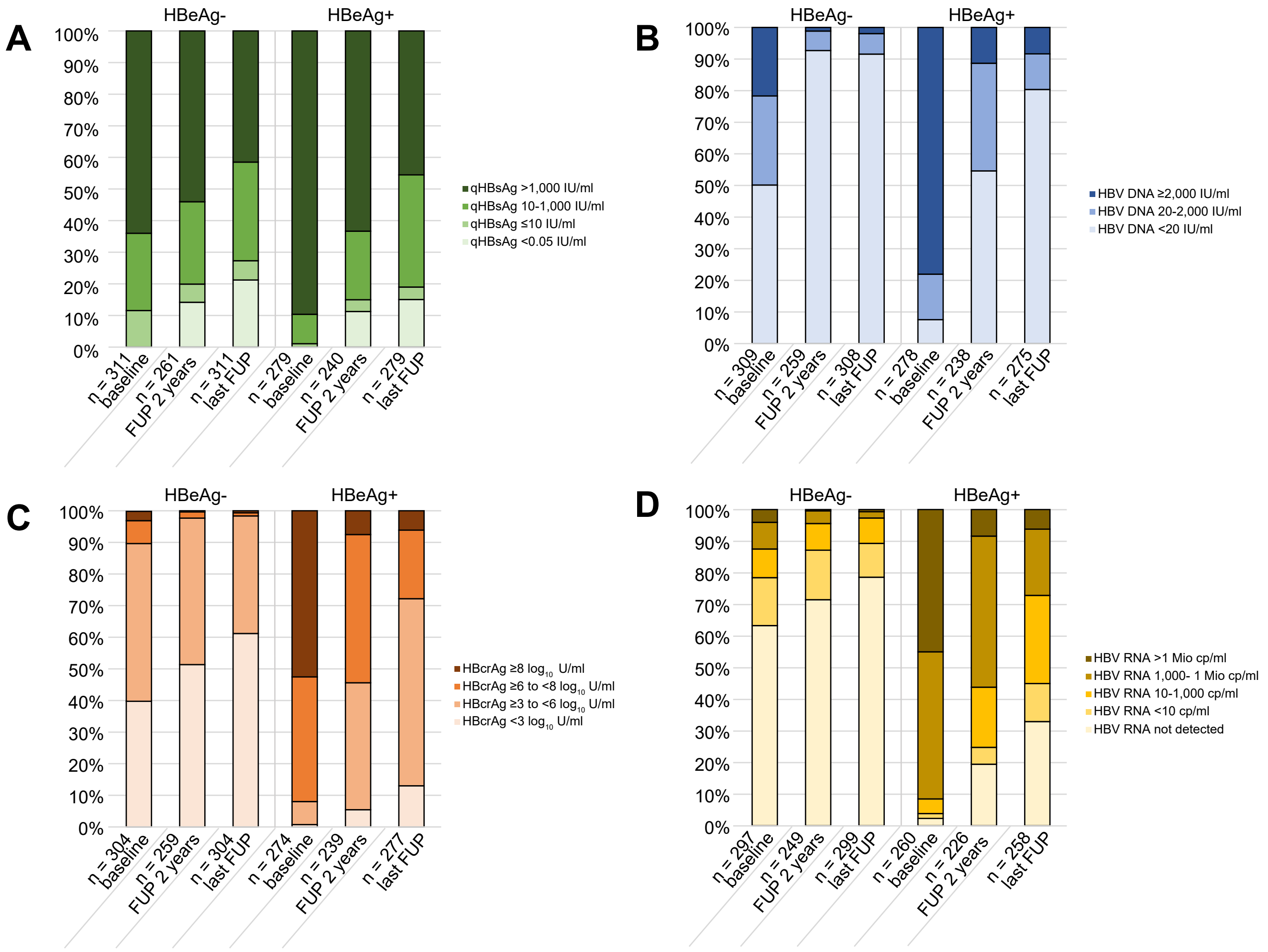

Fig 2. qHBsAg (A), HBV DNA (B), HBcrAg (C), and HBV RNA (D) levels in HBeAg-negative and HBeAg-positive participants at start of tenofovir therapy, after 2 years of follow-up and at the last follow-up visit after a median follow-up of 8.2 years (IQR 3.6-13.1). cp/ml, copies per milliliter; FUP, follow-up; HBcrAg, hepatitis B core-related antigen; HBeAg-, hepatitis B e antigen negative; HBeAg+, hepatitis B e antigen positive; Mio, million; qHBsAg, quantitative hepatitis B surface antigen; HBV, hepatitis B virus.

52.4% (22/42) of participants who were HBeAg-positive with HBsAg loss were HBcrAg-negative and 88.1% (37/42) had HBV RNA <LLOD (Fig. 3) at the last follow-up visit. At the last follow-up visit, a combination of negative HBcrAg and HBV RNA <LLOD was observed in 90.6% (58/64) of participants who were HBeAg-negative compared with 47.6% (20/42) of those who were HBeAg-positive. As shown in Fig. S6, these proportions were comparable at the 2-year follow-up time point. At 2 years, combined suppressed levels of HBcrAg and HBV RNA were observed in 75.0% (27/36) of participants who were HBeAg-negative and 33.3% (8/24) of those who were HBeAg-positive.

Predictors of HBsAg loss

In multivariable analysis, qHBsAg ≤1,000 IU/ml at baseline and follow-up duration were the strongest predictors of HBsAg loss at the last follow-up visit among participants who were HBeAg-negative (model with HBcrAg: odds ratio [OR] 7.64, 95% CI 3.78–15.44; model with HBV RNA: OR 6.82, 95% CI 3.39–13.70) and participants who were HBeAg-positive (model with HBcrAg: OR 4.82, 95% CI 1.53–15.21; model with HBV RNA: OR 4.98, 95% CI 1.59–15.58) (Fig. 4 and Tables S3 and S4). Among participants who were HBeAg-negative, neither HBcrAg, nor HBV RNA levels were associated with HBsAg loss after a median of 8.2 years of follow-up. However, higher ALT (OR 1.04, 95% CI 1.00–1.08 per 10 IU/ml increase) was associated with HBsAg loss in the multivariable model including HBV RNA but not in the one including HBcrAg. In individuals who were HBeAg-positive, lower HBV RNA (OR 0.66, 95% CI 0.50–0.88 per 1 $\log_{10}$ cp/ml increase) and higher HBV DNA levels were associated with HBsAg loss, whereas HBcrAg levels were not. Being ART-experienced was associated with HBsAg loss only in the multivariable model including HBcrAg and restricted to participants who were HBeAg-positive.

After 2 years of tenofovir therapy, HBsAg loss was associated with qHBsAg ≤1,000 IU/ml in participants who were HBeAg-negative (model with HBcrAg: OR 22.82, 95% CI 6.99–74.54; model with HBV RNA: OR 19.91, 95% CI

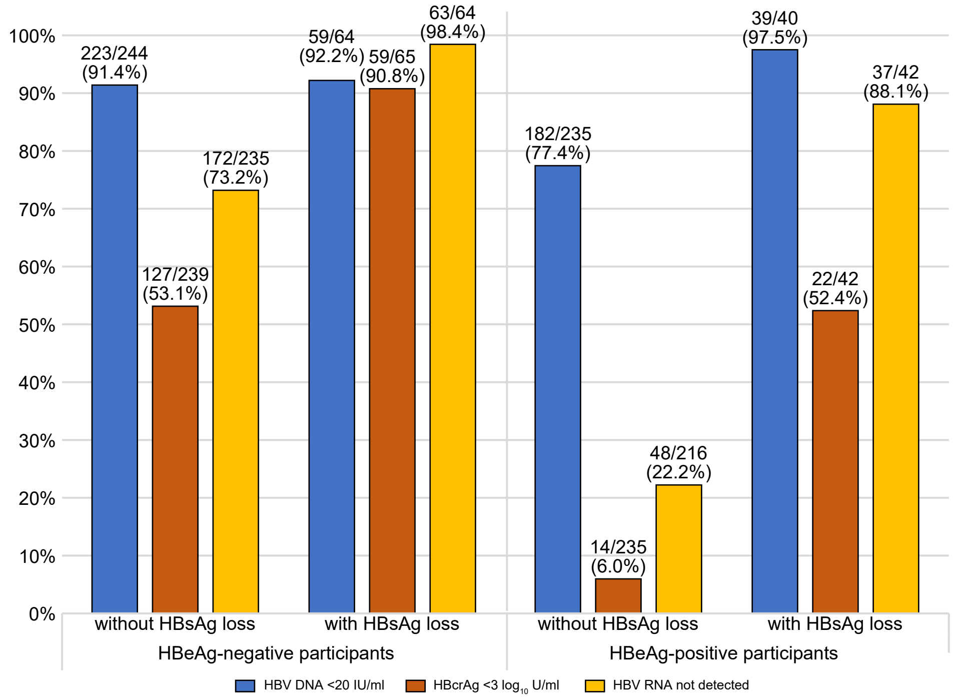

Fig. 3. Proportion of participants with HBV DNA <20 IU/ml, HBcrAg <3 $\log_{10}$ U/ml and HBV RNA below the detection limit at the last follow-up time point, stratified by HBsAg loss and HBeAg status. HBcrAg, hepatitis B core-related antigen; HBeAg, hepatitis B e antigen; HBsAg, hepatitis B surface antigen.

6.21–63.85), but not in participants who were HBeAg-positive (model with HBcrAg: OR 1.43, 95% CI 0.41–5.09; model with HBV RNA: OR 1.77, 95% CI 0.50–6.25) (Tables S5 and S6). In individuals who were HBeAg-negative, higher HBcrAg levels (OR 1.79, 95% CI 1.16–2.76 per 1 $\log_{10}$ U/ml increase) and female sex at birth were additionally associated with HBsAg loss after 2 years of therapy. Higher ALT levels were associated with HBsAg loss only in the model including HBV RNA in individuals who were HBeAg-negative. In participants who were HBeAg-positive, lower HBV RNA (OR 0.64, 95% CI 0.47–0.85 per 1 $\log_{10}$ cp/ml increase) and HBcrAg levels (OR 0.66, 95% CI 0.44–1.00 per 1 $\log_{10}$ U/ml increase) as well as prior ART was associated with HBsAg loss after 2 years.

In sensitivity analyses, we excluded all participants with detectable hepatitis C RNA at tenofovir start and participants who ever had replicating hepatitis D infection. As shown in Table S7, exclusion of the above-mentioned participants did not substantially influence our models. Similarly, our multivariable logistic regression models of predictors of HBsAg loss after 2 years of tenofovir therapy remained relatively unchanged (Table S8).

HBsAg seroreversion

Among individuals with three available qHBsAg measurements, 16/63 (25.3%) participants who had qHBsAg <0.05 IU/ml after 2 years seroreverted to being HBsAg-positive at the last follow-up visit. Participants with seroreversion had higher qHBsAg levels (3.7 $\log_{10}$ IU/ml, IQR 3.3–4.4 *vs.* 1.4 $\log_{10}$ IU/ml, IQR 0.1–3.5, p = 0.01) and HBV RNA levels at baseline (4.9 $\log_{10}$ cp/ml, IQR 0.8–5.6 *vs.* 0.8 $\log_{10}$ cp/ml, IQR 0.0–4.8), p = 0.02) and were more likely to be HBeAg-positive (10/15, 67% *vs.* 16/46, 35%, p = 0.03). In addition, they were more likely to have a detectable HBV viral load (5/16, 31% *vs.* 1/45, 2%, p = 0.001) and detectable HBV RNA levels (6/16, 37.5% *vs.* 1/47. 2%, p <0.001) after 2 years of follow-up despite having qHBsAg <0.05 IU/ml (Table S9).

Discussion

In this large prospective study including persons with HIV/HBV from three European cohorts, over 18% experienced HBsAg loss during a median of 8 years of tenofovir-containing ART. Participants with low qHBsAg levels were over five times more likely to experience HBsAg loss on tenofovir than those with higher levels. Lower HBV RNA levels in participants who were HBeAg-positive were additionally associated with HBsAg loss.

The proportion of HBsAg loss found in our study is consistent with data from the German HIV/HBV cohort, where 18% of participants experienced HBsAg loss during a median follow-up of 11 years.[23] Similar proportions have also been described in other cohorts including participants from China, Thailand, Australia, and Zambia.[24–26] These findings, however, contrast with data from persons with HBV monoinfection, where usually <1% per year experience HBsAg loss.[7,8] Previous studies have reported an association between lower CD4+ T cell counts or advanced HIV disease and increased rates of HBsAg loss, suggesting immune reconstitution as a driver of HBsAg loss.[9,27] Although we did not observe such an association in our study, participants who were ART-naïve and HBeAg-positive were four times more likely to experience HBsAg loss within 2 years of tenofovir therapy compared with their ART-experienced counterparts. Interestingly, we did not observe an association between prior ART and HBsAg loss

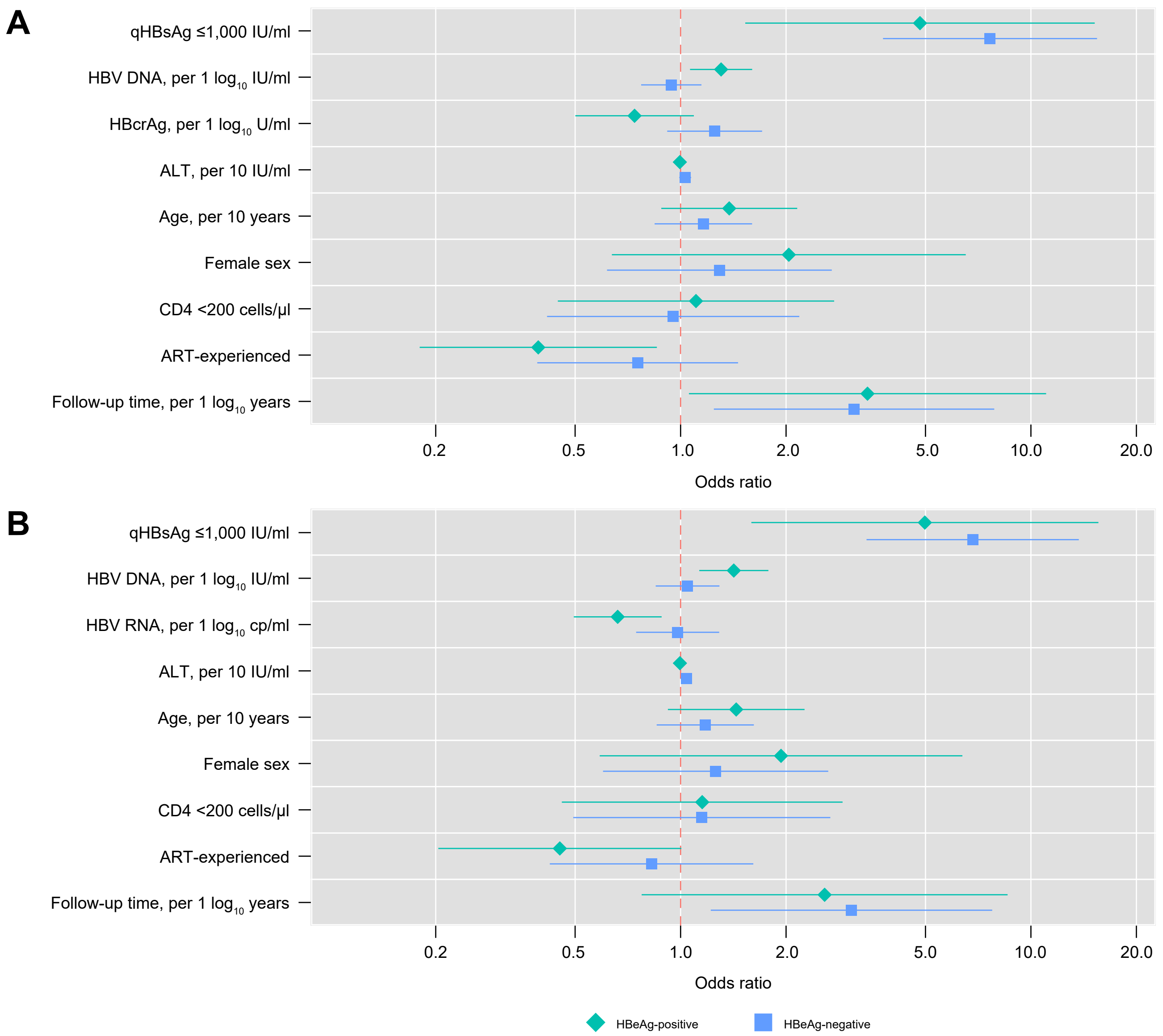

Fig. 4. Multivariable logistic regression models for HBsAg loss at the last follow-up time point. (A) with HBcrAg included in the model, (B) with HBV RNA included in the model. Except follow-up time, all parameters included in the models are baseline values. Points represent the odds ratios and bands the 95% CIs. Green refers to the model restricted to participants who were HBeAg-positive and blue to the model restricted to those who were HBeAg-negative. Wald tests were performed to assess statistical significance of individual covariables with significance defined as p <0.05. In the models including HBcrAg (panel A) the following variables were significantly associated with HBsAg loss in at least one sub-group: qHBsAg ≤1,000 IU/ml (HBeAg-negative participants p <0.001; HBeAg-positive participants p = 0.007), being ART-experienced (HBeAg-negative participants p = 0.40; HBeAg-positive participants p = 0.02), and follow-up time (HBeAg-negative participants p = 0.02; HBeAg-positive participants p = 0.04). In the models including HBV RNA (panel B) the following variables were significantly associated with HBsAg loss: qHBsAg ≤1,000 IU/ml (participants who were HBeAg-negative p <0.001; participants who were HBeAg-positive p = 0.006), HBV DNA (participants who were HBeAg-negative p = 0.67; participants who were HBeAg-positive p = 0.003), HBV RNA (participants who were HBeAg-negative p = 0.89; participants who were HBeAg-positive p = 0.005), ALT (participants who were HBeAg-negative p = 0.048; participants who were HBeAg-positive p = 0.71), and follow-up time (participants who were HBeAg-negative p = 0.02; participants who were HBeAg-positive p = 0.12). Actual odds ratios, 95% CIs and p values for all variables are depicted in Tables S3 and S4. ALT, alanine aminotransferase; ART, antiretroviral therapy; cp/ml, copies per milliliter; HBcrAg, hepatitis B core-related antigen; HBeAg, hepatitis B e antigen; qHBsAg, quantitative hepatitis B surface antigen.

among HBeAg-negative participants. As partial recovery of the impaired HBV-specific T cell response in chronic HBV infection can be observed in persons without HIV, HBeAg-positive PWH – who probably acquired HBV relatively recently, compared with their HBeAg-negative counterparts – may be able to establish robust HBV-specific T cell responses and achieve immunological control of HBV with immune reconstitution after ART uptake.[28,29]

In line with our pilot study, which relied on a median number of 12 follow-up measurements, HBV RNA and HBcrAg levels decreased in participants with and without HBsAg loss during long-term tenofovir-containing ART.[30] These findings contrast with a study including 95 persons with HIV/HBV from North America, where declining HBV RNA and HBcrAg levels were only observed in individuals who were HBeAg-positive.[31] However, in that study almost all participants were on

nucleos(t)ide analogue therapy at study inclusion, whereas in our study over 40% of participants were ART-naïve. Nevertheless, participants who were HBeAg-positive and HBeAg-negative appeared to represent two distinct sub-groups, consistent with the epidemiology of HIV/HBV coinfection in Europe. These differences were not limited to demographical characteristics: whereas in participants who were HBeAg-negative, 40% were HBcrAg-negative and >60% had undetectable HBV RNA levels at baseline, >95% of those who were HBeAg-positive had quantifiable HBcrAg and HBV RNA levels. The differences remained after the initiation of tenofovir therapy: whereas the majority of those who were HBeAg-negative reached undetectable HBcrAg and HBV RNA levels even in the absence of HBsAg loss, almost 50% of those who were HBeAg-positive with HBsAg loss remained HBcrAg-positive. Longitudinal data including liver biopsies would be needed to show whether these differences truly reflect a persistent distinction regarding the size and transcriptional activity of the intrahepatic cccDNA pool between individuals who were HBeAg-positive and those who were HBeAg-negative as shown in a previous cross-sectional study of mainly untreated individuals with HBV monoinfection.[32]

Interestingly, we observed an association between HBsAg seroreversion and detectable HBV RNA levels, but not with positive HBcrAg levels. Although our study was neither designed nor powered to investigate risk factors for HBV seroreversion, our findings are in line with recent studies in which detectable HBV RNA and higher HBcrAg levels were associated with biochemical relapse or hepatic flares after cessation of HBV therapy.[33–35]

In contrast to data from a large meta-analysis of over 40,000 persons with HBV monoinfection, we did not observe a significant difference between persons with HIV/HBV who were HBeAg-negative and HBeAg-positive with regard to the proportion with HBsAg loss.[7] However, qHBsAg levels were only associated with HBsAg loss within 2 years for people who were HBeAg-negative, with 20 times higher odds of clearing HBsAg when qHBsAg was ≤1,000 IU/ml, but not in participants who were HBeAg-positive. However, lower HBV RNA and HBcrAg levels together with lack of prior ART were associated with this outcome. HBV RNA appeared to be predictive only in participants who were HBeAg-positive whereas HBcrAg was only useful in predicting HBsAg loss within 2 years in those who were HBeAg-negative. The clinical use of HBV RNA levels in individuals who were HBeAg-negative is restricted by the large proportion with undetectable or unquantifiable levels at baseline, whereas the predictive potential of HBcrAg may be flawed by its nature as a composite marker which includes HBeAg.

Our study provides robust estimates on the probability of HBsAg loss and its predictors in a large population of well-characterized persons with HIV/HBV across Europe. The availability of serially stored plasma samples allowed us to investigate the trajectories of the two novel biomarkers HBcrAg and HBV RNA, along with traditional markers, in approximately 600 persons with HIV/HBV. However, given the lack of a molecular standardization across investigational and commercial assays for HBV RNA, our results may not be directly comparable with those from other cohorts using different assays.[22,36,37] Recently, calibration studies for a molecular standard involved in HBV RNA assays have been conducted, which may simplify comparisons in the near future.[38] The benefit of HBcrAg is currently limited by the high LLOD of 1,000 U/ml, although this disadvantage may diminish with novel assays aimed at improving analytical sensitivity.[12,39] As we only had data on the most likely route of HIV acquisition available, we could not reliably determine the chronology of HIV and HBV acquisition. Although we did not systematically assess HBV genotype, our sample is likely representative of the epidemiology in Europe, with genotypes A and D being most prevalent.[40]

In conclusion, our findings point towards high proportions of HBsAg loss among persons with HIV/HBV, with low baseline qHBsAg values being the strongest predictor of HBsAg loss in individuals who are HBeAg-negative and also in those who are HBeAg-positive. Additionally, HBV RNA may be a useful predictor of HBsAg loss in participants who are HBeAg-positive, whereas HBcrAg appears to be associated with HBsAg loss in individuals who are HBeAg-negative only during the first years of tenofovir therapy. Participants who were HBeAg-negative were more likely to achieve negative HBV RNA and HBcrAg levels despite similar rates of HBsAg loss, emphasizing the distinction between people who were HBeAg-negative and HBeAg-positive with HIV/HBV when considering treatment and the development of new treatment strategies.

Affiliations

[1]*Department of Infectious Diseases, Inselspital, Bern University Hospital, University of Bern, Bern, Switzerland;* [2]*Graduate School for Health Sciences, University of Bern, Bern, Switzerland;* [3]*Stichting HIV Monitoring, Amsterdam, The Netherlands;* [4]*Department of Infectious Diseases, Amsterdam UMC Location University of Amsterdam, Amsterdam, The Netherlands;* [5]*Department of Infectious Diseases, Amsterdam Institute for Infection and Immunity, Amsterdam, The Netherlands;* [6]*UMR PaThLiv U1350, Inserm, Université Claude Bernard Lyon 1, Lyon, France;* [7]*Lyon Hepatology Institute, IHU EVEREST, Lyon, France;* [8]*Institute for Infectious Diseases, University of Bern, Bern, Switzerland;* [9]*Department of Virology, Hospices Civils de Lyon, Lyon, France;* [10]*HIV-Clinic, Department of Medicine I, University Hospital Bonn, Bonn, Germany;* [11]*Sorbonne Université, Inserm, Institut Pierre Louis d'Épidémiologie et de Santé Publique, IPLESP, Paris, France;* [12]*AP-HP, GH Sorbonne.Université, Hôpital Saint-Antoine, Service de maladies infectieuses et tropicales, Paris, France;* [13]*CHIP, Centre of Excellence for Health, Immunity and Infections, Rigshospitalet, Copenhagen, Denmark;* [14]*Roche Molecular Diagnostics, Pleasanton, CA, USA;* [15]*Department of Hepatology, Hospices Civils de Lyon, Lyon, France;* [16]*Institute of Social and Preventive Medicine, University of Bern, Bern, Switzerland*

Abbreviations

ALT, alanine aminotransferase; ART, antiretroviral therapy; cccDNA, covalently closed circular DNA; cp/ml, copies per milliliter; HCC, hepatocellular carcinoma; LLOD, lower limit of detection; OR, odds ratio; PWH, persons with HIV; qHBsAg, quantitative hepatitis B surface antigen; SHCS, Swiss HIV Cohort Study; TAF, tenofovir alafenamide; TDF, tenofovir disoproxil fumarate.

Financial support

This work was supported by an investigator-initiated trial grant from Gilead Sciences, United States (CO-SW-985-5602), by the NEAT-ID Foundation, by the Department of Teaching and Research, Inselspital, Bern University Hospital, Switzerland and by the Liquid Biobank Inselspital Bern, Switzerland. This work was performed within the framework of the IHU EVEREST (ANR-23-IAHU-0008), within the program 'Investissements d'Avenir' operated by the French National Research Agency (ANR), and

by a public grant overseen by French ANR as part of the second ‘Investissements d’Avenir’ programme (ANR-17-RHUS-0003). This study has been financed within the framework of the Swiss HIV Cohort Study, supported by the Swiss National Science Foundation (grant #33FI-0_229621), by SHCS project #809 and #868, and by the SHCS research foundation. EuroSIDA has received funding from ViiV Healthcare LLC, United States Janssen Scientific Affairs, United States, Janssen R&D, Belgium, Bristol-Myers Squibb Company, United States, Merck Sharp & Dohme Corp, United States, Gilead Sciences, United States, and the European Union’s Seventh Framework Programme for research, technological development, and demonstration under EuroCoord grant agreement no. 260694. The participation of centers from Switzerland has been supported by the Swiss National Science Foundation (grant 148522).The study is also supported by a grant ([grant DNRF126) from the Danish National Research Foundation and by the International Cohort Consortium of Infectious Disease (RESPOND). The SHCS biobank was supported by the Liquid Biobank Inselspital Bern, Switzerland, and by the Department of Teaching and Research, Inselspital, Bern University Hospital, Switzerland. The French HIV-HBV and Biliver cohorts have been funded by the ANRS, France and Sidaction, France, as well as through an unrestricted grant from Gilead, United States. LB was supported by the «Young Talents in Clinical Research» program of the Swiss Academy of Medical Sciences and G. and J. Bangerter-Rhyner Foundation, Switzerland (grant YTCR 13/19). GW was supported by a Professorship from the Swiss National Science Foundation [PP00P3_211025]. FZ and ML received public grants overseen by the French National Research Agency (ANR) as part of the second ‘Investissements d’Avenir’ programme (reference: ANR-17-RHUS-0003) and by the EU (grant EU H2020-847939-IP-cure-B).

Conflicts of interest

LB reports unrestricted research grants from Gilead Sciences and Roche Diagnostics, support for travel and conference participation from the CROI Foundation and the SAFE-ID Foundation, and speaker honoraria from Roche, all paid to his institution. AB reports receiving speaker’s fees from Gilead Sciences, Inc. MLP reports support from the French National Research Agency (ANR). BT reports research grants paid to her institution from Aligos, Assembly, BlueJay, AusperBio, and ImCheck, and lecture honoraria from Gilead Sciences France, Hospital Vall d’Hebron and the Belgian Association for the Study of the Liver, and payment for expert testimony and travel support from the International Hepatology Education Program. CB reports travel support from Gilead and participation in the post EASL HDV advisory board 2023. FSR has no conflicts of interest to declare. CS reports honoraria for educational lectures from Abbvie and expert testimony paid to her institution from Roche Diagnostics. JKR reports grants from Gilead, paid to his institution, consulting fees from Boehringer, Gilead, MSD, ViiV, speaker honoraria from Gilead, Janssen, MSD, ViiV, participation in advisory boards from BerlinCure, and being unpaid co-chair of EuroTEST. KL reports grants from MSD, and personal funding for advisory boards and educational activities as well as support for travel and conference participation from Gilead, MSD, ViiV Healthcare. LP reports stock or stock options from Novo Nordisk, Eli Lilly and Company, and Bavarian Nordic. MH reports support from Roche, patents issued, and stock or stock options from Roche as part of employee compensation. ML reports support for travel and conference participation from Gilead, Abbvie, Madrigal, speakers honoraria from Gilead, Abbvie, and support for medical writing from Roche Diagnostics and Gilead (outside of submitted work). AR reports support to his institution for advisory boards and/or travel grants from MSD, Gilead Sciences, Pfizer, and Moderna, and an investigator-initiated trial (IIT) grant from Gilead Sciences. All remuneration went to his home institution and not to AR personally, and all remuneration was provided outside the submitted work. FZ reports research grants from Aligos, Ausperbio, Bluejay, and ImCheck, consulting fees from Aligos, Ausperbio, Bluejay, GSK, nChroma, Precision, and Gilead, and speaker honoraria from Gilead. GW reports unrestricted research grants from Gilead Sciences and Roche Diagnostics, as well as advisory board fees from MSD, ViiV, and Gilead Sciences, all paid to his institution.

Please refer to the accompanying ICMJE disclosure forms for further details.

Authors’ contributions

Study design: LB, AB, FZ, AR, GW. Performed the serological and virological analyses: MLP, BT, FSR, CS. Analyzed the data and prepared the first draft manuscript: LB. Collected and provided data for the study, contributed to the interpretation of the analyses, reviewed and commented on the draft, and approved the final version: all authors.

Data availability

Data are available upon reasonable request. The individual level datasets generated or analyzed during the current study do not fulfill the requirements for open data access as the data is too dense and comprehensive to preserve patient privacy in persons living with HIV.

Acknowledgements

The authors thank all patients, physicians, and nurses associated with the participating cohorts. The data for the SHCS are gathered by the five Swiss University Hospitals, two Cantonal Hospitals, 15 affiliated hospitals, and 36 private physicians (listed in http://www.shcs.ch/180-health-care-providers).

Members of the Swiss HIV Cohort Study. Irene A. Abela, Karoline Aebi-Popp, Alexia Anagnostopoulos, Manuel Battegay, Enos Bernasconi, Dominique L. Braun, Heiner C. Bucher, Alexandra Calmy, Matthias Cavassini (Chairman of the Clinical and Laboratory Committee), Angela Ciuffi, Günter Dollenmaier, Matthias Egger, Luisa Elzi, Jan S. Fehr, Jacques Fellay, Hansjakob Furrer, Christoph A. Fux, Huldrych F. Günthard, Anna Hachfeld, David Hans-Ulrich Haerry (Deputy of ‘Positive Council’), Barbara Hasse, Hans H. Hirsch, Matthias Hoffmann, Irene Hösli, Michael Huber, David Jackson-Perry (patient representative), Christian R. Kahlert (Chairman of the Mother & Child Substudy), Olivia Keiser, Thomas Klimkait, Roger D. Kouyos, Helen Kovari, Katharina Kusejko (Head of Data Centre), Niklaus D. Labhardt, Karoline Leuzinger, Begoña Martinez de Tejada, Catja Marzolini, Karin J. Metzner, Nicolas Müller, Johannes Nemeth, Dunja Nicca, Julia Notter, Paolo Paioni, Giuseppe Pantaleo, Matthieu Perreau, Andri Rauch (President of the SHCS), Luisa Paola Salazar-Vizcaya, Patrick Schmid, Olivier Segeral, Speck R. F, Marcel Stöckle, Philip E. Tarr, Alexandra Trkola, Gilles Wandeler (Chairman of the Scientific Board), Maja Weisser, Sabine Yerly.

Members of the EuroSIDA Study Group. The multi-centre study group, EuroSIDA (national coordinators): Albania: (A. Harxhi), University Hospital Center of Tirana, Tirana. Argentina: (M. Losso), M. Kundro, Hospital JM Ramos Mejia, Buenos Aires. Austria: (M. Knappik), P. Cichon, Klinik Penzing, Vienna; M. Sarcletti, Medical University Innsbruck, Innsbruck. Belarus: (I. Karpov), Belarusian State Medical University, Minsk; V.M. Mitsura, Gomel State Medical University, Gomel; D. Paduto, Regional Centre for Hygiene, Gomel. Belgium: (N. Clumeck), S. De Wit, M. Delforge, Saint-Pierre Hospital, Brussels; M. Frankenhuijsen, Institute of Tropical Medicine, Antwerp; M. De Scheerder, University Ziekenhuis Gent, Gent. Bosnia-Herzegovina: (J. Topalovic), Klinicki Centar Univerziteta Sarajevo, Sarajevo. Croatia: (J. Begovac), University Hospital of Infectious Diseases, Zagreb. Czech Republic: (D. Jilich), L. Machala, Faculty Hospital Bulovka, Prague; D. Sedlacek, Charles University Hospital, Plzen. Denmark: T. Benfield, Hvidovre Hospital, Copenhagen; J. Gerstoft, A.M. Lebech, O. Kirk, Rigshospitalet, Copenhagen; I.S. Johansen, Odense University Hospital, Odense; L. Ostergaard, Skejby Hospital, Aarhus; L. Wiese, Sjællands Universitetshospital, Roskilde; L.N. Nielsen, Hillerod Hospital, Hillerod, Estonia: (K. Zilmer), West-Tallinn Central Hospital, Tallinn; J. Smidt, Narva AIDS Centre, Siseklinik, Kohtla-Järve. Finland: (I. Aho), Helsinki University Central Hospital, Helsinki. France: (J.-P. Viard), The Hôtel-Dieu Hospital, Paris; K. Lacombe, Hospital Saint-Antoine, Paris; C. Pradier, E. Fontas, Hôpital de l’Archet, Nice; C. Duvivier, Hôpital Necker-Enfants Malades, Paris. Germany: (J. Rockstroh), Universitäts Klinik Bonn; O. Degen, University Medical Center Hamburg-Eppendorf, Infectious Diseases Unit, Hamburg; C. Hoffmann, ICH Study Center GmbH & Co. KG, Hamburg; C. Stefan, J.W. Goethe University Hospital, Frankfurt; J. Bogner, Medizinische Poliklinik, Munich; C. Lehmann, University Hospital Cologne, Cologne. Georgia: (A. Abutidze) Infectious Diseases, AIDS & Clinical Immunology Research Center, Tbilisi. Greece: (H. Sambatakou), Ippokration General Hospital, Athens; G. Adamis, N. Paissios, Athens General Hospital "G Gennimatas", Athens. Hungary: (J. Szlávik), South-Pest Hospital Centre, Budapest. Iceland: (M. Gottfredsson), Landspitali University Hospital, Reykjavik. Ireland: (E. Devitt), St. James’s Hospital, Dublin. Israel: (L. Tau), Ichilov Hospital, Tel Aviv; O.A. Bondarenko, L.M. Wattad, Rambam Health Care Campus, Haifa; H. Elinav, Hadassah University Hospital, Jerusalem; D. Elbirt, AIDS Center (Neve Or), Rehovot. Italy: (G. Marchetti), Ospedale San Paulo, Milan; G. Guaraldi, C. Mussini, Università Modena, Modena; A. Castagna, Ospedale San Raffaele, Milan; A. Ridolfo, F. Schiavo, Ospedale. L. Sacco, Milan. Lithuania: (V. Uzdaviniene) Vilnius University Hospital Santaros Klinikos, Vilnius; R. Matulionyte, Vilnius University, Faculty of Medicine, Department of Infectious Diseases and Dermatovenerology, Vilnius. Luxembourg: (T. Staub), R. Batutu, Centre Hospitalier, Luxembourg. Netherlands: (M. vd Valk), Academisch Medisch Centrum bij de Universiteit van Amsterdam, Amsterdam. North Macedonia (J. Trajanovska), University Clinic for Infectious Diseases & Febrile Conditions, Skopje. Norway: (D.H. Reikvam), Oslo University Hospital, Olso. Poland: (B. Knysz), B. Szetela, M. Inglot, Medical University, Wroclaw; E. Bakowska, Wojewodzki Szpital Zakazny, Warsawa; M. Parczewski, B. Aksak-Was, Pomeramian Academy of Medicine, Szczecin; M. Beniowski, E. Mularska, Szpital Specjalistycsny, Chorzow; E. Jablonowska, J. Kamerys, K. Wojcik, Wojewodzki Szpital Specjalistyczny, Lodz; I. Mozer-Lisewska, B. Rozplochowski, Poznan University of Medical Sciences, Poznan. Portugal: (A. Zagalo), Hospital Santa Maria, Lisbon; K Mansinho, Hospital de

Egas Moniz, Lisbon; F. Maltez, Hospital Curry Cabral, Lisbon. Romania: (R. Radoi), C. Oprea, Victor Babes Clinical Hospital for Infectious and Tropical Diseases, Bucharest. Russia: (D. Gusev), Medical Academy Botkin Hospital, St Petersburg; T. Trofimova, Novgorod Centre for AIDS prevention and control, Novgorod the Great; E. Kuzovatova, Academian I.N. Blokhina Nizhny Novgorod Scientific Research Institute of Epidemiology and Microbiology, Nizhny Novgorod; E. Borodulina, E. Vdoushkina, Samara State Medical University, Samara. Serbia: (J. Ranin), The Institute for Infectious and Tropical Diseases, Belgrade. Slovenia: (J.Tomazic), University Clinical Centre Ljubljana, Ljubljana. Spain: (E. Martínez), J.M. Miró, M. Laguno, J.L. Blanco, M. Martinez-Rebollar, J. Ambrosioni, B. Torres, L. de la Mora, A. Gonzalez-Cordon, I. Chivite, A. Foncillas, E. de Lazzaari, L. Berrocal, P. Callau, A. Inciarte, J. Alcami, J. Mallolas, Hospital Clinic – IDIBAPS University of Barcelona, and CIBERINFEC, S. Moreno, S. del Campo, Hospital Ramon y Cajal, Madrid; R. Paredes, J. Puig, J.R. Santos, C. Miranda, Hospital Germans Trias I Pujol, Badalona; P. Domingo, G.M. Mar, M.G. Gracia, Hospital de la Santa Creu, Barcelona; E.S. De A Arroniz, A. Ponz, Hospital Universitario de Alava, Vitoria-Gasteiz. Sweden: (C. Carlander), A. Sönnerborg, Karolinska University Hospital, Stockholm; J. Brännström, K. Falconer, Venhälsan-Sodersjukhuset, Stockholm; F. Månsson, Malmö University Hospital, Malmö. Switzerland: (K. Kusejko), D. Braun, University Hospital Zurich; M. Cavassini, University Hospital Lausanne; A. Calmy, University Hospital Geneva; H. Furrer, University Hospital Bern; M. Battegay, University Hospital Basel; P. Schmid, Cantonal Hospital St. Gallen; E. Bernasconi, Ente Ospedaliero Cantonale, Lugano. Ukraine: A. Kuznetsova, Kharkov State Medical University, Kharkov; L. Hetman, A. Kryshchuk, Public Health Center of the MOH, Kyiv. UK: M. Boffito, Chelsea and Westminster Hospital, London; S. Edwards, Mortimer Market Centre, London; F. Burns, Royal Free and University College Medical School, London; C. Orkin, Royal London Hospital, London; A. Winston, Imperial College School of Medicine at St. Mary's, London; A. Clarke, Royal Sussex County Hospital, Brighton; C. Mackintosh, Western General Hospital, Edinburgh. The following centers have previously contributed data to EuroSIDA: Medical University, Gdansk, Poland; Infectious Diseases Hospital, Sofia, Bulgaria; Hôpital de la Croix Rousse, Lyon, France; Hôpital de la Pitié-Salpétière, Paris, France; Unité INSERM, Bordeaux, France; Hôpital Edouard Herriot, Lyon, France; Bernhard Nocht Institut für Tropenmedizin, Hamburg, Germany; 1st I.K.A Hospital of Athens, Athens, Greece; Ospedale Riuniti, Divisione Malattie Infettive, Bergamo, Italy; Ospedale di Bolzano, Divisione Malattie Infettive, Bolzano, Italy; Ospedale Cotugno,III Divisione Malattie Infettive, Napoli, Italy; Dérer Hospital, Bratislava, Slovakia. Hospital Carlos III, Departamento de Enfermedades Infecciosas, Madrid, Spain; Kyiv Centre for AIDS, Kyiv, Ukraine; Luhansk State Medical University, Luhansk, Ukraine; Odessa Region AIDS Center, Odessa, Ukraine; St Petersburg AIDS Centre, St Petersburg, Russia; Infectology Centre of Latvia, Riga, Latvia; University di Roma la Sapienza, Rome, Italy; Istituto Nazionale Malattie Infettive Lazzaro Spallanzani, Rome, Italy. EuroSIDA Steering Committee: Steering Committee: C. Boesecke, C. Carlander, C. Pradier, C. Oprea, E. Martinez, G. Wandeler, I. Aho, I. Karpov, J. Begovac, J.D. Kowalska, J. Lundgren, L.D. Rasmussen, L. Tau, O. Nesterova, R. Matulionyte, S. Nozza. Chair: J.D. Kowalska. Study lead: L. Peters. EuroSIDA staff: Coordinating Centre Staff: L. Peters, J.F. Larsen, M. Gardizi, N. Jaschinski, A. Timiryasova, B. Neesgaard, F. Roper, D. Raben, A.H. Fischer. Statistical Staff: A. Cozzi-Lepri, W. Bannister. IT Staff: T.W. Elsing, L.R. Kumar, S. Shahi, B. Pepa.

Members of the French HIV/HBV and Biliver cohorts. Anders Boyd, Patrick Miailhes, Caroline Lascoux-Combe, Julie Chas, Pierre-Marie Girard, Joël Gozlan, Fabien Zoulim, Constance Delaugerre, Hayette Rougier, and Karine Lacombe. We would like to thank Lorenza N.C. Dezanet for her management of the French HIV-HBV and Biliver cohorts, particularly in relation to the Euro-B study.

Supplementary data

Supplementary data to this article can be found online at https://doi.org/10.1016/j.jhepr.2025.101671.

References

Author names in bold designate shared co-first authorship

[1] Leumi S, Bigna JJ, Amougou MA, et al. Global burden of hepatitis B infection in people living with human immunodeficiency virus: a systematic review and meta-analysis. Clin Infect Dis 2020;71:2799–2806.
[2] Platt L, French CE, McGowan CR, et al. Prevalence and burden of HBV co-infection among people living with HIV: a global systematic review and meta-analysis. J Viral Hepat 2020;27:294–315.
[3] Nikolopoulos GK, Paraskevis D, Hatzitheodorou E, et al. Impact of hepatitis B virus infection on the progression of AIDS and mortality in HIV-infected individuals: a cohort study and meta-analysis. Clin Infect Dis 2009;48:1763–1771.
[4] Hofmann E, Surial B, Boillat-Blanco N, et al. Hepatitis B virus (HBV) replication during tenofovir therapy is frequent in human immunodeficiency virus/HBV coinfection. Clin Infect Dis 2023;76:730–733.
[5] Yip TC, Wong GL, Chan HL, et al. HBsAg seroclearance further reduces hepatocellular carcinoma risk after complete viral suppression with nucleos(t)ide analogues. J Hepatol 2019;70:361–370.
[6] **Anderson RT, Choi HSJ**, Lenz O, et al. Association between seroclearance of hepatitis B surface antigen and long-term clinical outcomes of patients with chronic hepatitis B virus infection: systematic review and meta-analysis. Clin Gastroenterol Hepatol 2021;19:463–472.
[7] Yeo YH, Ho HJ, Yang HI, et al. Factors associated with rates of HBsAg seroclearance in adults with chronic HBV infection: a systematic review and meta-analysis. Gastroenterology 2019;156:635–646.e639.
[8] Boyd A, Dezanet LNC, Lacombe K. Functional cure of hepatitis B virus infection in individuals with HIV-coinfection: a literature review. Viruses 2021;13:1341.
[9] Zoutendijk R, Zaaijer HL, de Vries-Sluijs TE, et al. Hepatitis B surface antigen declines and clearance during long-term tenofovir therapy in patients coinfected with HBV and HIV. J Infect Dis 2012;206:974–980.
[10] Beguelin C, Surial B, Hofmann E, et al. Frequent hepatitis B surface antigen (HBsAg) clearance during tenofovir therapy in persons with HIV/hepatitis B virus coinfection. HIV Med 2025;26:813–819.
[11] Begre L, Boyd A, Salazar-Vizcaya L, et al. Long-term quantitative hepatitis B surface antigen (HBsAg) trajectories in persons with and without HBsAg loss on tenofovir-containing antiretroviral therapy. HIV Med 2024;25:291–298.
[12] Kimura T, Rokuhara A, Sakamoto Y, et al. Sensitive enzyme immunoassay for hepatitis B virus core-related antigens and their correlation to virus load. J Clin Microbiol 2002;40:439–445.
[13] **Testoni B, Lebosse F**, Scholtes C, et al. Serum hepatitis B core-related antigen (HBcrAg) correlates with covalently closed circular DNA transcriptional activity in chronic hepatitis B patients. J Hepatol 2019;70:615–625.
[14] Dezanet LNC, Maylin S, Gabassi A, et al. Correlation of serum hepatitis B core-related antigen with hepatitis B virus total intrahepatic DNA and covalently closed circular-DNA viral load in HIV-hepatitis B coinfection. AIDS 2020;34:1943–1949.
[15] Anderson M, Gersch J, Luk KC, et al. Circulating pregenomic hepatitis B virus RNA is primarily full-length in chronic hepatitis B patients undergoing nucleos(t)ide analogue therapy. Clin Infect Dis 2021;72:2029–2031.
[16] **Wang J, Yu Y, Li G**, et al. Relationship between serum HBV-RNA levels and intrahepatic viral as well as histologic activity markers in entecavir-treated patients. J Hepatol 2017;68:16–24.
[17] Testoni B, Scholtes C, Plissonnier ML, et al. Quantification of circulating HBV RNA expressed from intrahepatic cccDNA in untreated and NUC treated patients with chronic hepatitis B. Gut 2024;73:659–667.
[18] **Scherrer AU, Traytel A**, Braun DL, et al. Cohort profile update: the Swiss HIV cohort study (SHCS). Int J Epidemiol 2022;51:33–34j.
[19] Laut K, Kirk O, Rockstroh J, et al. The EuroSIDA study: 25 years of scientific achievements. HIV Med 2020;21:71–83.
[20] Boyd A, Gozlan J, Miailhes P, et al. Rates and determinants of hepatitis B 'e' antigen and hepatitis B surface antigen seroclearance during long-term follow-up of patients coinfected with HIV and hepatitis B virus. AIDS 2015;29:1963–1973.
[21] Begré L, Béguelin C, Boyd A, et al. Long-term trends of alanine aminotransferase levels among persons living with human immunodeficiency virus/hepatitis B virus with and without hepatitis delta coinfection. Front Med 2022;9:988356.
[22] Scholtes C, Hamilton AT, Plissonnier ML, et al. Performance of the cobas(R) HBV RNA automated investigational assay for the detection and quantification of circulating HBV RNA in chronic HBV patients. J Clin Virol 2022;150–151:105150.
[23] van Bremen K, Hoffmann C, Mauss S, et al. Obstacles to HBV functional cure: late presentation in HIV and its impact on HBV seroconversion in HIV/HBV coinfection. Liver Int 2020;40:2978–2981.
[24] Xia H, Gao L, Hu Y, et al. High rates of hepatitis B virus (HBV) functional cure among HIV/HBV coinfected Chinese adults on antiretroviral therapy. Chin Med J (Engl) 2022;135:2744–2746.

[25] Audsley J, Avihingsanon A, Littlejohn M, et al. Long-term TDF-inclusive ART and progressive rates of HBsAg loss in HIV-HBV coinfection-lessons for functional HBV cure? J Acquir Immune Defic Syndr 2020;84:527–533.
[26] Vinikoor MJ, Hamusonde K, Muula G, et al. Long-term hepatitis B and liver outcomes among adults taking tenofovir-containing antiretroviral therapy for HBV/HIV coinfection in Zambia. Clin Infect Dis 2024;78:1583–1590.
[27] Chihota BV, Wandeler G, Chilengi R, et al. High rates of hepatitis B virus (HBV) functional cure among human immunodeficiency virus-HBV coinfected patients on antiretroviral therapy in Zambia. J Infect Dis 2020;221:218–222.
[28] Iannacone M, Guidotti LG. Immunobiology and pathogenesis of hepatitis B virus infection. Nat Rev Immunol 2022;22:19–32.
[29] Boni C, Laccabue D, Lampertico P, et al. Restored function of HBV-specific T cells after long-term effective therapy with nucleos(t)ide analogues. Gastroenterology 2012;143:963–973.e969.
[30] Begre L, Boyd A, Plissonnier ML, et al. Circulating HBV RNA and hepatitis B core-related antigen trajectories in persons with HIV/HBV coinfection and hepatitis B surface antigen loss during tenofovir therapy. J Infect Dis 2024;230:e954–e963.
[31] Chung RT, King WC, Ghany MG, et al. A prospective cohort study of novel markers of hepatitis B virus replication in human immunodeficiency virus coinfection. Clin Gastroenterol Hepatol 2023;21:125–135.e128.
[32] Suslov A, Meier MA, Ketterer S, et al. Transition to HBeAg-negative chronic hepatitis B virus infection is associated with reduced cccDNA transcriptional activity. J Hepatol 2021;74:794–800.
[33] Hume SJ, Wong DK, Yuen MF, et al. High end-of-treatment hepatitis B core-related antigen levels predict hepatitis flare after stopping nucleot(s)ide analogue therapy. Liver Int 2024;44:2605–2614.
[34] Sonneveld MJ, Park JY, Kaewdech A, et al. Prediction of sustained response after nucleo(s)tide analogue cessation using HBsAg and HBcrAg levels: a multicenter study (CREATE). Clin Gastroenterol Hepatol 2022;20:e784–e793.
[35] Terrault NA, Sterling R, Lok AS, et al. Hepatitis B virus RNA as a biomarker for safe antiviral discontinuation: a prospective study of nucleos(t)ide analogue withdrawal. J Infect Dis 2025;231:1290–1298.
[36] **van Bommel F**, **Bartens A**, et al. Serum hepatitis B virus RNA levels as an early predictor of hepatitis B envelope antigen seroconversion during treatment with polymerase inhibitors. Hepatology 2015;61:66–76.
[37] Butler EK, Gersch J, McNamara A, et al. Hepatitis B virus serum DNA and RNA levels in nucleos(t)ide analog-treated or untreated patients during chronic and acute infection. Hepatology 2018;68:2106–2117.
[38] Paturel A, Casuscelli di Tocco F, Bousquet D, et al. A molecular standard for circulating HBV RNA detection and quantification assays in patients with chronic hepatitis B. JHEP Rep 2024;6:101124.
[39] Inoue T, Kusumoto S, Iio E, et al. Clinical efficacy of a novel, high-sensitivity HBcrAg assay in the management of chronic hepatitis B and HBV reactivation. J Hepatol 2021;75:302–310.
[40] Chen J, Li L, Yin Q, et al. A review of epidemiology and clinical relevance of Hepatitis B virus genotypes and subgenotypes. Clin Res Hepatol Gastroenterol 2023;47:102180.

Keywords: Hepatitis B virus; HIV; Coinfection; HBV RNA; Hepatitis B core-related antigen; Cohort studies; Tenofovir.
Received 20 June 2025; received in revised form 26 September 2025; accepted 30 October 2025; Available online 7 November 2025

Journal of Hepatology, Volume *8*

Supplemental information

Circulating HBV RNA and hepatitis B core-related antigen as determinants of HBsAg loss in persons with HIV in Europe

Lorin Begré, Anders Boyd, Marie-Laure Plissonnier, Barbara Testoni, Charles Béguelin, Franziska Suter-Riniker, Caroline Scholtès, Jürgen K. Rockstroh, Karine Lacombe, Lars Peters, Marintha Heil, Massimo Levrero, Andri Rauch, Fabien Zoulim, Gilles Wandeler, for the Swiss HIV Cohort Study, EuroSIDA and French HIV/HBV and Biliver cohorts, Members of the EuroSIDA Study Group, and Members of the French HIV/HBV and Biliver cohorts

Circulating HBV RNA and hepatitis B core-related antigen as determinants of HBsAg loss in persons with HIV in Europe

Lorin Begré, Anders Boyd, Marie-Laure Plissonnier, Barbara Testoni, Charles Béguelin, Franziska Suter-Riniker, Caroline Scholtès, Jürgen K. Rockstroh, Karine Lacombe, Lars Peters, Marintha Heil, Massimo Levrero, Andri Rauch, Fabien Zoulim, Gilles Wandeler, the Swiss HIV Cohort study, EuroSIDA and French HIV/HBV and Biliver cohorts

Table of contents

Fig. S1 2
Fig. S2. 3
Fig. S3 4
Fig. S4 5
Fig. S5 6
Fig. S6 7
Table S1 8
Table S2 9
Table S3. 11
Table S4. 12
Table S5 13
Table S6 14
Table S7 15
Table S8 16
Table S9 17

Supplementary Figures

Fig. S1: Inclusion flow chart.

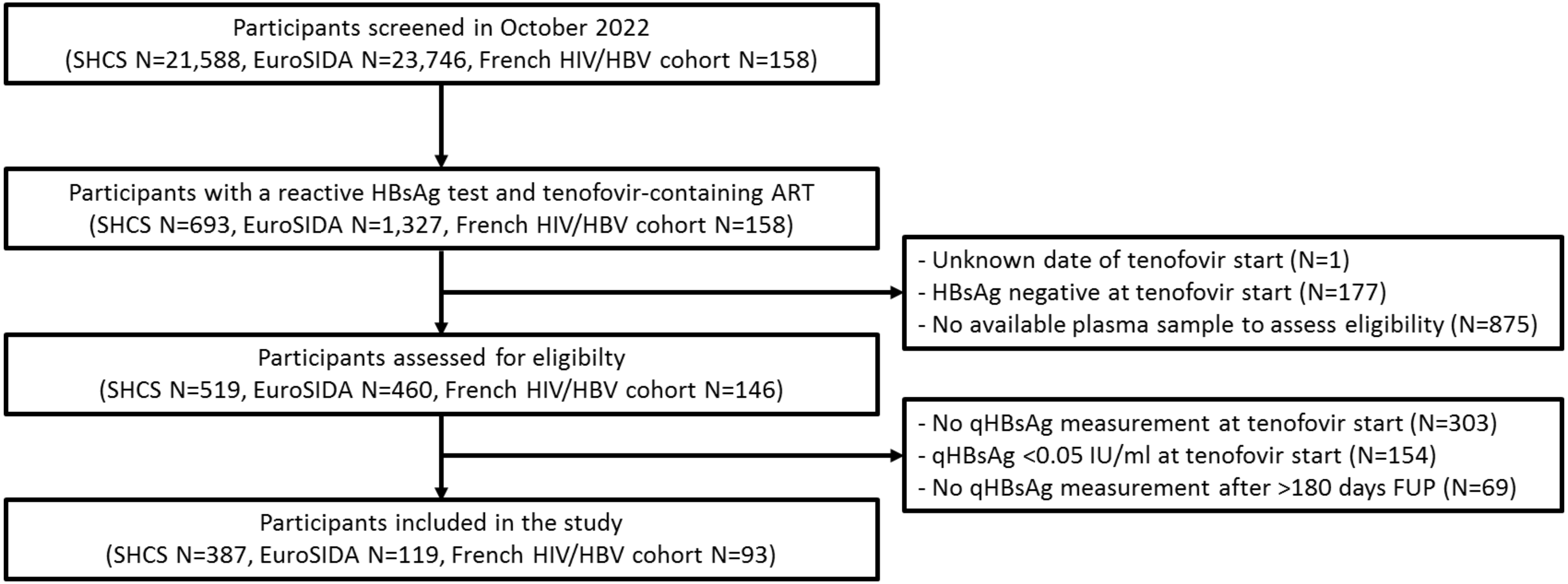

Abbreviations: ART, antiretroviral therapy; FUP, follow-up; HBsAg, hepatitis B surface antigen; HBV, hepatitis B virus; HIV, human immunodeficiency virus; qHBsAg, quantitative HBsAg; SHCS, Swiss HIV Cohort Study.

Fig. S2: qHBsAg levels in HBeAg-negative and HBeAg-positive participants at start of tenofovir therapy, after two years of follow-up and at the last follow-up visit, stratified by prior treatment with anti-HBV activity*.

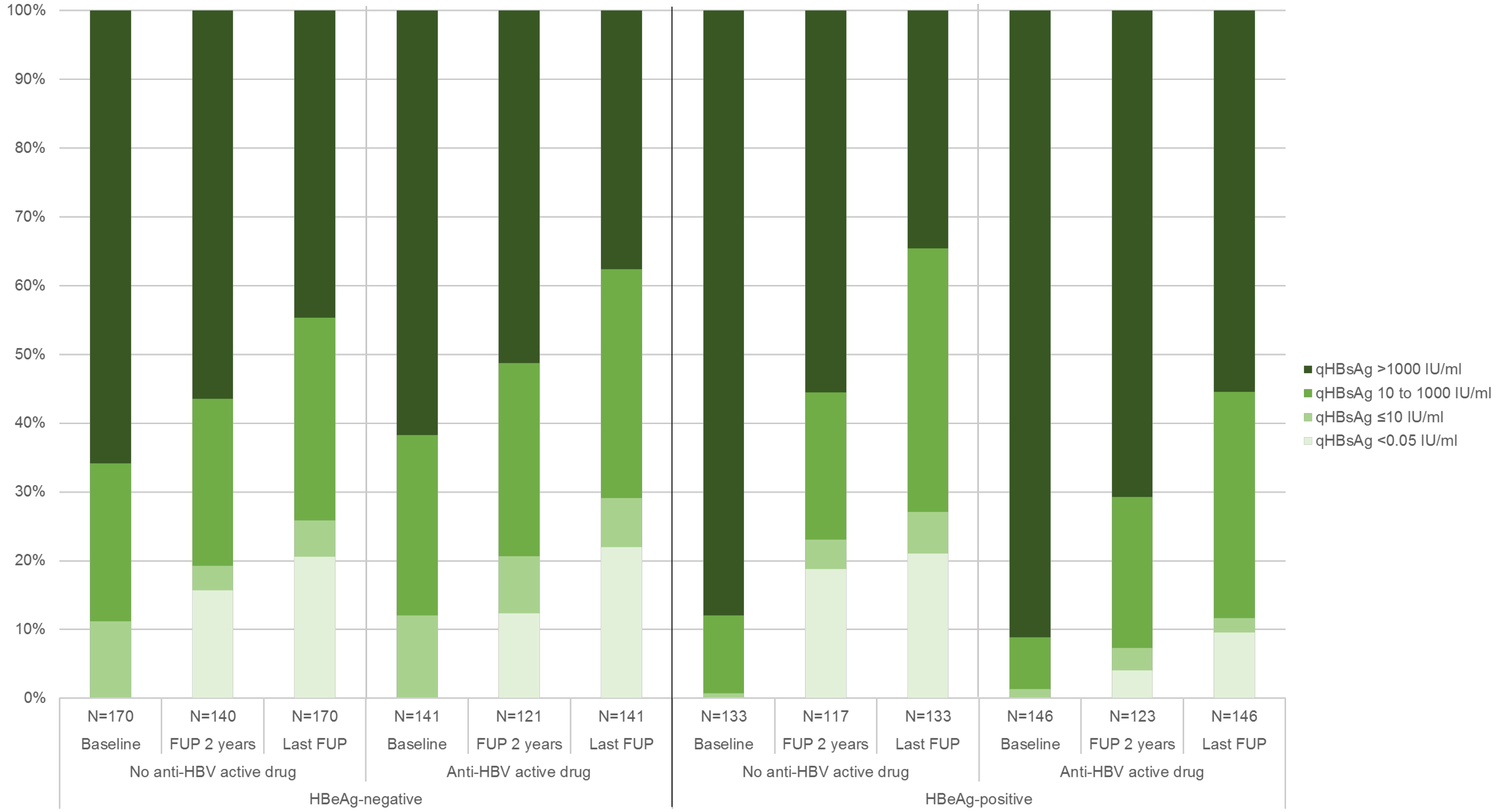

*Anti-HBV active drugs included lamivudine, emtricitabine, entecavir, adefovir and interferon-alfa. Abbreviations: ART, antiretroviral therapy; FUP, follow-up; HBeAg, IU/ml, international units per milliliter; qHBsAg, quantitative hepatitis B surface antigen.

Fig. S3: HBV DNA levels in HBeAg-negative and HBeAg-positive participants at start of tenofovir therapy, after two years of follow-up and at the last follow-up visit, stratified by prior treatment with anti-HBV activity*.

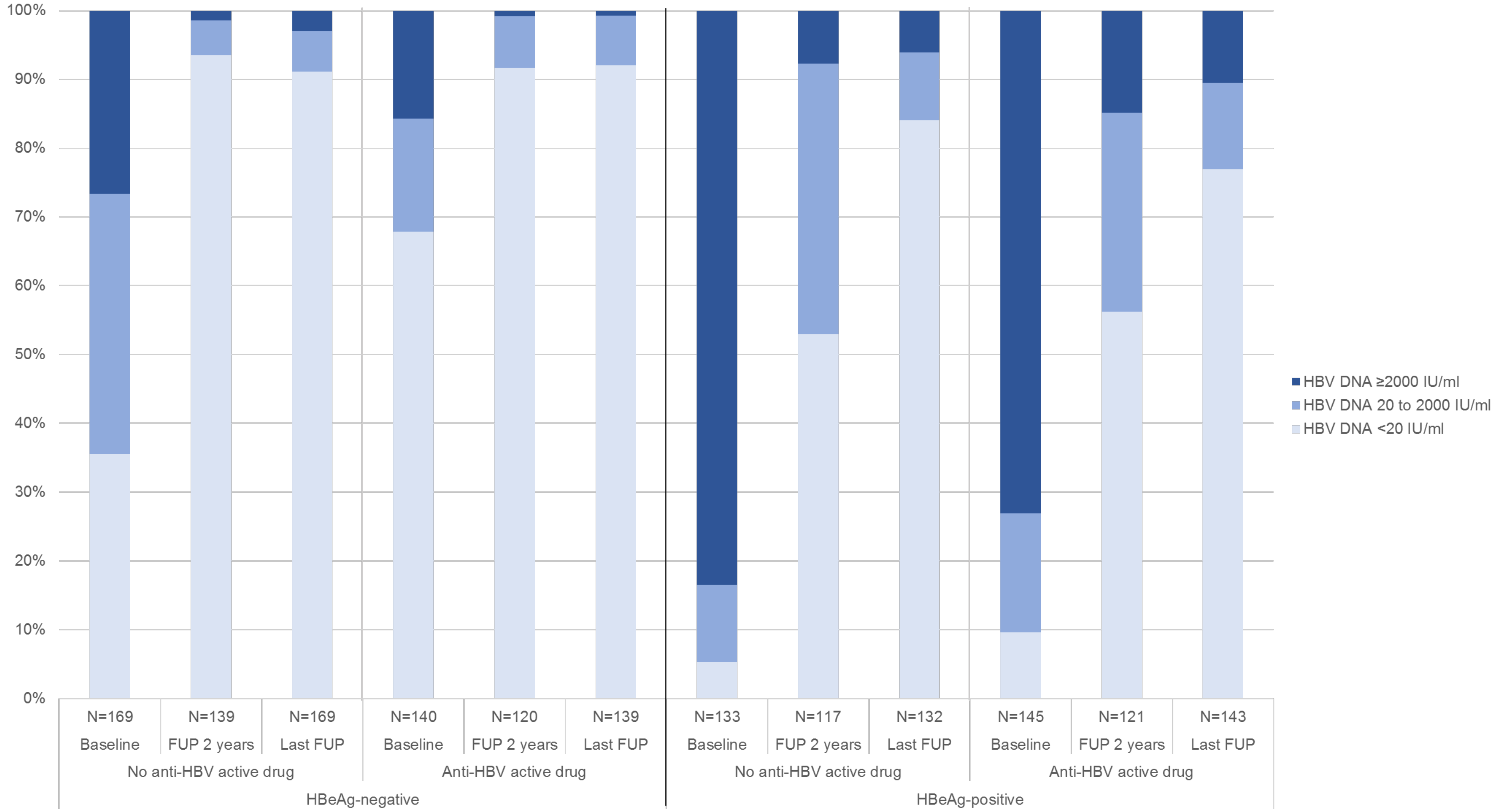

*Anti-HBV active drugs included lamivudine, emtricitabine, entecavir, adefovir and interferon-alfa. Abbreviations: ART, antiretroviral therapy; DNA, deoxyribonucleic acid; FUP, follow-up; HBeAg, hepatitis B e antigen; IU/ml, international units per milliliter; HBV, hepatitis B virus.

Fig. S4: HBcrAg levels in HBeAg-negative and HBeAg-positive participants at start of tenofovir therapy, after two years of follow-up and at the last follow-up visit, stratified by prior treatment with anti-HBV activity*.

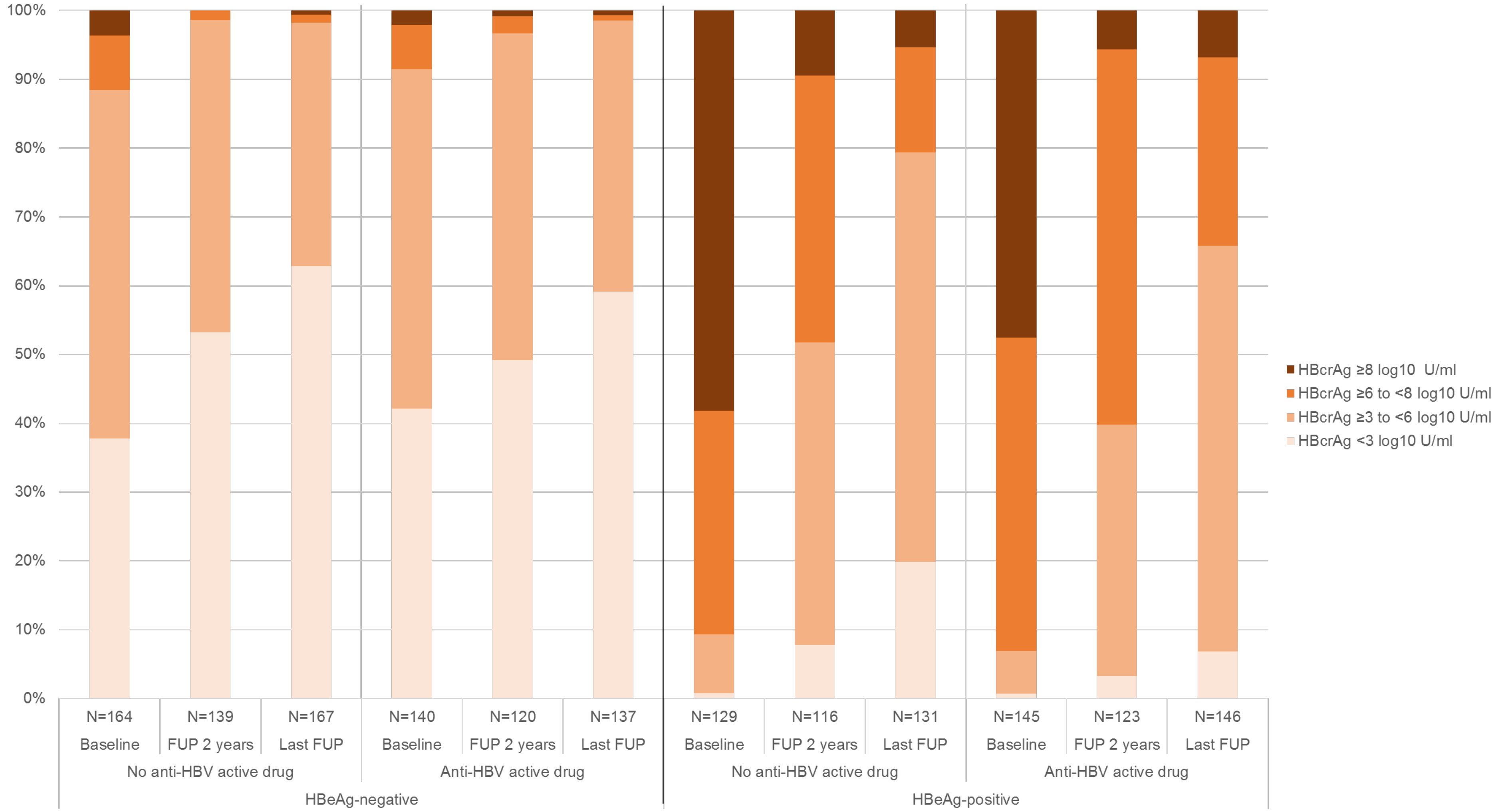

*Anti-HBV active drugs included lamivudine, emtricitabine, entecavir, adefovir and interferon-alfa. Abbreviations: ART, antiretroviral therapy; FUP, follow-up; HBcrAg, hepatitis B core-related antigen; HBeAg, hepatitis B e antigen; U/ml, units per milliliter

Fig. S5: HBV RNA levels in HBeAg-negative and HBeAg-positive participants at start of tenofovir therapy, after two years of follow-up and at the last follow-up visit, stratified by prior treatment with anti-HBV activity*.

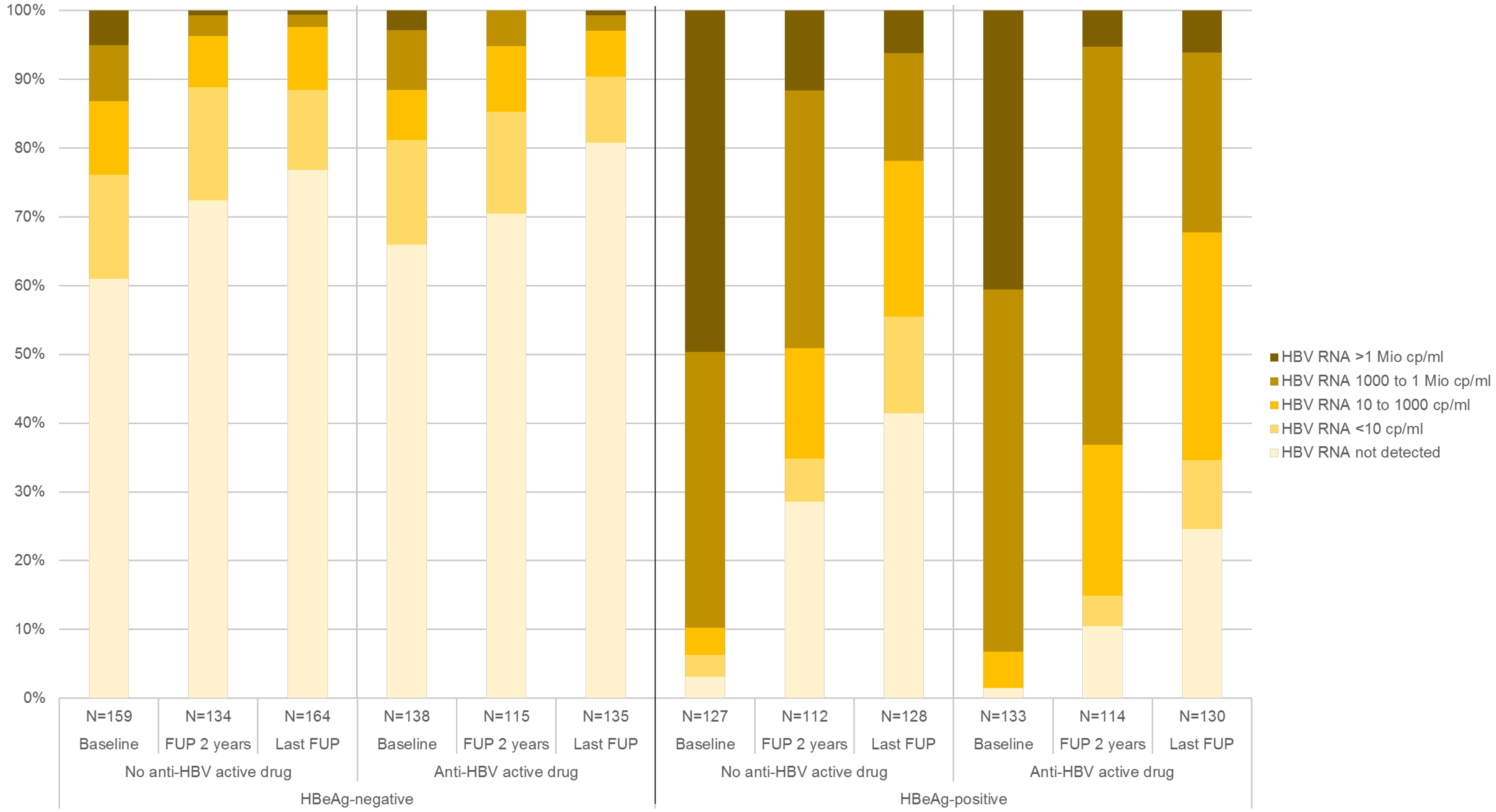

*Anti-HBV active drugs included lamivudine, emtricitabine, entecavir, adefovir and interferon-alfa. Abbreviations: ART, antiretroviral therapy; cp/ml, copies per milliliter; FUP, follow-up; HBeAg, hepatitis B e antigen Mio, million; HBV, hepatitis B virus; RNA, ribonucleic acid.

Fig. S6: Proportion of participants with HBV DNA <20 IU/ml, HBcrAg <3 log_{10} U/ml and HBV RNA below the detection limit after two years of tenofovir-containing antiretroviral therapy, stratified by qHBsAg level loss and HBeAg status.

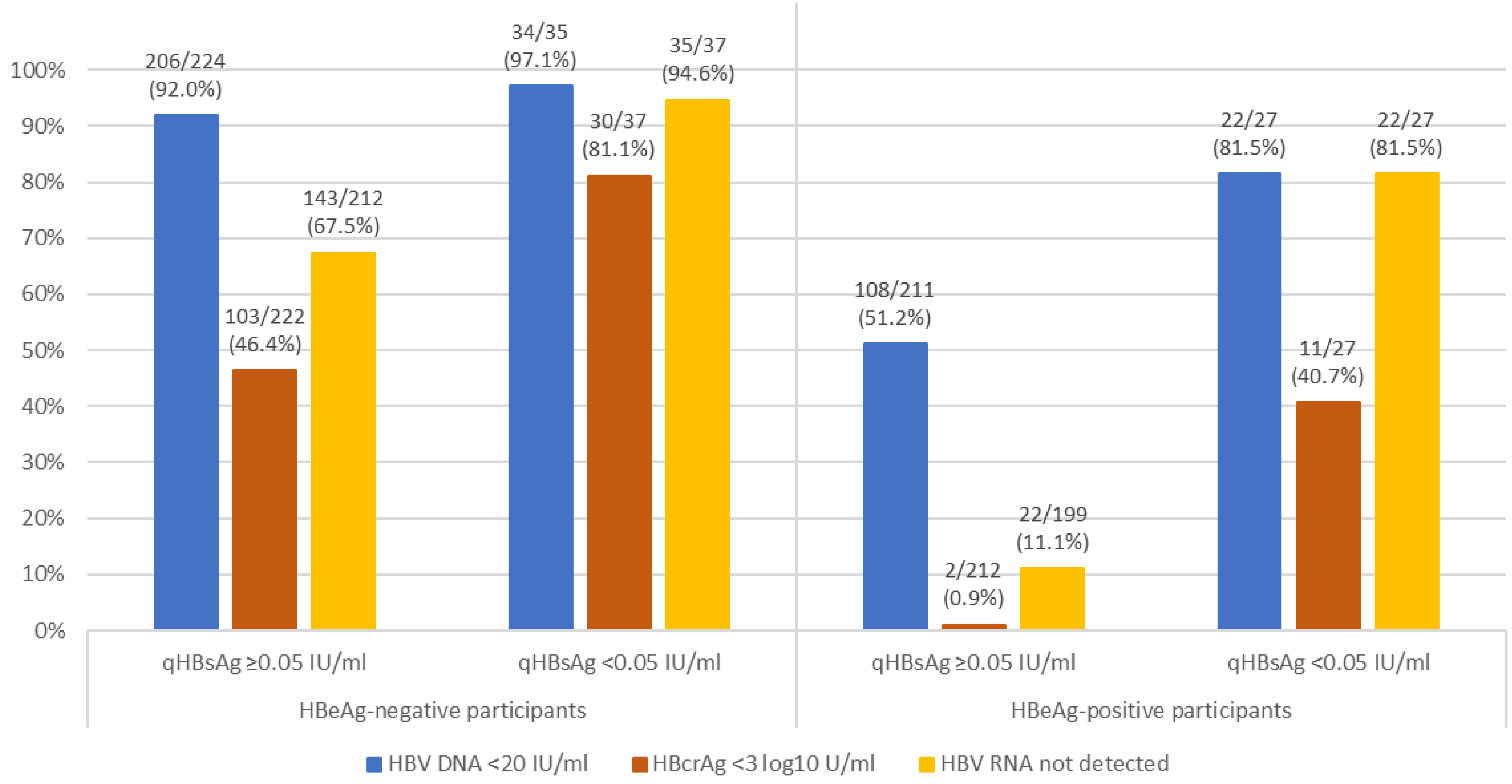

Abbreviations: DNA, deoxyribonucleic acid; HBcrAg, hepatitis B core-related antigen; HBeAg, hepatitis B e antigen; HBV, hepatitis B virus; IU/ml, international units milliliter; qHBsAg, quantitative hepatitis B surface antigen; RNA, ribonucleic acid; U/ml, units per milliliter.

Supplementary Tables

Table S1: Baseline characteristics of participants with an HBsAg-positive test before commencing tenofovir who were included and not included in the analysis.

	**Excluded with reason N=526**	**Included in analysis N=599**	**P-value**
Female sex at birth	92/515 (17.9%)	110/599 (18.4%)	0.83
Age, median (IQR), years	41.0 (35.0-46.0)	41.0 (35.0-47.0)	0.55
Calendar year of tenofovir start, median (IQR)	2006 (2003-2009)	2006 (2003-2009)	0.08
HBV prevalence ≥2% in country of origin*	115/511 (22.5%)	174/596 (29.2%)	**0.01**
BMI, median (IQR), kg/m^2	23.0 (20.7-25.2)	22.8 (20.9-25.3)	>0.99
On ART at start of tenofovir	280/526 (53.2%)	345/599 (57.6%)	0.14
CD4 count, median (IQR), cells/mm^3	323 (201-525)	345 (215-513)	0.41
CD4 nadir, median (IQR), cells/mm^3	161 (59-267)	170 (61-288)	0.20
HIV viral load <50 copies/ml	212/526 (40.3%)	259/599 (43.2%)	0.32
AIDS-defining condition†	151/526 (28.7%)	151/599 (25.2%)	0.19
Liver cirrhosis	31/242 (12.8%)	50/366 (13.7%)	0.76
Ever unhealthy alcohol use	40/139 (28.8%)	41/292 (14.0%)	**<0.001**

*Based on 2015 estimates from GBD 2019 Hepatitis Collaborators, Lancet Gastroenterol Hepatol 2022; 7:796-829. †According to the clinical classification of HIV disease by the US Centers for Disease Control and Prevention. Data are presented as median (IQR) for continuous measures, and n/total (%) for categorical measures. Continuous variables were compared using Wilcoxon rank-sum tests. Comparisons of categorical variables were performed using Pearson's χ^2 tests. Level of significance: $p<0.05$ (bold). Abbreviations: BMI, body mass index; HBV, hepatitis B virus; IQR, interquartile range.

Table S2: Characteristics of participants at baseline, stratified by HBeAg and ART status.

	**HBeAg-negative participants**			**HBeAg-positive participants**		
	**Not on ART**<br>**N=141**	**ART-experienced**<br>**N=170**	**P-value**	**Not on ART**<br>**N=106**	**ART-experienced**<br>**N=173**	**P-value**
Female sex at birth	41/141 (29.1%)	44/170 (25.9%)	0.53	11/106 (10.4%)	13/173 (7.5%)	0.41
Age, median (IQR), years	40.0 (34.0-46.0)	41.0 (36.0-49.0)	0.07	39.5 (33.0-45.0)	42.0 (37.0-47.0)	**0.03**
Calendar year of tenofovir start, median (IQR)	2009 (2006-2013)	2005 (2003-2008)	**<0.001**	2007 (2004-2010)	2003 (2002-2005)	**<0.001**
HBV prevalence ≥2% in country of origin*	60/140 (42.9%)	64/169 (37.9%)	0.37	21/106 (19.8%)	27/172 (15.7%)	0.38
Mode of HIV acquisition			0.06			0.06
Men who have sex with men	49/141 (34.8%)	68/170 (40.0%)		67/106 (63.2%)	120/173 (69.4%)	
Heterosexual contact	63/141 (44.7%)	52/170 (30.6%)		22/106 (20.8%)	25/173 (14.5%)	
Injection drug use	19/141 (13.5%)	30/170 (17.6%)		14/106 (13.2%)	13/173 (7.5%)	
Other or unknown	10/141 (7.1%)	20/170 (11.8%)		3/106 (2.8%)	15/173 (8.7%)	
BMI, median (IQR), kg/m^2	23.8 (21.4-26.8)	22.5 (20.8-25.8)	0.07	22.7 (21.1-24.3)	22.6 (20.5-24.3)	0.50
On lamivudine or emtriticabine	0/141 (0.0%)	138/170 (81.2%)	**<0.001**	0/106 (0.0%)	146/173 (84.4%)	**<0.001**
On adefovir or entecavir	1/141 (0.7%)	2/170 (1.2%)	0.67	0/106 (0.0%)	4/173 (2.3%)	0.11
On interferon-α therapy	1/141 (0.7%)	3/170 (1.8%)	0.41	0/106 (0.0%)	0/173 (0.0%)	
CD4 count, median (IQR), $cells/mm^3$	338 (184-443)	415 (271-564)	**<0.001**	288 (181-413)	404 (265-590)	**<0.001**
CD4 nadir, median (IQR), $cells/mm^3$	234 (130-351)	145 (52-241)	**<0.001**	232 (96-320)	109 (37-228)	**<0.001**
HIV viral load <50 copies/ml	31/141 (22.0%)	123/170 (72.4%)	**<0.001**	22/106 (20.8%)	120/173 (69.4%)	**<0.001**
AIDS-defining condition†	16/141 (11.3%)	44/170 (25.9%)	**0.001**	24/106 (22.6%)	66/173 (38.2%)	**0.007**

Liver cirrhosis	10/87 (11.5%)	18/109 (16.5%)	0.32	14/67 (20.9%)	8/98 (8.2%)	**0.02**
Ever unhealthy alcohol use	13/73 (17.8%)	8/78 (10.3%)	0.18	10/58 (17.2%)	9/79 (11.4%)	0.33
HBV DNA, median (IQR), $\log_{10}$ IU/ml	2.1 (0.0-3.6)	0.0 (0.0-2.1)	**<0.001**	7.0 (4.1-8.1)	6.5 (3.8-8.0)	0.32
HBV DNA <20 IU/ml	48/141 (34.0%)	107/168 (63.7%)		7/106 (6.6%)	14/172 (8.1%)	
qHBsAg, median (IQR), $\log_{10}$ IU/ml	3.5 (2.3-4.0)	3.3 (2.5-3.8)	0.06	4.5 (3.5-5.0)	4.7 (4.1-5.0)	**0.04**
qHBsAg >1000 IU/ml	91/141 (64.5%)	108/170 (63.5%)		90/106 (84.9%)	160/173 (92.5%)	
qHBsAg >10 to 1000 IU/ml	33/141 (23.4%)	43/170 (25.3%)		15/106 (14.2%)	11/173 (6.4%)	
qHBsAg ≤10 IU/ml	17/141 (12.1%)	19/170 (11.2%)		1/106 (0.9%)	2/173 (1.2%)	
HBcrAg, median (IQR), $\log_{10}$ U/ml	3.4 (2.9-4.6)	3.1 (2.9-4.3)	0.24	8.0 (7.1-8.5)	8.0 (7.2-8.5)	0.91
HBcrAg <3 $\log_{10}$ U/ml	49/136 (36.0%)	72/168 (42.9%)	0.23	1/103 (1.0%)	1/171 (0.6%)	0.72
HBV RNA, median (IQR), $\log_{10}$ copies/ml	0.0 (0.0-0.8)	0.0 (0.0-0.8)	0.49	5.9 (5.1-6.4)	5.9 (5.1-6.3)	0.71
HBV RNA <LLOD	83/134 (61.9%)	105/163 (64.4%)	0.66	4/102 (3.9%)	2/158 (1.3%)	0.16
Ever Hepatitis D antibody positive	23/124 (18.5%)	31/152 (20.4%)	0.70	7/95 (7.4%)	11/159 (6.9%)	0.89
Ever Hepatitis D RNA positive	14/122 (11.5%)	15/149 (10.1%)	0.71	4/96 (4.2%)	7/158 (4.4%)	0.92
Hepatitis C antibody positive	15/124 (12.1%)	27/159 (17.0%)	0.25	13/95 (13.7%)	10/153 (6.5%)	0.06
Hepatitis C RNA positive	10/122 (8.2%)	13/154 (8.4%)	0.94	9/91 (9.9%)	2/148 (1.4%)	**0.002**
ALT elevation ≥5x ULN	8/136 (5.9%)	6/168 (3.6%)	0.34	6/103 (5.8%)	18/170 (10.6%)	0.18

*Based on 2015 estimates from GBD 2019 Hepatitis Collaborators, Lancet Gastroenterol Hepatol 2022; 7:796-829. †According to the clinical classification of HIV disease by the US Centers for Disease Control and Prevention. Data are presented as median (IQR) for continuous measures, and n/total (%) for categorical measures. Continuous variables were compared using Wilcoxon rank-sum tests. Comparisons of categorical variables were performed using Pearson's χ^2 tests. Level of significance: $p<0.05$ (bold). Abbreviations: AIDS, acquired immunodeficiency syndrome; ALT, alanine aminotransferase; BMI, body mass index; ART, antiretroviral therapy; DNA, deoxyribonucleic acid; HBcrAg, hepatitis B core-related antigen; HBeAg, hepatitis B e antigen; HBV, hepatitis B virus; IQR, interquartile range; LLOD, lower limit of detection; qHBsAg, quantitative hepatitis B surface antigen; RNA, ribonucleic acid; ULN, upper limit of normal.

Table S3: Determinants of HBsAg loss at the last follow-up visit among HBeAg-negative participants.

		Univariable analysis		Multivariable analysis			
				with HBcrAg (N=296)		with HBV RNA (N=290)	
	N	OR (95% CI)	P-value	OR (95% CI)	P-value	OR (95% CI)	P-value
qHBsAg ≤1000 IU/ml	311	5.69 (3.16-10.26)	**<0.001**	7.64 (3.78-15.44)	**<0.001**	6.82 (3.39-13.70)	**<0.001**
HBV DNA, per 1 log_{10} IU/ml increase	309	0.93 (0.81-1.06)	0.25	0.94 (0.77-1.15)	0.55	1.05 (0.85-1.29)	0.67
HBV RNA, per 1 log_{10} cp/ml increase	297	0.90 (0.76-1.08)	0.26			0.98 (0.75-1.29)	0.89
HBcrAg, per 1 log_{10} U/ml increase	304	1.01 (0.83-1.22)	0.93	1.25 (0.92-1.71)	0.16		
ALT, per 10 IU/ml increase	304	1.03 (1.00-1.06)	0.10	1.03 (0.99-1.07)	0.13	1.04 (1.00-1.08)	**0.05**
Age, per 10 years	311	1.23 (0.94-1.61)	0.12	1.16 (0.84-1.60)	0.36	1.18 (0.85-1.62)	0.32
Female sex at birth	311	0.74 (0.39-1.39)	0.35	1.29 (0.62-2.70)	0.50	1.26 (0.60-2.64)	0.54
CD4 <200 cells/ul	309	1.05 (0.53-2.09)	0.89	0.95 (0.42-2.18)	0.91	1.15 (0.49-2.68)	0.75
ART-experienced	311	0.92 (0.53-1.59)	0.76	0.75 (0.39-1.46)	0.40	0.83 (0.42-1.61)	0.58
Follow-up time, per 1 log_{10} years	311	3.03 (1.32-6.93)	**0.01**	3.13 (1.24-7.86)	**0.02**	3.07 (1.22-7.75)	**0.02**

The determinants of HBsAg loss were assessed using univariable and multivariable logistic regression models. Wald tests were performed to assess statistical significance of individual covariables with significance being defined as p<0.05 (bold). Abbreviations: ALT, alanine aminotransferase; ART, antiretroviral therapy; CI, confidence interval; cp/ml, copies per milliliter; DNA, deoxyribonucleic acid; HBcrAg, hepatitis B core-related antigen; HBeAg, hepatitis B e antigen; HBV, hepatitis B virus; IU/ml, IU/l, international units per liter; international units per milliliter; OR, odds ratio; qHBsAg, quantitative hepatitis B surface antigen; RNA, ribonucleic acid; U/ml, units per milliliter.

Table S4: Determinants of HBsAg loss at the last follow-up visit among HBeAg-positive participants.

		Univariable analysis		Multivariable analysis			
				with HBcrAg (N=268)		with HBV RNA (N=255)	
	N	**OR (95% CI)**	**P-value**	**OR (95% CI)**	**P-value**	**OR (95% CI)**	**P-value**
qHBsAg ≤1000 IU/ml	279	4.32 (1.87-9.99)	**0.001**	4.82 (1.53-15.21)	**0.007**	4.98 (1.59-15.58)	**0.006**
HBV DNA, per 1 log_{10} IU/ml increase	278	1.08 (0.94-1.23)	0.27	1.31 (1.06-1.60)	**0.01**	1.42 (1.13-1.78)	**0.003**
HBV RNA, per 1 log_{10} cp/ml increase	260	0.76 (0.63-0.90)	**0.002**			0.66 (0.50-0.88)	**0.005**
HBcrAg, per 1 log_{10} U/ml increase	274	0.77 (0.59-1.00)	0.05	0.74 (0.50-1.09)	0.13		
ALT, per 10 IU/ml increase	273	0.99 (0.97-1.02)	0.62	1.00 (0.97-1.02)	0.67	1.00 (0.97-1.02)	0.71
Age, per 10 years	279	1.15 (0.77-1.70)	0.49	1.38 (0.88-2.15)	0.16	1.44 (0.92-2.26)	0.11
Female sex at birth	279	2.03 (0.75-5.45)	0.16	2.04 (0.64-6.52)	0.23	1.94 (0.59-6.37)	0.28
CD4 <200 cells/ul	279	1.75 (0.83-3.70)	0.14	1.11 (0.45-2.74)	0.83	1.15 (0.46-2.90)	0.76
ART-experienced	279	0.35 (0.18-0.69)	**0.002**	0.39 (0.18-0.86)	**0.02**	0.45 (0.20-1.00)	0.05
Follow-up time, per 1 log_{10} years	279	3.32 (1.13-9.78)	**0.03**	3.42 (1.06-11.05	**0.04**	2.58 (0.77-8.58)	0.12

The determinants of HBsAg loss were assessed using univariable and multivariable logistic regression models. Wald tests were performed to assess statistical significance of individual covariables with significance being defined as p<0.05 (bold). Abbreviations: ALT, alanine aminotransferase; ART, antiretroviral therapy; CI, confidence interval; cp/ml, copies per milliliter; DNA, deoxyribonucleic acid; HBcrAg, hepatitis B core-related antigen; HBeAg, hepatitis B e antigen; HBV, hepatitis B virus; IU/l, international units per liter; IU/ml, international units per milliliter; OR, odds ratio; qHBsAg, quantitative hepatitis B surface antigen; RNA, ribonucleic acid; U/ml, units per milliliter.

Table S5: Determinants of HBsAg loss after two years of tenofovir-containing antiretroviral therapy among HBeAg-negative participants.

		Univariable analysis		Multivariable analysis			
				with HBcrAg (N=248)		with HBV RNA (N=242)	
	N	**OR (95% CI)**	**P-value**	**OR (95% CI)**	**P-value**	**OR (95% CI)**	**P-value**
qHBsAg ≤1000 IU/ml	261	7.29 (3.26-16.28)	**<0.001**	22.82 (6.99-74.54)	**<0.001**	19.91 (6.21-63.85)	**<0.001**
HBV DNA, per 1 $\log_{10}$ IU/ml increase	259	1.06 (0.91-1.23)	0.47	0.99 (0.76-1.28))	0.91	1.11 (0.86-1.44)	0.42
HBV RNA, per 1 $\log_{10}$ cp/ml increase	248	1.05 (0.87-1.26)	0.62			1.30 (0.93-1.84)	0.13
HBcrAg, per 1 $\log_{10}$ U/ml increase	255	1.19 (0.96-1.49)	0.12	1.79 (1.16-2.76)	**0.008**		
ALT, per 10 IU/ml increase	254	1.03 (1.00-1.06)	0.07	1.03 (0.99-1.07)	0.11	1.04 (1.01-1.08)	**0.03**
Age, per 10 years	261	1.18 (0.83-1.67)	0.35	1.19 (0.77-1.86)	0.43	1.18 (0.77-1.82)	0.45
Female sex at birth	261	1.55 (0.74-3.24)	0.24	5.37 (1.93-14.93)	**0.001**	4.68 (1.71-12.82)	**0.003**
CD4 <200 cells/ul	260	1.22 (0.52-2.87)	0.64	0.75 (0.25-2.21)	0.60	0.91 (0.31-2.65)	0.86
ART-experienced	261	0.72 (0.36-1.45)	0.36	0.75 (0.30-1.84)	0.53	0.92 (0.37-2.26)	0.85

The determinants of HBsAg loss were assessed using univariable and multivariable logistic regression models. Wald tests were performed to assess statistical significance of individual covariables with significance being defined as p<0.05 (bold). Abbreviations: ALT, alanine aminotransferase; ART, antiretroviral therapy; CI, confidence interval; cp/ml, copies per milliliter; DNA, deoxyribonucleic acid; HBcrAg, hepatitis B core-related antigen; HBeAg, hepatitis B e antigen; HBV, hepatitis B virus; IU/l, international units per liter; IU/ml, international units per milliliter; OR, odds ratio; qHBsAg, quantitative hepatitis B surface antigen; RNA, ribonucleic acid; U/ml, units per milliliter.

Table S6: Determinants of HBsAg loss after two years of tenofovir-containing antiretroviral therapy among HBeAg-positive participants.

		Univariable analysis		Multivariable analysis			
				with HBcrAg (N=230)		with HBV RNA (N=219)	
	N	OR (95% CI)	P-value	OR (95% CI)	P-value	OR (95% CI)	P-value
qHBsAg ≤1000 IU/ml	240	3.57 (1.34-9.53)	**0.01**	1.43 (0.41-5.09)	0.58	1.77 (0.50-6.25)	0.38
HBV DNA, per 1 log_{10} IU/ml increase	239	0.92 (0.79-1.07)	0.28	1.04 (0.84-1.28)	0.75	1.12 (0.88-1.41)	0.36
HBV RNA, per 1 log_{10} cp/ml increase	223	0.64 (0.53-0.79)	**<0.001**			0.64 (0.47-0.85)	**0.003**
HBcrAg, per 1 log_{10} U/ml increase	235	0.63 (0.47-0.85)	**0.003**	0.66 (0.44-1.00)	**0.05**		
ALT, per 10 IU/l increase	235	1.00 (0.98-1.03)	0.86	1.01 (0.98-1.03)	0.60	1.00 (0.98-1.03)	0.64
Age, per 10 years	240	0.94 (0.58-1.52)	0.79	1.15 (0.67-1.96)	0.61	1.22 (0.71-2.09)	0.47
Female sex at birth	240	1.88 (0.59-6.05)	0.29	2.04 (0.54-7.72)	0.30	1.88 (0.49-7.29)	0.36
CD4 <200 cells/µl	240	1.47 (0.58-3.71)	0.42	0.78 (0.26-2.36)	0.66	0.81 (0.26-2.51)	0.72
ART-experienced	240	0.22 (0.09-0.54)	**0.001**	0.22 (0.08-0.58	**0.002**	0.27 (0.10-0.74)	**0.01**

The determinants of HBsAg loss were assessed using univariable and multivariable logistic regression models. Wald tests were performed to assess statistical significance of individual covariables with significance being defined as p<0.05 (bold). Abbreviations: ALT, alanine aminotransferase; ART, antiretroviral therapy; CI, confidence interval; cp/ml, copies per milliliter; DNA, deoxyribonucleic acid; HBcrAg, hepatitis B core-related antigen; HBeAg, hepatitis B e antigen; HBV, hepatitis B virus; IU/l, international units per liter; IU/ml, international units per milliliter; OR, odds ratio; qHBsAg, quantitative hepatitis B surface antigen; RNA, ribonucleic acid; U/ml, units per milliliter.

Table S7: Determinants of HBsAg loss at the last follow-up visit: sensitivity analyses excluding participants with detectable hepatitis C RNA at baseline or ever replicating hepatitis D infection.

		**Univariable analysis**		**Multivariable analysis**			
	**N**	**OR (95% CI)**	**P-value**	**OR (95% CI)**	**P-value**	**OR (95% CI)**	**P-value**
**HBeAg-negative participants**							
				**with HBcrAg (N=252)**		**with HBV RNA (N=246)**	
qHBsAg ≤1000 IU/ml	266	6.38 (3.36-12.09)	**<0.001**	9.20 (4.29-19.74)	**<0.001**	8.48 (3.95-18.22)	**<0.001**
HBV DNA, per 1 $\log_{10}$ IU/ml increase	264	0.90 (0.78-1.04)	0.15	0.88 (0.71-1.09)	0.24	0.98 (0.79-1.23)	0.89
HBV RNA, per 1 $\log_{10}$ cp/ml increase	252	0.91 (0.75-1.09)	0.30			1.02 (0.77-1.36)	0.88
HBcrAg, per 1 $\log_{10}$ U/ml increase	259	1.02 (0.83-1.25)	0.85	1.33 (0.95-1.86)	0.10		
ALT, per 10 IU/ml increase	260	1.03 (1.00-1.07)	0.08	1.03 (0.99-1.08)	0.16	1.04 (1.00-1.09)	0.06
Age, per 10 years	266	1.25 (0.95-1.65)	0.11	1.14 (0.81-1.61)	0.45	1.18 (0.83-1.64)	0.38
Female sex at birth	266	0.77 (0.39-1.51)	0.45	1.30 (0.59-2.89)	0.52	1.28 (0.58-2.85)	0.54
CD4 <200 cells/ul	264	1.05 (0.48-2.27)	0.91	1.16 (0.43-3.10)	0.77	1.57 (0.58-4.23)	0.37
ART-experienced	266	0.97 (0.54-1.75)	0.93	0.67 (0.33-1.39)	0.29	0.75 (0.36-1.56)	0.44
Follow-up time, per 1 $\log_{10}$ years	266	2.43 (1.01-5.84)	**0.05**	2.53 (0.93-6.87)	0.07	2.55 (0.94-6.93)	0.07
**HBeAg-positive participants**							
				**with HBcrAg (N=247)**		**with HBV RNA (N=234)**	
qHBsAg ≤1000 IU/ml	258	5.05 (2.05-12.42)	**<0.001**	4.72 (1.36-16.32)	**0.01**	4.99 (1.47-16.96)	**0.01**
HBV DNA, per 1 $\log_{10}$ IU/ml increase	257	1.06 (0.92-1.22)	0.39	1.29 (1.04-1.59)	**0.02**	1.38 (1.10-1.74)	**0.006**
HBV RNA, per 1 $\log_{10}$ cp/ml increase	239	0.73 (0.60-0.88)	**0.001**			0.66 (0.49-0.89)	**0.006**
HBcrAg, per 1 $\log_{10}$ U/ml increase	253	0.73 (0.55-0.96)	**0.03**	0.73 (0.49-1.09)	0.13		
ALT, per 10 IU/ml increase	252	0.99 (0.97-1.02)	0.64	1.00 (0.97-1.02)	0.71	1.00 (0.97-1.02)	0.73
Age, per 10 years	258	1.10 (0.73-1.65)	0.65	1.28 (0.80-2.04)	0.30	1.32 (0.83-2.10)	0.24
Female sex at birth	258	2.31 (0.84-6.32)	0.10	2.23 (0.67-7.45)	0.19	2.04 (0.59-7.06)	0.26
CD4 <200 cells/ul	258	1.66 (0.76-3.59)	0.20	1.03 (0.40-2.68)	0.95	1.12 (0.42-2.95)	0.83
ART-experienced	258	0.38 (0.19-0.76)	**0.006**	0.46 (0.21-1.04)	0.06	0.54 (0.24-1.25)	0.15
Follow-up time, per 1 $\log_{10}$ years	258	3.72 (1.17-11.79)	**0.03**	3.50 (1.00-12.21)	**0.05**	2.68 (0.74-9.70)	0.13

The determinants of HBsAg loss were assessed using univariable and multivariable logistic regression models. Wald tests were performed to assess statistical significance of individual covariables with a significance being defined as p<0.05 (bold). Abbreviations: ALT, alanine aminotransferase; ART, antiretroviral therapy; CI, confidence interval; cp/ml, copies per milliliter; DNA, deoxyribonucleic acid; HBcrAg, hepatitis B core-related antigen; HBeAg, hepatitis B e antigen; HBV, hepatitis B virus; IU/ml, IU/l, international units per liter; international units per milliliter; OR, odds ratio; qHBsAg, quantitative hepatitis B surface antigen; RNA, ribonucleic acid; U/ml, units per milliliter.

Table S8: Determinants of HBsAg loss after two years: sensitivity analyses excluding participants with detectable hepatitis C RNA at baseline or ever replicating hepatitis D infection.

	N	Univariable analysis		Multivariable analysis			
		OR (95% CI)	P-value	OR (95% CI)	P-value	OR (95% CI)	P-value
**HBeAg-negative participants**							
				**with HBcrAg (N=213)**		**with HBV RNA (N=207)**	
qHBsAg ≤1000 IU/ml	225	6.02 (2.61-13.89)	**<0.001**	18.16 (5.33-61.81)	**<0.001**	16.44 (4.88-55.33)	**<0.001**
HBV DNA, per 1 log$_{10}$ IU/ml increase	223	1.05 (0.89-1.23)	0.59	0.95 (0.72-1.25)	0.72	1.08 (0.83-1.41)	0.58
HBV RNA, per 1 log$_{10}$ cp/ml increase	212	1.07 (0.88-1.31)	0.48			1.36 (0.95-1.95)	0.10
HBcrAg, per 1 log$_{10}$ U/ml increase	219	1.22 (0.97-1.53)	0.09	1.87 (1.20-2.93)	**0.006**		
ALT, per 10 IU/ml increase	219	1.04 (1.00-1.08)	**0.03**	1.04 (0.99-1.08)	0.09	1.25 (1.01-1.09)	**0.02**
Age, per 10 years	225	1.18 (0.82-1.71)	0.37	1.26 (0.79-2.02)	0.33	1.25 (0.79-1.99)	0.33
Female sex at birth	225	1.72 (0.78-3.80)	0.18	6.19 (2.04-18.76)	**0.001**	5.40 (1.80-16.17)	**0.003**
CD4 <200 cells/ul	224	0.93 (0.33-2.61)	0.89	0.59 (0.16-2.19)	0.43	0.89 (0.25-3.22)	0.86
ART-experienced	225	0.97 (0.45-2.07)	0.93	0.83 (0.31-2.22)	0.71	1.01 (0.37-2.71)	0.99
**HBeAg-positive participants**							
				**with HBcrAg (N=210)**		**with HBV RNA (N=199)**	
qHBsAg ≤1000 IU/ml	220	4.02 (1.39-11.65)	**0.01**	1.31 (0.31-5.57)	0.72	1.81 (0.45-7.30)	0.41
HBV DNA, per 1 log$_{10}$ IU/ml increase	219	0.92 (0.78-1.08)	0.31	1.05 (0.84-1.32)	0.66	1.09 (0.86-1.38)	0.48
HBV RNA, per 1 log$_{10}$ cp/ml increase	203	0.64 (0.51-0.79)	**<0.001**			0.65 (0.48-0.89)	**0.006**
HBcrAg, per 1 log$_{10}$ U/ml increase	215	0.60 (0.44-0.83)	**0.002**	0.62 (0.40-0.96)	**0.03**		
ALT, per 10 IU/ml increase	215	1.00 (0.98-1.03)	0.75	1.01 (0.99-1.03)	0.48	1.01 (0.99-1.03)	0.55
Age, per 10 years	220	0.92 (0.55-1.53)	0.75	1.10 (0.62-1.94)	0.75	1.15 (0.65-2.02)	0.64
Female sex at birth	220	2.25 (0.69-7.39)	0.18	2.35 (0.58-9.44)	0.23	2.03 (0.49-8.34)	0.33
CD4 <200 cells/ul	220	1.34 (0.50-3.60)	0.56	0.66 (0.19-2.21)	0.50	0.72 (0.21-.247)	0.61
ART-experienced	220	0.25 (0.10-0.62)	**0.003**	0.26 (0.09-0.70)	**0.008**	0.33 (0.12-0.92)	**0.03**

The determinants of HBsAg loss were assessed using univariable and multivariable logistic regression models. Wald tests were performed to assess statistical significance of individual covariables with significance being defined as p<0.05 (bold). Abbreviations: ALT, alanine aminotransferase; ART, antiretroviral therapy; CI, confidence interval; cp/ml, copies per milliliter; DNA, deoxyribonucleic acid; HBcrAg, hepatitis B core-related antigen; HBeAg, hepatitis B e antigen; HBV, hepatitis B virus; IU/ml, IU/l, international units per liter; international units per milliliter; OR, odds ratio; qHBsAg, quantitative hepatitis B surface antigen; RNA, ribonucleic acid; U/ml, units per milliliter.

Table S9: Characteristics of participants with qHBsAg <0.05 IU/ml after two years of tenofovir therapy, stratified by qHBsAg level at last follow-up visit.

	**qHBsAg level at last follow-up**		
	**qHBsAg <0.05 IU/ml N=47**	**qHBsAg ≥0.05 IU/ml N=16**	**p-value**
**Demographical and clinical characteristics at tenofovir start**			
Female sex at birth	11/47 (23.4%)	4/16 (25.0%)	0.90
Age, median (IQR), years	43.0 (34.0-51.0)	39.5 (36.5-41.5)	0.22
Calendar year of tenofovir start, median (IQR)	2008 (2004-2010)	2006 (2004-2009)	0.31
Follow-up duration, median (IQR), years	9.7 (6.5-14.0)	11.6 (8.0-15.6)	0.21
HBV prevalence ≥2% in country of origin*	11/47 (23.4%)	4/16 (25.0%)	0.90
BMI, median (IQR), kg/m^2	23.0 (20.1-24.8)	23.5 (20.2-24.9)	0.78
CD4 count, median (IQR), $cells/mm^3$	345.0 (198.0-510.0)	317.0 (242.5-432.5)	0.63
CD4 nadir, median (IQR), $cells/mm^3$	145.0 (83.0-239.0)	211.0 (74.5-274.5)	0.54
CD4/CD8 ratio, median (IQR)	0.4 (0.2-0.7)	0.3 (0.2-0.3)	0.09
HIV viral load <50 cp/ml	21/47 (44.7%)	6/16 (37.5%)	0.62
Stage C†	12/47 (25.5%)	4/16 (25.0%)	0.97
Liver cirrhosis	4/27 (14.8%)	1/10 (10.0%)	0.70
Ever unhealthy alcohol use	1/19 (5.3%)	0/7 (0.0%)	0.54
**Laboratory characteristics at tenofovir start**			
ALT, median (IQR), IU/l	44.0 (28.0-73.0)	33.0 (21.0-56.0)	0.14
ALT ≥5x ULN	5/45 (11.1%)	0/14 (0.0%)	0.19
HBV DNA, median (IQR), log_{10} IU/ml]	3.3 (0.0-5.7)	3.7 (2.1-7.4)	0.30
HBV DNA <20 IU/ml	15/47 (31.9%)	3/16 (18.8%)	0.31
qHBsAg, median (IQR), log_{10} IU/ml	1.4 (0.1-3.5)	3.7 (3.3-4.4)	**0.007**

>1000 IU/ml	17/47 (36.2%)	13/16 (81.3%)	
10-1000 IU/ml	9/47 (19.1%)	1/16 (6.3%)	
≤10 IU/ml	21/47 (44.7%)	2/16 (12.5%)	
HBcrAg, median (IQR), log_{10} U/ml	5.3 (3.0-7.5)	6.7 (3.7-7.9)	0.28
HBcrAg <3 log_{10} U/ml	11/47 (23.4%)	3/15 (20.0%)	0.78
HBV RNA, median (IQR), log_{10} copies/ml	0.8 (0.0-4.8)	4.9 (0.8-5.6)	**0.02**
HBV RNA <LLOD	23/47 (48.9%)	1/15 (6.7%)	**0.003**
HBeAg-positive	16/46 (34.8%)	10/15 (66.7%)	**0.03**
**Laboratory characteristics after 2 years of FUP**			
ALT, median (IQR), IU/l	27.0 (17.0-38.0)	21.0 (16.5-40.0)	0.65
ALT ≥5x ULN	1/47 (2.1%)	2/16 (12.5%)	0.09
HBV DNA, median (IQR), log_{10} IU/ml]	0.0 (0.0-0.0)	0.0 (0.0-1.7)	**0.03**
HBV DNA <20 IU/ml	44/45 (97.8%)	11/16 (68.8%)	**<0.001**
HBcrAg, median (IQR), log_{10} U/ml	2.9 (2.9-3.7)	2.9 (2.9-4.6)	0.33
HBcrAg <3 log_{10} U/ml	30/47 (63.8%)	9/16 (56.3%)	0.59
HBV RNA, median (IQR), log_{10} copies/ml	0.0 (0.0-0.0)	0.0 (0.0-1.3)	**<0.001**
HBV RNA <LLOD	46/47 (97.9%)	10/16 (62.5%)	**<0.001**
**Treatment-related characteristics**			
Started on TAF	1/47 (2.1%)	0/16 (0.0%)	0.56
Tenofovir interruption >30 days from baseline to 2 years FUP	4/47 (8.5%)	2/16 (12.5%)	0.64
Ever tenofovir therapy interruption >30 days	17/47 (36.2%)	6/16 (37.5%)	0.92
FUP time on tenofovir from baseline to 2 years FUP , median (IQR), %	100.0 (100.0-100.0)	100.0 (100.0-100.0)	0.96
FUP time on tenofovir from baseline to last FUP, median (IQR), %	100.0 (90.9-100.0)	100.0 (89.0-100.0)	0.91
Started with TAF	1/47 (2.1%)	0/16 (0.0%)	0.56
Switched from TDF to TAF during tenofovir	27/47 (57.4%)	9/16 (56.3%)	0.93

Lamivudine and/or emtricitabine co-therapy	46/47 (97.8%)	15/16 (93.8%)	0.42
Pegylated interferon-α co-therapy	4/47 (8.5%)	0/16 (0.0%)	0.23
Median CD4 count change from baseline to 2 years FUP, median (IQR), cells/mm^3	85.0 (4.0-227.0)	78.5 (-21.0-169.5)	0.28
Median CD4 count change from baseline to last FUP, median (IQR), cells/mm^3	299.0 (90.0-452.0)	251.5 (17.0-377.5)	0.48
Time with unsuppressed HIV viral load from baseline to 2 years FUP, median (IQR), %	13.0 (0.0-27.4)	13.1 (7.5-30.9)	0.34
Time with unsuppressed HIV viral load from baseline to last FUP, median (IQR), %	4.0 (1.1-14.6)	4.9 (2.1-7.2)	0.54
Worst reported level of adherence during first 2 years FUP			0.58
low adherence (<70%)	1/45 (2.2%)	0/14 (0.0%)	
partial adherence	1/45 (2.2%)	1/14 (7.1%)	
full adherence (>95%)	43/45 (95.6%)	13/14 (92.9%)	
Worst ever reported level of adherence during FUP			0.29
low adherence (<70%)	5/46 (10.9%)	3/14 (21.4%)	
partial adherence	5/46 (10.9%)	3/14 (21.4%)	
full adherence (>95%)	36/46 (78.3%)	8/14 (57.1%)	

*Based on 2015 estimates from GBD 2019 Hepatitis Collaborators, Lancet Gastroenterol Hepatol 2022; 7:796-829. †According to the clinical classification of HIV disease by the US Centers for Disease Control and Prevention. Data are presented as median (IQR) for continuous measures, and n/total (%) for categorical measures. Continuous variables were compared using Wilcoxon rank-sum tests. Comparisons of categorical variables were performed using Pearson's $\chi 2$ tests. Level of significance: $p<0.05$ (bold). Abbreviations: ALT, alanine aminotransferase; BMI, body mass index; DNA, deoxyribonucleic acid; FUP, follow-up; HBcrAg, hepatitis B core-related antigen; HBeAg, hepatitis B e antigen; HBV, hepatitis B virus; IQR, interquartile range; qHBsAg, quantitative hepatitis B surface antigen; RNA, ribonucleic acid; TAF, tenofovir alafenamide; TDF, tenofovir disoproxil fumarate; ULN, upper limit of normal.
